# Functional analysis of a gene-edited mouse model to gain insights into the disease mechanisms of a titin missense variant

**DOI:** 10.1007/s00395-021-00853-z

**Published:** 2021-02-26

**Authors:** He Jiang, Charlotte Hooper, Matthew Kelly, Violetta Steeples, Jillian N. Simon, Julia Beglov, Amar J. Azad, Lisa Leinhos, Pauline Bennett, Elisabeth Ehler, Jacinta I. Kalisch-Smith, Duncan B. Sparrow, Roman Fischer, Raphael Heilig, Henrik Isackson, Mehroz Ehsan, Giannino Patone, Norbert Huebner, Benjamin Davies, Hugh Watkins, Katja Gehmlich

**Affiliations:** 1 Division of Cardiovascular Medicine, Radcliffe Department of Medicine and British Heart Foundation Centre of Research Excellence Oxford, University of Oxford, Oxford, OX3 9DU UK; 2 Randall Centre for Cell and Molecular Biophysics, School of Cardiovascular Medicine and Sciences, King’s College London BHF Centre of Research Excellence, London, UK; 3 Department of Physiology, Anatomy and Genetics, University of Oxford, Oxford, UK; 4 Nuffield Department of Clinical Medicine, Target Discovery Institute, University of Oxford, Oxford, UK; 5 Department of Medical Sciences, Cardiology, Uppsala University, Uppsala, Sweden; 6 Department of Medical Cell Biology, Integrative Physiology, Uppsala University, Uppsala, Sweden; 7 Max Delbrueck Centre for Molecular Medicine, Berlin, Germany; 8 Transgenic Core, Wellcome Centre for Human Genetics, University of Oxford, Oxford, UK; 9 Institute of Cardiovascular Sciences, University of Birmingham, Birmingham, B15 2TT UK

**Keywords:** Mouse model, Titin missense variant, Telethonin, Proteo-toxic response, Cardiomyopathy, Proteasome

## Abstract

Titin truncating variants are a well-established cause of cardiomyopathy; however, the role of titin missense variants is less well understood. Here we describe the generation of a mouse model to investigate the underlying disease mechanism of a previously reported titin A178D missense variant identified in a family with non-compaction and dilated cardiomyopathy. Heterozygous and homozygous mice carrying the titin A178D missense variant were characterised in vivo by echocardiography. Heterozygous mice had no detectable phenotype at any time point investigated (up to 1 year). By contrast, homozygous mice developed dilated cardiomyopathy from 3 months. Chronic adrenergic stimulation aggravated the phenotype. Targeted transcript profiling revealed induction of the foetal gene programme and hypertrophic signalling pathways in homozygous mice, and these were confirmed at the protein level. Unsupervised proteomics identified downregulation of telethonin and four-and-a-half LIM domain 2, as well as the upregulation of heat shock proteins and myeloid leukaemia factor 1. Loss of telethonin from the cardiac Z-disc was accompanied by proteasomal degradation; however, unfolded telethonin accumulated in the cytoplasm, leading to a proteo-toxic response in the mice.We show that the titin A178D missense variant is pathogenic in homozygous mice, resulting in cardiomyopathy. We also provide evidence of the disease mechanism: because the titin A178D variant abolishes binding of telethonin, this leads to its abnormal cytoplasmic accumulation. Subsequent degradation of telethonin by the proteasome results in proteasomal overload, and activation of a proteo-toxic response. The latter appears to be a driving factor for the cardiomyopathy observed in the mouse model.

**Supplementary Information:**

The online version contains supplementary material available at 10.1007/s00395-021-00853-z.

## Introduction

Titin is the largest protein, with a size of up to 3.7 megadaltons. It is expressed specifically in striated muscle tissues [32]. Each titin molecule spans half a sarcomere, which is the contractile unit responsible for force generation in cardiomyocytes and skeletal myotubes: the N-terminus of titin is anchored at the Z-disc, while its C-terminal end is embedded in the M-band. Consistent with its enormous size, numerous proteins have been identified as titin binding partners. Examples include alpha-actinin, cysteine rich protein 3 (Csrp3, also called muscle LIM protein, MLP) and telethonin (also called Tcap) at the Z-disc; and myosin binding protein C, myosin and myomesin in more distal regions (for review see [15]). Importantly, several titin binding partners, including four-and-a-half-LIM-domain 1 and 2 (Fhl1 and Fhl2), cardiac ankyrin repeat protein (Ankrd1), heat shock proteins, obscurin and calpain-3, have been implicated in diverse signalling pathways [36]. This suggests that titin does not merely play a structural role as a ‘molecular ruler’ of the sarcomere [55] and modulator of passive stiffness [41], but it is also an important hub, integrating distinct signalling networks required for striated muscle adaptation (reviewed in [34]). In support of this, titin features a kinase domain [45] and is also subjected to post-translational modifications that modulate its functions (reviewed in [33]).

Titin is encoded by the gene *TTN*, comprising of 364 exons. Historically, *TTN* has been implicated in inherited skeletal or cardiac muscle diseases, mainly through genome-wide linkage approaches [14]. Advances in high throughput sequencing have made *TTN* more accessible for genetic analyses in clinical practice, revealing that up to 25% of familial dilated cardiomyopathy (DCM) cases are associated with truncating variants in *TTN* [21].

Due to the large size of the gene, individually rare, even private, missense variants in titin are seen extremely frequently in cohort databases, such as GnomAD [28]. *TTN* missense variants are detected in both cardiomyopathy cases and controls, hence it is assumed that the large majority do not cause disease [54]. As a result, *TTN* missense variants are generally ignored in clinical genetic reports, as their functional evaluation is so challenging that they are rarely actionable [27]. Nevertheless, using a combination of genome-wide linkage and next generation sequencing, we identified the titin missense variant A178D as the most likely cause of cardiac disease in a family affected by autosomal dominant left ventricular non-compaction (LVNC) and DCM [19]. In this family, 9 individuals carried the variant, and 4 of these had features of pronounced hypertrabeculation and mild DCM. A further 3 had only DCM and the remaining 2 had pronounced hypertrabeculation without DCM. Of note, 8 out of 9 affected individuals were clinically stable and did not progress into heart failure.

Here we present a unique mouse model carrying this variant, and show that, even in isolation, this variant is sufficient to cause cardiac disease in homozygous animals. Moreover, our molecular analyses give insight into the underlying disease mechanisms associated with this variant, namely the loss and proteasomal degradation of telethonin from the Z-discs of cardiomyocytes, the downregulation of Fhl2 and the induction of a proteo-toxic response. To our knowledge, this is the first mouse model for cardiac disease caused by a titin missense variant with unique insights into disease mechanisms.

## Methods

An expanded Methods section can be found in the Electronic Supplementary Material online.

### Ethical statement

Animal studies have been performed in accordance with the ethical standards laid down in the 1964 Declaration of Helsinki and its later amendments. Experimental procedures were performed in accordance with the Directive 2010/63/EU and UK Home office guidelines (project licences 30/2966, 30/2977, P37BA1809 and P572C7345) and approved by the respective institutional ethical review boards.

Animals were housed in specific pathogen free conditions, with the only reported positives on health screening over the entire time course of these studies being for *Tritrichomonas sp.* and *Entamoeba spp.* All animals were housed in social groups (apart from single housing with enrichment for chronic adrenergic stimulation experiments), provided with food and water ad libitum and, maintained on a 12 h light:12 h dark cycle (150–200 lx cool white LED light, measured at the cage floor).

Phenotyping experiments and offline analysis were performed blinded, and treatment groups were randomly assigned to animals, with no animals excluded from the study. All in vivo phenotyping studies were carried out using littermates of adult male mice. Both males and females were used for in vitro studies. Animals were sacrificed by cervical dislocation and death confirmed by cessation of circulation.

### Generation of the mice and genotyping

*Ttn* c. 533 C > A single base pair substitution was introduced into mouse embryonic stem cells using CRISPR-Cas9 mediated homology-directed repair. A target site for CRISPR mutagenesis (5′- AGCTCTTCCAACGCTGTTGG-3′), with minimal predicted off target sites (http://crispor.tefor.net) was selected, immediately downstream of the codon encoding Alanine-178, on the antisense strand relative to *Ttn* transcription. Two complementary oligos encoding this CRISPR target were annealed and cloned into the BbsI site of pX330-Puro, encoding a CAG-Cas9 expression cassette and a U6-promoter driven single guide-RNA (sgRNA) scaffold, together with a puromycin resistance cassette. A 138 nt single-stranded-oligodeoxynucleotide (ssODN), serving as a homology repair template, was synthesized (Eurogentec). This contained the desired A178D change and a silent 3 base pair change to introduce a de novo DdeI restriction site to facilitate the genotyping of the recombinant allele (Fig. S1a, b). Mouse C57BL/6N embryonic stem cells (ES, JM8F6) were electroporated with 5 µg of the pX330-Puro plasmid and 200 pmol of the ssODN using the Neon transfection system (Life Technologies, 3 × 1400 V, 10 ms). After 24 h, 48 h of selection in 600 ng/µl puromcyin was applied, and resistant ES cells were clonally expanded and genotyped using primers (5′- CCGAGACAGCACCACCCAACTT-3′ and 5′-TTGACCTTAGCTCAGGCGAGCACC-3′) to amplify the target region. Successful targeting was first established by DdeI digestion, followed by confirmation by Sanger sequencing. ES cell clones harbouring the A178D mutation homozygously were microinjected into albino C57BL/6 J blastocysts. The resulting chimeras were bred with wild-type C57BL/6 J mice and germline transmission of the A178D *Ttn* allele confirmed.

Both heterozygous (HET) and homozygous (referred to as A178D) titin A178D mice were viable and fertile. The expression of the mutated allele A178D was confirmed at the mRNA level by reverse transcriptase-PCR and Sanger sequencing (Fig. S1c), as well as at the protein level by mass spectrometry (Fig. S1d). Animals were backcrossed onto C57BL/6J (Envigo) for at least 2 generations before generating wild-type and heterozygous littermates and for at least 5 generations before generating wild-type and homozygous littermates for all WT and A178D studies.

### Ultrasound echocardiography

General anaesthesia was initially induced using 4% isoflurane by inhalation (Piramal Critical Care) in an anaesthesia chamber. Animals were then placed onto a heated platform, and the anaesthesia was maintained continuously with the usage of 1–1.5% isoflurane. Body temperature of the animals was maintained at 37 ºC, and echocardiography was performed with a 22–55 MHz linear array transducer using the Vevo 2100 ultrasound system (Visualsonics). Data acquisition was normally completed within 30 min of anaesthesia, followed by offline analysis blinded to the genotype and the treatment group, if applicable.

### Left ventricular haemodynamic measurements

To measure left ventricular (LV) haemodynamic index, general anaesthesia was induced as above. The animal was surgically prepared to allow insertion of a 1.4 F Millar Mikro-tip catheter (SPR-671) into the LV of the animal via the carotid artery and intubation of another infusion catheter of an electronic auto-pump into the jugular vein. Animals were maintained under 1.25–1.5% isoflurane for 15 min to allow baseline recordings, before administration of dobutamine (Hameln Pharmaceuticals Ltd) through the jugular vein (4 ng per g bodyweight per min, followed by 16 ng per g bodyweight per min) for the contractile reserve assessment.

### Administration of drugs via osmotic minipumps

Animals of each genotype were randomised into treatment and control groups. A short surgical procedure for implantation was carried out. Briefly, anaesthesia was induced as described above, and each animal was given a single dose of Vetergesic (buprenorphine hydrochloride, Ceva Animal Health Ltd) at 50 µg kg^−1^ body weight by sub-cutaneous injection for pain relief before the procedure. Micro-osmotic pumps (model 1002, ALZET) were implanted subcutaneously into continuously anaesthetised animals (with 1.5% isoflurane) via a midscapular incision. In the treatment group, the α- and β-adrenergic agonists, isoprenaline and phenylephrine (Iso/PE; in hydrochloride forms, Sigma-Aldrich) were delivered to the animals via minipumps that released both substances at a rate of 15 mg kg^−1^ body weight per day in 0.9% NaCl (Vetivex 1, from Dechra) for a total of 14 days. In the control group, animals were implanted with minipumps containing 0.9% NaCl alone for the same time course. The response of adrenergic challenge was assessed by echocardiography 13 days post-surgery, followed by organ harvest on day 14 to allow downstream analysis.

For tandem ubiquitin-binding entities (TUBE) assay (see below) and cryo-sections, epoxomicin (APexBio), was administered at 0.5 mg kg^−1^ bodyweight per day in 10% dimethyl sulfoxide in 0.9% NaCl for 7 days using micro-osmotic pumps (model 1007D, ALZET). Vehicle (10% dimethyl sulfoxide in 0.9% NaCl) served as control.

### Western blotting

Western blotting was performed as described [12] with antibodies listed in Table S11.

Please note some loading controls (Gapdh or Ponceau S) may be shown in multiple figures, as the same membranes were probed for more than one protein. This applies to Figs. 4b, 5a, 6a, S16–S20, S23, S30–S35.

### Mass spectrometry

Protein fractionation into cytoplasmic and myofilament fractions was done according to [59]. Sample preparation, data acquisition and analysis see Supplementary Methods.

### Cardiomyocyte isolation, contractility, calcium transients, size and immunofluorescence

Adult mouse left ventricular cardiomyocytes were isolated and sarcomere shortening measurements were obtained using an IonOptix µstep apparatus according to the manufacturer’s operating instructions as previously described [52]. For details see Supplementary Methods.

### High Resolution Episcopic Microscopy (HREM)

Tissue and data processing for HREM was performed as previously described [44]. For details see Supplementary Methods.

### Statistics

Values are given as mean ± standard error of mean (SEM) if normally distributed or as median ± SEM if not, or as violin or box and whisker plots displaying median, 25th/75th percentile, and whiskers as minimum/maximum values. To compare two unpaired sample groups, data was tested for normality using the Shapiro-Wilk test. Normally distributed data was analysed by Student's *t* test, and data that was not, was analysed by Mann–Whitney *U* test (GraphPad Prism 7.03).

When there were more than 2 groups and data were normally distributed, one-way ANOVA followed by Tukey’s post-hoc test was used (GraphPad Prism 7.03). If data were not normally distributed, *p* values were obtained by Kruskal–Wallis followed by Wilcoxon rank sum test with Bonferroni correction (R version 3.6.0), or by Kruskal–Wallis followed by Dunn’s multiple comparison’s test. Haemodynamic data were analysed by two-way-ANOVA with Bonferroni correction (GraphPad Prism 7.03). Violin plots were generated with GraphPad Prism 8.4.1.

To compensate for the likely clustering of data from cells from the same mouse, the Student’s *t* test using hierarchical clustering method described by Sikkel et al*.* was used to assess any difference in cardiomyocyte size, contractility and calcium measurements [50].

Annotations used: n.s. – not significant (*p* > 0.05), *p < 0.05, **p < 0.01, ***p < 0.001, ***p < 0.0001 versus WT; n indicates number of animals in each group. *p* < 0.05 was considered significant.

## Results

### Introduction of A178D does not affect titin expression or localisation

We first assessed whether the A178D missense variant change in our mouse model had any consequences on titin protein expression or localisation. No changes in titin transcript or protein levels were observed (Figs. S2 and S3a). Likewise, there was no alteration in isoform composition or global phosphorylation, the latter as assessed by ProQDiamond stain (Figs. S2b, S3c). Moreover, the T2 band, indicative of protein turnover [32], was similarly abundant between homozygous A178D and WT hearts (Figs. S2b, c, S3b). The titin epitope harbouring the variant (Z1Z2), localised normally to the Z-disc in homozygous A178D mice (Fig. S4). Likewise, titin epitopes close to the Z-disc (T12) and in the M-band (m8) were normally localised (Fig. S4). Finally, at the ultra-structural level, assessed by electron microscopy, sarcomeric and Z-disk appearance were normal and Z-disc width was unchanged in homozygous A178D hearts (Fig. S5). These observations suggest that the A178D variant has no effect on gross sarcomeric organisation of the heart.

### Heterozygous titin A178D mice display no cardiac abnormalities

As the titin A178D variant caused autosomal dominant cardiomyopathy in patients [19], we first assessed the cardiac phenotype of heterozygous (HET) mice by echocardiography. Their hearts were structurally indistinguishable from wild-type (WT) littermates at all time points investigated (3 month, 6 month, 1 year, Fig. S6a and Table S1). Moreover, heart weight to tibial length was normal (Fig. S6b), and no differences were observed during invasive LV haemodynamic measurements, either at baseline or upon adrenergic stimulation (Fig. S6c, Table S2).

### Homozygous titin A178D mice show features of DCM but not of LVNC

**Fig. 1:**
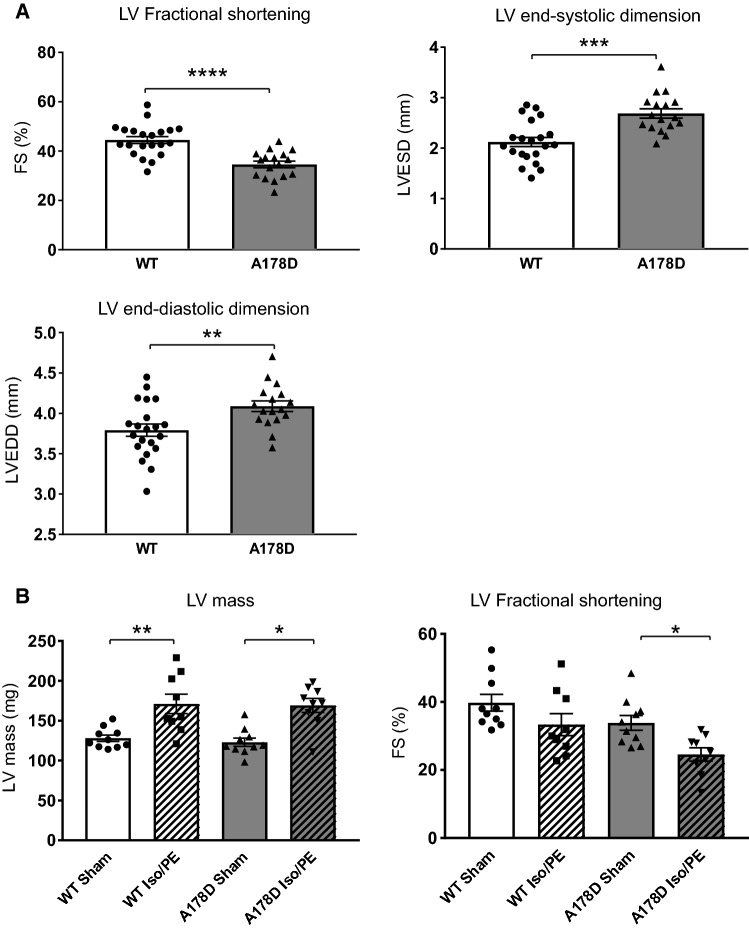
Cardiac phenotype of the A178D mice. **a** Echocardiographic measurements: fractional shortening, end-systolic and end-diastolic dimensions are shown (Student’s t-test, *n* = 21/17 WT/A178D, all males, age 99 ± 1 days). For cohort characteristics and a wider set of echocardiographic parameters please refer to Table S3. **b** Effect of chronic adrenergic stimulation by Iso/PE infusion on cardiac performance in WT and A178D mice. Hypertrophic response to Iso/PE as indicated by LV mass is similar between both genotypes (left). Systolic function of A178D hearts was reduced upon Iso/PE treatment (right). For cohort characteristics and a wider set of echocardiographic parameters please refer to Table S5; (*n* = 10/9 sham/Iso/PE; Kruskal–Wallis followed by Wilcoxon rank sum test with Bonferroni correction, all males, age 116–120 days)

The cardiac function of the homozygous A178D mice (referred to as A178D) in comparison to their WT littermates was investigated by echocardiography. A178D mice had mildly reduced systolic function (as shown by a reduction in fractional shortening) as well as enlarged systolic and diastolic dimensions (Fig. 1a, Table S3). By contrast, their diastolic wall thickness and left ventricular mass were not altered (Table S3) and heart weight normalised to tibial length was normal (Table S3). Thus, these mice show features typical of mild DCM.

We next probed for abnormal trabeculation, a hallmark of disease in a sub-set of patients carrying the *TTN* A178D variant [19]: High-resolution episcopic microscopy (HREM) suggested that A178D hearts were more rounded, with the lumens of both LV and RV appearing more open (Fig. S7a–n). However, morphometric analysis of lumen volume and heart shape was not significantly different between A178D and WT mice. In addition, trabeculation in basal sections of A178D hearts appeared more pronounced, although this was also not statistically significant (Fig. S7o). Thus, there was no clear evidence of a LVNC phenotype in A178D mice.

Titin is expressed not only in the heart, but also in skeletal muscle and pathogenic variants in titin are also associated with skeletal muscle pathologies [7]. However, there was no evidence of abnormalities in the skeletal muscle of A178D mice (Fig. S8).

### Ageing does not aggravate the phenotype of A178D mice

To test whether the phenotype of A178D becomes more pronounced with age, we aged a cohort of A178D mice to 1 year. No premature deaths were observed. On echocardiography, mice had mildly enlarged LV cavity dimensions in both systole and diastole (LVESD and LVESSD, Fig. S9, Table S4). The visual trend towards reduced fractional shortening did not reach significance. Hence, aged A178D did not have an aggravated cardiac phenotype, rather it appeared milder than in young mice.

### Adrenergic challenge aggravates the phenotype of A178D mice

We next compared the response of young A178D and WT mice to chronic adrenergic challenge, which has similar physiological consequences to trans-aortic constriction, but is more refined for the animals. Infusion with isoprenaline and phenylephrine (Iso/PE) led to a robust hypertrophic response in both WT and A178D hearts (Fig. 1b, Table S5). There was a tendency of reduced systolic function upon treatment, which was more profound in A178D hearts (Fig. 1b, Table S5).

In summary, homozygous A178D mice display a mild DCM phenotype without pronounced hypertrophy or abnormal trabeculation under baseline conditions, hence reflecting some, but not all aspects of the human cardiomyopathy phenotype. The observed in vivo phenotype does not aggravate with age. Their hypertrophic response to chronic adrenergic challenge did not differ from that of WT mice. However, challenged homozygous A178D hearts had a trend towards aggravated decline of cardiac function.

### Altered cellular characteristics in isolated A178D cardiomyocytes

**Fig. 2:**
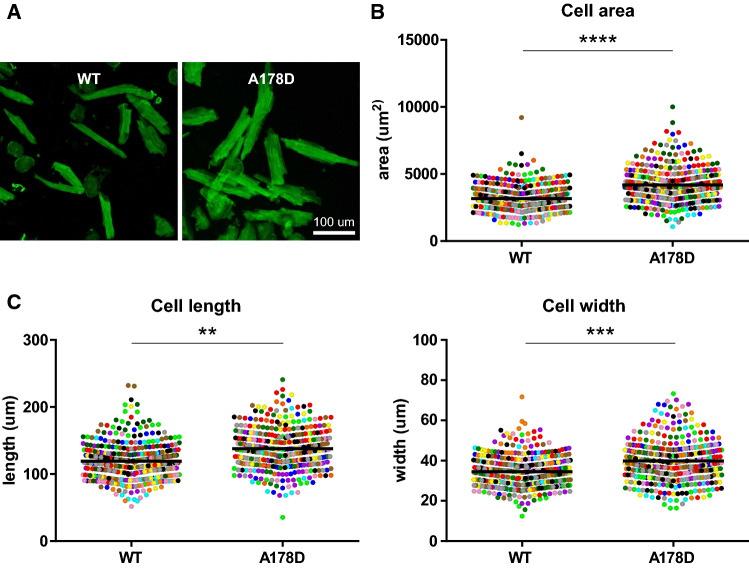
Altered cellular characteristics in A178D hearts. **a** Image of isolated cardiomyocytes from WT and A178D hearts, stained with wheat germ agglutinin (WGA). **b**, **c** Quantification of cellular dimensions: **b** cellular area, **c** left—cell length, right—cell width. Values of each measured cell are shown as individual data points, colour coded by the heart they are isolated from, means are indicated by black bar. (Total of 392/369 cells from 12 independent isolations from 4 female and 8 male mice each for WT/A178D, age: 128 ± 8 days/132 ± 10 days WT/A178D; Student’s *t* test using hierarchical clustering see Methods). For size distribution per isolation see Fig. S7, for summary of results Table S6

To assess the functional consequences of the A178D variant, we isolated adult mouse cardiomyocytes from WT and A178D hearts. Unloaded A178D cells had an approximate 32% increase in cell area, driven by increases in both cell length and width (Figs. 2, S10, Table S6).

Next, contractility of isolated unloaded cells was investigated upon pacing. All contraction parameters investigated were normal (Fig. S11a, Table S6). In agreement, measured calcium transients were normal (Fig. S11b, Table S7). These experiments suggest that the titin variant does not alter the contractile properties of isolated, unloaded cells.

We further assessed passive tension in demembranised, loaded fibre preparations (Fig. S12, Table S8). Overall passive tension, as well as titin-derived tension, were similar in WT and A178D mice, suggesting that the A178D change does not affect titin’s role in controlling compliance of the heart.

### Changes in the transcriptome profile of A178D mice

We next used transcriptome profiling to gain insight into the molecular changes underlying the titin A178D phenotype. We isolated RNA from 3 WT and 4 A178D hearts and analysed gene expression by RNAseq. 295 transcripts were up- and 374 were downregulated (Fig. S13, Table S9). In keeping with previous studies, genes known to be upregulated in DCM, including *Acta1* and *Nppa* [4, 20], were amongst the most upregulated genes (Fig. S13a). Gene set enrichment analysis identified ‘Proteasome’ to be the most significantly enriched pathway in A178D hearts (Fig. S13b, Table S10). Other enriched pathways were “Oxidative Phosphorylation”, “Butanoate Metabolism” and “Ribosome” (Table S10).

**Fig. 3:**
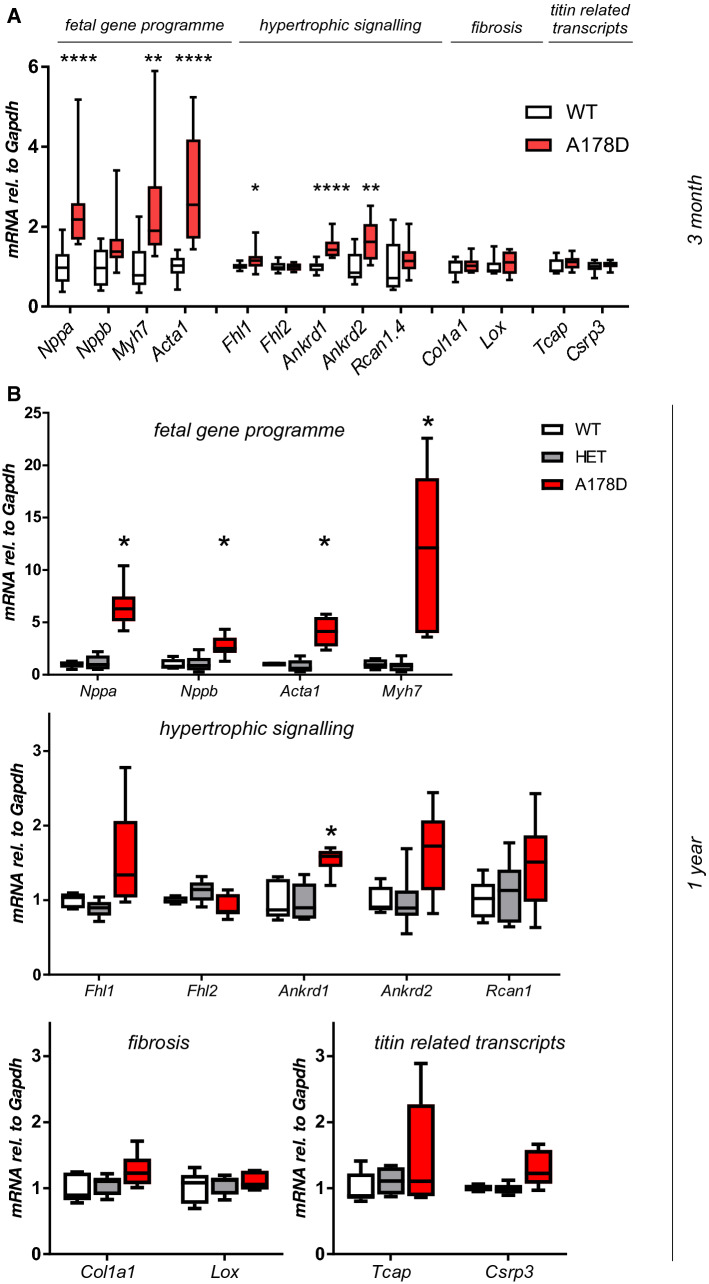
**a** Targeted assessment of transcriptional changes by qPCR for 3 month old A178D mice. All measurements are normalised to *Gapdh*. Significant changes are observed in the hearts of A178D mice (Mann–Whitney *U* test, WT: *n* = 10 (4M/6F), age 114.2 ± 0.7 days; A178D *n* = 12 (6M/6F), age 113.4 ± 0.5 days). **b** Targeted assessment of transcriptional changes by qPCR in 1 year old WT, Het and A178D mice. All measurements are normalised to *Gapdh*; Significant changes are observed in the hearts of A178D mice (Kruskal–Wallis followed by Dunn’s multiple comparison’s test, WT: *n* = 5, age 411 ± 3 days, HET *n* = 6, age 412 ± 2 days, A178D *n* = 6, age 423 ± 3 days, all male)

In agreement with the RNAseq data, a targeted approach revealed induction of the foetal gene programme in A178D hearts (induction of *Nppa, Myh7, Acta1,* Fig. 3a). Additionally, a very modest, but statistically significant induction of *Fhl1, Ankrd1* and *Ankrd2* was observed, all transcripts implicated in hypertrophic signalling. In the cohorts aged for 1 year, heterozygous mice displayed no indication of changes in expression for any transcript. Aged homozygous A178D mice showed induction of the foetal gene programme and statistically significant induction of *Ankrd1* (Fig. 3b). The expression of titin Z-disc binding partners muscle LIM protein (*Csrp3*) and telethonin (*Tcap*) was normal at the transcript level (Figs. 3, S2) in all cohorts investigated. Further, there was no evidence of induction of pro-fibrotic signalling. In support, histology of A178D hearts was indistinguishable from WT samples, and no prominent fibrosis was observed (Fig. S14).

We next investigated the molecular response to chronic adrenergic stimulation (Fig. S15). As expected, the Iso/PE treatment led to the induction of transcripts related to the foetal gene programme, hypertrophic signalling and fibrosis in both WT and A178D mice. In agreement with the tendency towards worse performance of A178D hearts in vivo, there was a trend towards increased induction of these transcripts in the Iso/PE treated A178D hearts compared to Iso/PE treated WT hearts, reaching statistical significance for *Nppa* and *Acta1*.

In summary, A178D hearts have a molecular signature of cardiomyopathy, namely the induction of a foetal gene programme, and this is aggravated upon adrenergic stimulation.

### Consequences of titin A178D at the protein level

To validate the findings of the transcript analysis, we probed young adult A178D mice, aged HET and homozygous A178D mice, and young adult A178D mice that underwent adrenergic challenge for protein levels of selected markers at the protein level: beta-myosin (encoded by *Myh7),* as a marker of the foetal gene programme; Fhl1, Ankrd1 and Rcan1, as markers of hypertrophic signalling; and Csrp3, as a titin binding partner in the Z-disc (Table 1, Figs. S16–S20). Beta-myosin was significantly upregulated in aged A178D mice, Fhl1 was significantly upregulated in the A178D sham group, while Rcan1 and Csrp3 were significantly upregulated in the A178D Iso/PE group. For Ankrd1, there was a visual trend of induction in aged A178D mice. These findings confirm that there was induction of the foetal gene programme and hypertrophic signalling at the protein level in our mouse model.

**Table 1:** Summary of results from Western blots on the three different cohorts

Protein (Transcript)	Figure	3 month old	Aged (1 year old)	Iso/PE challenge
Beta-myosin (*Myh7*)	S16	n.s.	↑	n.s.
Fhl1	S17	Not detectable	n.s.	↑ (sham)
Ankrd1	S18	Not quantifiable	Not quantifiable	n.s
Rcan1	S19	n.s.	n.s.	↑ (Iso/PE)
Csrp3/MLP	S20	n.s.	n.s.	↑ (Iso/PE)

Significant changes of homozygous A178D compared to WT (of same treatment group for Iso/PE challenge) are indicated; ↑ upregulation, *n.s.* not significant. No changes were observed in aged heterozygous mice

For original blots and quantifications see Figs. S16–20

### Unsupervised proteomics reveal insights into changes in the A178D hearts

**Fig. 4:**
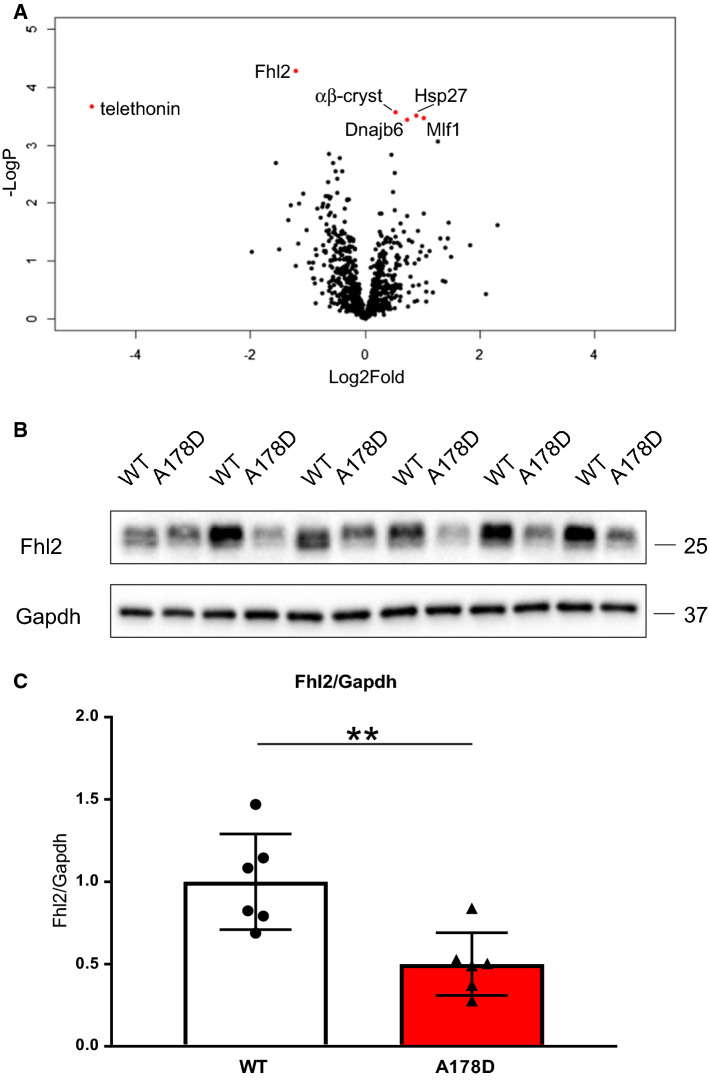
**a** Volcano plot visualising unsupervised proteomics results (normalised by log2 transformation followed by median subtraction in Perseus, followed by Student’s *t* test with permutation based FDR calculation, *n* = 6, age WT 114 ± 1 days/A178D 113 ± 1 days, 3M/3F). Significantly downregulated proteins are telethonin and Fhl2, significantly upregulated proteins are αβ-crystallin (αβ-cryst), heat shock protein 27 (Hsp27), Dnaj homology subfamily B member (Dnajb6, another heat shock protein) and myeloid leukaemia factor 1 (Mlf1), all shown in red. **b** Representative Western blot demonstrating the downregulation of Fhl2 in total extracts of A178D mice. Gapdh serves as loading control. **c** Quantification of blot in panel B indicates downregulation of Fhl2 by approximately 50%; (mean ± SEM, *n* = 6, age WT 114 ± 1 days/A178D 113 ± 1 days, 3M/3F, Student’s *t* test)

To gain further insights into the underlying disease mechanisms in our mouse model, we performed unsupervised proteomics to compare WT and A178D hearts. Quantitative comparison of myofilament protein fractions identified downregulation of telethonin and Fhl2 in A178D hearts, as well as upregulation of three heat shock proteins and myeloid leukaemia factor 1 (Mlf1) (Fig. 4a). We validated these results using Western blots, showing a 50% reduction in Fhl2 protein levels in A178D mice, both in total protein lysates (Fig. 4b, c) and in the myofilament fraction (Fig. S21C). Likewise, Mlf1 protein levels were upregulated in the adrenergic challenge cohort, both in the sham and in the Iso/PE group (Fig. S22). These experiments validate the findings from the proteomics approach for both proteins.

### Loss of telethonin from the Z-disc of A178D hearts

**Fig. 5:**
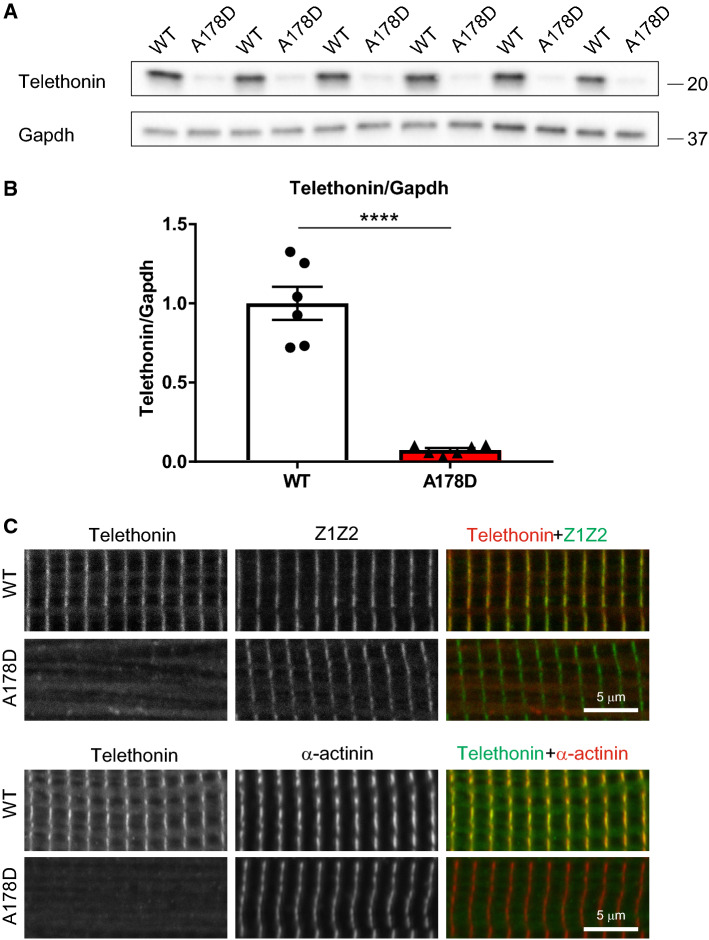
Loss of telethonin from the Z-discs of A178D hearts. **a** Protein destabilisation of telethonin in A178D hearts demonstrated by Western blotting. GAPDH serves as loading control. **b** Quantification of telethonin depletion (to < 10% of WT levels) in A178D hearts, normalised to Gapdh protein (shown as mean ± SEM, Student’s *t* test, *n* = 6, age 113 ± 1 days, all males). **c** Loss of telethonin from cardiac Z-disc in A178D mice. Top: Isolated cardiomyocytes from WT and A178D mice were stained with an antibody recognising titin Z1Z2 epitope and telethonin; merged images: Z1Z2 green, telethonin red. Bottom: cells were stained with a different telethonin antibody and Z-disc marker α-actinin (merged images: telethonin green, α-actinin red). Age WT: 98 days, A178D: 105 days, male)

In agreement with the proteomics data, telethonin was strikingly (> 90%) downregulated in A178D hearts (Fig. 5a, b), despite normal transcript levels (Fig. 3a). In WT adult mouse cardiomyocytes, telethonin co-localises with the titin Z1Z2 epitope at the Z-disc (Fig. 5c). By contrast, in A178D cardiomyocytes, the sarcomeric signal for telethonin at the Z-disc was completely abolished. This was confirmed using two antibodies that recognise different epitopes (Fig. 5c). Telethonin downregulation was also confirmed in homozygous A178D aged mice and in A178D mice that underwent adrenergic challenge experiments (Fig. S23b–d).

**Fig. 6:**
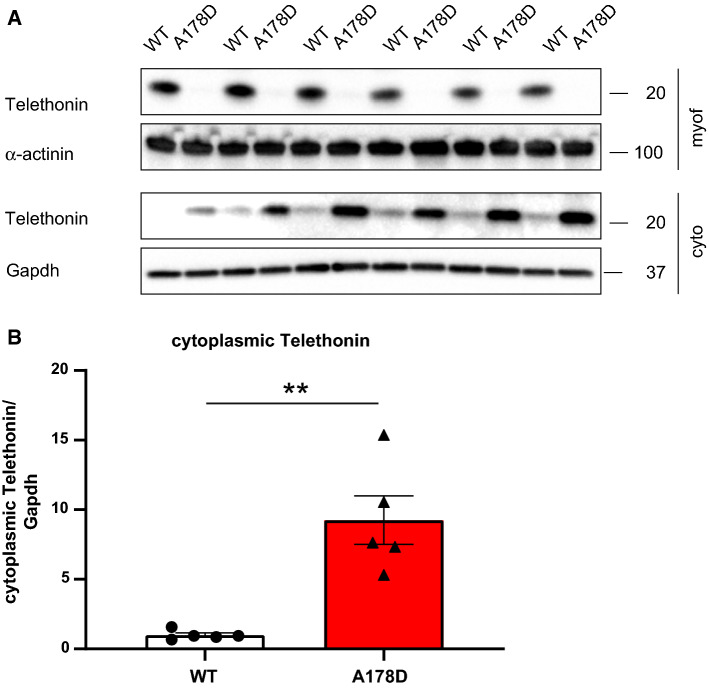
**a** Telethonin downregulation and re-distribution in A178D hearts: Myofilament and cytoplasmic protein fractions from WT and A178D hearts were blotted for telethonin (teleth). α-actinin served as loading control for the myofilament fraction, Gapdh as loading control for the cytoplasmic fraction, respectively. In A178D hearts, no telethonin is detected in the myofilament fraction. In contrast, the remaining telethonin protein is enriched in the cytoplasmic fraction. (*n* = 6, 3M/3F, age WT 114 ± 1 days, A178D 113 ± 1 days). **b** Quantification of telethonin accumulation in the cytoplasm in A178D hearts, normalised to Gapdh protein (shown as mean ± SEM, Student’s *t* test, *n* = 5, 2M/3F, age WT 114 ± 1 days, A178D 113 ± 1 days; the first two lanes of the blot in A were omitted for quantification)

**Fig. 7:**
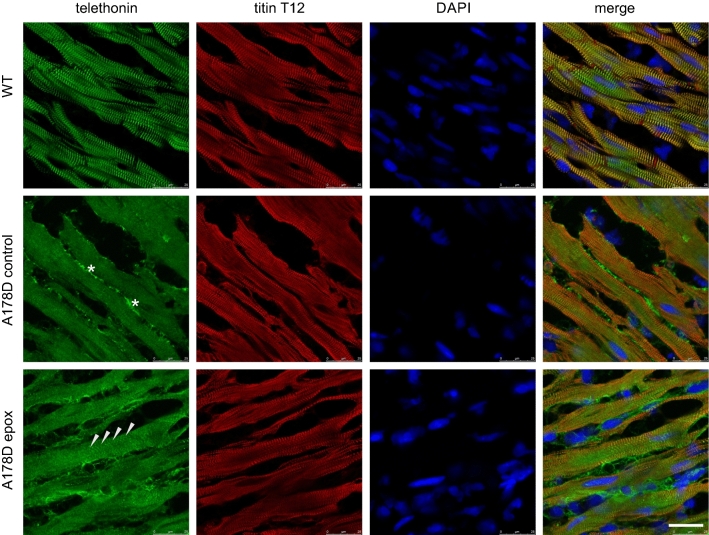
Loss of telethonin in A178D heart and partical restoration upon proteasomal inhibition. Top—Cryo-sections of hearts from WT mice, stained for telethonin and titin Z-disc epitope T12; nuclei are visualised with DAPI. Telethonin is localised at the Z-discs. Middle—same staining in A178D hearts treated with vehicle (10% dimethyl sulfoxide in 0.9% saline). Telethonin signal is lost from the Z-discs. Asterisks indicate non-specific staining of the telethonin antibody at the extracellular matrix. Bottom—A178D hearts treated with epoxomicin (0.5 mg kg^−1^ bodyweight per day for 7 days) show partial restoration of telethonin signal at the Z-disc (arrowheads). Scale bar represents 25 microns. All mice in this experiment are male (*n* = 2 per group; WT age 116 days, A178D age 115 ± 2 days)

Intriguingly, the small amount of remaining telethonin was not found in the myofilament fraction, but was re-distributed to the cytoplasmic protein fraction in A178D mice (Fig. 6), resulting in an approximately ninefold increase in *cytosolic* telethonin comparative to WT *cytosolic* levels. The protein was phosphorylated normally (Fig. S26a, b), but was polyubiquitinylated in A178D hearts (Fig. S23a), suggesting that it is degraded by the ubiquitin-proteasomal-system (UPS). Likewise, Fhl2 was also polyubiquitinylated in A178D hearts (Fig. S21b). Treatment of A178D mice with the proteasomal inhibitor epoxomicin in vivo partially restored telethonin and Fhl2 signal in A178D hearts (Figs. 7 and S24), adding confidence that the UPS is involved in the degradation of the proteins.

Protein degradation can also be mediated by autophagy [43]. Of two autophagy-markers investigated, LC3-II, the lipidated form of LC3, was normal (data not shown) and p62 elevated significantly in aged HET and A178D mice (Fig. S25).

The complete loss of telethonin from the Z-disc was the most striking feature of mouse hearts carrying the A178D variant. Therefore, we explored telethonin-mediated functions in A178D mice to determine if any of these were impaired. Telethonin has been suggested to be involved in p53 signalling, and lack of telethonin results in p53-mediated apoptosis [30]. However, p53 expression was normal at both transcript and protein levels in A178D hearts (Fig. S27a, b). In addition, most transcripts related to apoptosis were also normal in A178D hearts. This was true also for aged mice, or after chronic adrenergic stimulation (Fig. S27c, d). This is consistent with the observation that no replacement fibrosis (substituting apoptotic cardiomyocytes by fibrous tissue) was observed in these mice (Fig. S14). In addition, T-tubular organisation, linked to normal telethonin function [24], was unaltered in A178D mice (Fig. S26c). Hence, none of the known telethonin functions seemed affected in our mouse model.

### Proteo-toxic response in A178D hearts

**Fig. 8:**
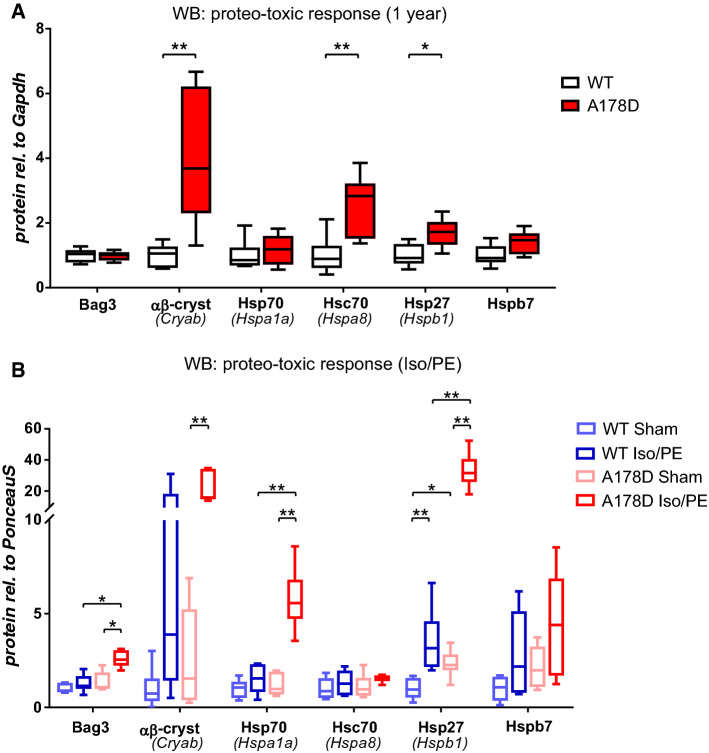
**a** Summary of induction of a proteo-toxic response in 1 year old A178D mice assessed by Western blotting (Mann–Whitney *U* test, *n* = 6, age WT 416 ± 2 days, A178D 423 ± 3 days, all male). **b** Summary of induction of a proteo-toxic response in a cohort undergoing adrenergic challenge assessed by Western blotting. Kruskal–Wallis followed by Wilcoxon rank sum test with Bonferroni correction, *n* = 6, age 120 ± 2 days, all male. For summary see Table 2 and for individual blots Figs. S29–34

With “proteasome” being the most upregulated pathway in our RNAseq analysis, incomplete clearance of cytosolic telethonin by the UPS could be an indicator of proteasomal overload. Consequently, accumulating mis-folded proteins could induce a proteo-toxic response [10]. In support of this, an upregulation of three heat shock proteins was detected in our proteomics dataset (Fig. 4a). Hence, we probed for the induction of the key players of the proteo-toxic response machinery. At transcript level, the only significant upregulation was observed for *Hspb1* and *Hspb7* in A178D hearts in the sham-treated group of the adrenergic challenge experiments (Fig. S28). At protein level, induction of a proteo-toxic response was more clearly indicated by upregulation of its components in aged A178D hearts (Hsc70, αβ-crystallin and Hsp27, Figs. 8a, S29–34, Table 2). The response was even more prominent upon adrenergic challenge: Bcl2-associated anthanogene (Bag3), Hsp70 and Hsp27 were strikingly upregulated in Iso/PE treated A178D hearts compared to WT undergoing the same treatment (Figs. 8b, S29–S34, Table 2).

**Table 2:** Summary of results from Western blots for proteo-toxic response on the three different cohorts

Protein (Transcript)	Figure	3 month old	Aged (1 year old)	Iso/PE challenge
Bag3	S29	n.s.	n.s.	↑ (Iso/PE)
Hsp70 (*Hspa1a*)	S30	n.s.	n.s.	↑ (Iso/PE)
Hsc70 (*Hspa8*)	S31	n.s.	↑ (also HET)	n.s.
αβ-crystallin (*Cryab*)	S32	n.s.	↑	n.s.
Hsp27 (*Hspb1*)	S33	n.s.	↑	
Hspb7	S34	n.s.	n.s.	n.s.

Significant changes of homozygous A178D compared to WT (of same treatment group for Iso/PE challenge) are indicated; ↑ upregulation, *n.s.* not significant

For original blots and quantifications see Figs. S29–34

In summary, telethonin was found to be lost from the Z-discs of titin A178D mice, however none of the known telethonin functions were affected in these mice. Furthermore, we demonstrated accumulation of the remaining telethonin in the cytoplasm, its ubiquitinylation and evidence of induction of a proteo-toxic response that was especially apparent upon additional stressors, such as ageing or adrenergic challenge.

## Discussion

In this study, we have generated a novel mouse model for the *TTN* missense variant A178D, which was originally identified as the most likely cause of cardiomyopathy in a family affected by both DCM and LVNC [19]. Remarkably, homozygosity for a single amino acid change in a protein of approximately 34,350 residues results in a measurable cardiac phenotype of reduced systolic function and mild dilatation as well as increased cardiomyocyte size. Moreover, while ageing did not aggravate the phenotype and we would expect a normal life-span of the mice, homozygous mice had an aggravated response to chronic adrenergic stimulation. Taken together, this is a robust indication that the titin A178D missense variant is solely responsible for the DCM aspect of cardiomyopathy in the reported family.

Our molecular studies, combining –omics approaches and targeted analyses, identify the potential molecular mechanisms underpinning the cardiomyopathy phenotype in this family (such as induction of transcripts related to the foetal gene programme) and provide further insights into disease pathways. However, despite the DCM phenotype, the gross performance of titin seemed to be unaffected by the missense variant: the titin protein has unchanged abundance and isoform composition and is normally incorporated into sarcomeres. Given the mild phenotype of our mouse model, it is no surprise that RNAseq identified only a modest number of differently expressed genes compared to other mouse models of cardiomyopathy [4, 10]. Likewise, the proteomics approach identified only 6 differentially expressed proteins.

Nevertheless, particularly the proteomics approach has revealed insights into the molecular changes driving disease in the model. Most strikingly, telethonin was downregulated in the A178D hearts. Telethonin is the only reported binding partner of the first two immunoglobulin domains of titin (Z1Z2), and the titin-telethonin interaction is the strongest known non-covalent protein–protein interaction [3]. Our previous work [19] has shown that A178D abolishes the titin-telethonin interaction in cells, and hence agrees with the observed loss of telethonin from the Z-discs of A178D mice. Titin Z1Z2, together with telethonin and Csrp3, was initially proposed to be a Z-disc-based mechano-sensor [29], but more recent work has suggested that Csrp3 is not an integral part of the Z-disc [13]; nevertheless, Csrp3 plays important roles in stress signalling [37]. In agreement with a functional titin Z1Z2—telethonin—Csrp3 complex, we see aggravated upregulation of Csrp3 in the A178D mice (with strikingly reduced telethonin levels) under adrenergic stress.

We now further demonstrate that unbound telethonin translocates to the cytoplasm and undergoes degradation. However, even the striking loss of telethonin (> 90% in our model) might be expected to only have a mild phenotype in mice, since a global knock-out of telethonin has no cardiac baseline phenotype in unchallenged mice [30]. To our surprise, none of the known telethonin features and suggested functions were affected in our model: our mouse model had normal passive stiffness [17], telethonin phosphorylation [5], normal T-tubular organisation [24], and no evidence of p53-mediated apoptosis [30].

Hence, a likely explanation could be that it is not the lack of telethonin at the Z-disc itself, but the remaining telethonin in the cytoplasm that is deleterious to the A178D hearts. Telethonin that is not complexed with titin is predicted to be unfolded [39] and cannot be recruited to the Z-disc. Instead it translocates to the cytoplasm, where it is likely to be recognised by small heat shock proteins (indicated by upregulation of αβ-crystallin and Hsp27), which try to maintain telethonin in a folded state and if this fails, target the protein for degradation by the UPS [25]. Degradation of telethonin by the UPS is supported by: (i) the presence of a PEST sequence in telethonin (amino acids 106–130 [23]). These are sequences rich in proline (P), glutamic acid (E), serine (S), and threonine (T), associated with proteins that have a short intracellular half-life and assumed to act as a signal peptide for protein degradation [47]; (i) the observed enrichment of a “Proteasome” gene set in our RNAseq dataset; (ii) the partial restoration of sarcomeric telethonin and Fhl2 in A178D mice treated with the proteasome inhibitor epoxomicin and (iii) the polyubiquitinylation of the protein in our mouse model, which we experimentally confirmed in a TUBE assay. However, the UPS fails to clear telethonin in the A178D mice to the cytoplasmic levels of WT mice. Such continuous demand on the UPS, as identified by upregulation of transcripts related to the proteasome in the RNAseq data set, can result in overload of the proteasome and in the accumulation of unfolded proteins. Indeed, we did document an upregulation of the proteo-toxic response machinery, as indicated by induction of its components Bag3, Hsc70 and Hsp70.

The importance of the proteo-toxic response machinery for cardiac integrity is clearly documented by human genetic data and experimental models: Human truncating variants in *BAG3* as well as loss-of-function missense variants (e.g. E455K) can cause DCM [9, 11]. Cardiac-specific loss of Bag3 results in cardiomyopathy in mice [11], and myofibrillar disruption in human induced pluripotent stem cell derived cardiomyocytes [26]. Moreover, the contribution of a proteo-toxic response to cardiomyopathy has been documented for pathogenic variants in *CSRP3* and *MYBPC3* through in vitro experiments and mouse models [10, 57].

How does this proteo-toxic response caused by UPS impairment result in cardiomyopathy? While the detailed mechanisms are not fully elucidated, three pathways have been proposed (reviewed in [49]): (i) under impaired UPS conditions, pro-hypertrophic and pro-apoptotic proteins may not be degraded and could accumulate, (ii) unfolded protein response may result in ER stress, upregulation of chaperones (heat shock proteins) and attenuation of protein synthesis, and (iii) UPS impairment may result in the activation of the autophagy-lysosomal pathway. Further work will focus on untangling these mechanisms.

Fhl2 does not bind titin at the affected titin Z1Z2 region, but in the more distal N2B region. Hence, its downregulation may be secondary, again possibly mediated through degradation by the UPS (as evidenced by polyubiquitinylation in the TUBE assay and partial restoration upon epoxomicin treatment). Fhl2 is an anti-hypertrophic signalling mediator [40], acting through sequestration of Erk1/2 and calcineurin [22, 46], and is also thought to recruit metabolic enzymes to the sarcomere [35]. However, despite these key roles in the heart, its loss is tolerated in the unchallenged mouse heart [31].

As is true for all model systems, genetically modified mice have their limitations for studying human genetic disease. Our mouse model reflects aspects of the human disease, albeit only in the homozygous setting, when wild-type titin protein is absent. By contrast, the human variant is heterozygous, thus the missense and wild-type protein co-exist. This caveat is commonly observed for mouse models of cardiomyopathy variants. For example, missense variants in *Mybpc3* and *Csrp3* only display phenotypes in the homozygous setting [10, 57], whilst heterozygous mice are normal. The vast physiological differences between mice and humans, as well as the sedentary lifestyle of laboratory mice, are likely to be contributing factors to this difference.

The mild DCM phenotype of our mouse model also mirrors the observation that young mice or rats carrying pathogenic truncating variants in titin display no overt DCM phenotypes [16, 48]. At transcript level, three of the four top KEGG pathways in our RNAseq dataset—“Ribosome”, “Oxidative Phosphorylation” and “Proteasome “—were also in the top four KEGG pathways to be found enriched in human cardiac biopsies from DCM patients with titin truncating variants, when compared to samples from DCM patients without titin truncating variants [56], suggesting that our titin missense model shares some molecular similarities with DCM caused by titin truncating variants, at least at the transcriptome level.

Careful morphological analysis by HREM failed to find evidence of abnormal trabeculation in the mice. Hence, the model does not recapitulate the hypertrabeculation in the reported family [19]. However, to our knowledge there are only few genetic mouse models for hypertrabeculation in the adult heart [6, 8], suggesting that this feature of human cardiac disease is poorly reflected in mice [42].

Our isolated cardiomyocyte work has underpinned features of cardiomyopathy in the mouse model, which appear to contradict the whole heart parameters: Isolated cardiomyocytes are increased in cell size (~ 30%), however we failed to detect significant increase in ventricular mass in the whole hearts. A possible explanation is that cardiomyocytes make up only 20–35% of the heart (by numbers) [2], and that the size difference observed in cells gets “diluted” in the whole heart.

To our surprise, contractility and calcium transient measurements in isolated cardiomyocytes were normal; they did not reflect the observed reduced systolic dysfunction documented in vivo by echocardiography. It is worth noting that the isolated cardiomyocytes are mechanically unloaded and hence cannot recapitulate all aspects of loaded heart performance, as demonstrated for other mouse models [38].

Titin phosphorylation was normal when assessed by ProQDiamond stain. However, this assay may not reflect all phosphorylation events equally [18] and titin functions can additionally be regulated by other post-translational events such as e.g. glutathionylation [1].

We cannot rule out a secondary contribution of autophagy to the degradation of telethonin and Fhl2, as has also been described for a *Mybpc3* mouse model [51]. Cross-talk between the UPS and autophagy is well established for the heart [60], with p62 being a crucial mediator linking both protein degrading systems [53].

Despite these limitations and caveats, our A178D mice represent a valuable tool to study titin-related cardiomyopathy. To our knowledge, it is the first mouse model of a cardiomyopathy-causing titin missense variant and provides valuable insights into the role of proteo-toxicity as a contributor to cardiomyopathy. Moreover, it will be of use in future studies to molecularly test, for example, the effects of environmental factors [58], oligogenic factors or drug therapy on disease outcome.

## Supplementary Information

Below is the link to the electronic supplementary material.

Supplementary file1 (PDF 20035 KB)

Supplementary file1 (XLSX 122 KB)

## Data Availability

The proteomics data underlying this article are available in PRIDE [https://www.ebi.ac.uk/pride/archive/], and can be accessed with identifier PXD020390. The RNAseq data are available on GEO [https://www.ncbi.nlm.nih.gov/geo/] and can be accessed with GSE154504. Other data underlying this article will be shared on reasonable request to the corresponding author.

## References

[1] Alegre-Cebollada J, Kosuri P, Giganti D, Eckels E, Rivas-Pardo JA, Hamdani N, Warren CM, Solaro RJ, Linke WA, Fernandez JM (2014). S-glutathionylation of cryptic cysteines enhances titin elasticity by blocking protein folding. Cell.

[2] Bergmann O, Zdunek S, Felker A, Salehpour M, Alkass K, Bernard S, Sjostrom SL, Szewczykowska M, Jackowska T, Dos Remedios C, Malm T, Andra M, Jashari R, Nyengaard JR, Possnert G, Jovinge S, Druid H, Frisen J (2015). Dynamics of Cell Generation and Turnover in the Human Heart. Cell.

[3] Bertz M, Wilmanns M, Rief M (2009). The titin-telethonin complex is a directed, superstable molecular bond in the muscle Z-disk. Proc Natl Acad Sci U S A.

[4] Burke MA, Chang S, Wakimoto H, Gorham JM, Conner DA, Christodoulou DC, Parfenov MG, DePalma SR, Eminaga S, Konno T, Seidman JG, Seidman CE (2016). Molecular profiling of dilated cardiomyopathy that progresses to heart failure. JCI Insight.

[5] Candasamy AJ, Haworth RS, Cuello F, Ibrahim M, Aravamudhan S, Kruger M, Holt MR, Terracciano CM, Mayr M, Gautel M, Avkiran M (2014). Phosphoregulation of the titin-cap protein telethonin in cardiac myocytes. J Biol Chem.

[6] Cao Q, Shen Y, Liu X, Yu X, Yuan P, Wan R, Liu X, Peng X, He W, Pu J, Hong K (2017). Phenotype and functional analyses in a transgenic mouse model of left ventricular noncompaction caused by a DTNA mutation. Int Heart J.

[7] Chauveau C, Rowell J, Ferreiro A (2014). A rising titan: TTN review and mutation update. Hum Mutat.

[8] Choquet C, Nguyen THM, Sicard P, Buttigieg E, Tran TT, Kober F, Varlet I, Sturny R, Costa MW, Harvey RP, Nguyen C, Rihet P, Richard S, Bernard M, Kelly RG, Lalevee N, Miquerol L (2018). Deletion of Nkx2-5 in trabecular myocardium reveals the developmental origins of pathological heterogeneity associated with ventricular non-compaction cardiomyopathy. PLoS Genet.

[9] Dominguez F, Cuenca S, Bilinska Z, Toro R, Villard E, Barriales-Villa R, Ochoa JP, Asselbergs F, Sammani A, Franaszczyk M, Akhtar M, Coronado-Albi MJ, Rangel-Sousa D, Rodriguez-Palomares JF, Jimenez-Jaimez J, Garcia-Pinilla JM, Ripoll-Vera T, Mogollon-Jimenez MV, Fontalba-Romero A, Garcia-Medina D, Palomino-Doza J, de Gonzalo-Calvo D, Cicerchia M, Salazar-Mendiguchia J, Salas C, Pankuweit S, Hey TM, Mogensen J, Barton PJ, Charron P, Elliott P, Garcia-Pavia P, Initiative EGCI (2018). Dilated cardiomyopathy due to BLC2-associated athanogene 3 (BAG3) mutations. J Am Coll Cardiol.

[10] Ehsan M, Kelly M, Hooper C, Yavari A, Beglov J, Bellahcene M, Ghataorhe K, Poloni G, Goel A, Kyriakou T, Fleischanderl K, Ehler E, Makeyev E, Lange S, Ashrafian H, Redwood C, Davies B, Watkins H, Gehmlich K (2018). Mutant muscle LIM Protein C58G causes cardiomyopathy through protein depletion. J Mol Cell Cardiol.

[11] Fang X, Bogomolovas J, Wu T, Zhang W, Liu C, Veevers J, Stroud MJ, Zhang Z, Ma X, Mu Y, Lao DH, Dalton ND, Gu Y, Wang C, Wang M, Liang Y, Lange S, Ouyang K, Peterson KL, Evans SM, Chen J (2017). Loss-of-function mutations in co-chaperone BAG3 destabilize small HSPs and cause cardiomyopathy. J Clin Invest.

[12] Gehmlich K, Dodd MS, Allwood JW, Kelly M, Bellahcene M, Lad HV, Stockenhuber A, Hooper C, Ashrafian H, Redwood CS, Carrier L, Dunn WB (2015). Changes in the cardiac metabolome caused by perhexiline treatment in a mouse model of hypertrophic cardiomyopathy. Mol Biosyst.

[13] Geier C, Gehmlich K, Ehler E, Hassfeld S, Perrot A, Hayess K, Cardim N, Wenzel K, Erdmann B, Krackhardt F, Posch MG, Osterziel KJ, Bublak A, Nägele H, Scheffold T, Dietz R, Chien KR, Spuler S, Fürst DO, Nürnberg P, Ozcelik C (2008). Beyond the sarcomere: CSRP3 mutations cause hypertrophic cardiomyopathy. Hum Mol Genet.

[14] Gerull B, Gramlich M, Atherton J, McNabb M, Trombitas K, Sasse-Klaassen S, Seidman JG, Seidman C, Granzier H, Labeit S, Frenneaux M, Thierfelder L (2002). Mutations of TTN, encoding the giant muscle filament titin, cause familial dilated cardiomyopathy. Nat Genet.

[15] Gigli M, Begay RL, Morea G, Graw SL, Sinagra G, Taylor MR, Granzier H, Mestroni L (2016). A review of the giant protein titin in clinical molecular diagnostics of cardiomyopathies. Front Cardiovasc Med.

[16] Gramlich M, Michely B, Krohne C, Heuser A, Erdmann B, Klaassen S, Hudson B, Magarin M, Kirchner F, Todiras M, Granzier H, Labeit S, Thierfelder L, Gerull B (2009). Stress-induced dilated cardiomyopathy in a knock-in mouse model mimicking human titin-based disease. J Mol Cell Cardiol.

[17] Granzier H, Labeit D, Wu Y, Labeit S (2002). Titin as a modular spring: emerging mechanisms for elasticity control by titin in cardiac physiology and pathophysiology. J Muscle Res Cell Motil.

[18] Hamdani N, Krysiak J, Kreusser MM, Neef S, Dos Remedios CG, Maier LS, Kruger M, Backs J, Linke WA (2013). Crucial role for Ca2(+)/calmodulin-dependent protein kinase-II in regulating diastolic stress of normal and failing hearts via titin phosphorylation. Circ Res.

[19] Hastings R, de Villiers CP, Hooper C, Ormondroyd L, Pagnamenta A, Lise S, Salatino S, Knight SJ, Taylor JC, Thomson KL, Arnold L, Chatziefthimiou SD, Konarev PV, Wilmanns M, Ehler E, Ghisleni A, Gautel M, Blair E, Watkins H, Gehmlich K (2016). Combination of whole genome sequencing, linkage, and functional studies implicates a missense mutation in titin as a cause of autosomal dominant cardiomyopathy with features of left ventricular noncompaction. Circ Cardiovasc Genet.

[20] Heinig M, Adriaens ME, Schafer S, van Deutekom HWM, Lodder EM, Ware JS, Schneider V, Felkin LE, Creemers EE, Meder B, Katus HA, Ruhle F, Stoll M, Cambien F, Villard E, Charron P, Varro A, Bishopric NH, George AL, Dos Remedios C, Moreno-Moral A, Pesce F, Bauerfeind A, Ruschendorf F, Rintisch C, Petretto E, Barton PJ, Cook SA, Pinto YM, Bezzina CR, Hubner N (2017). Natural genetic variation of the cardiac transcriptome in non-diseased donors and patients with dilated cardiomyopathy. Genome Biol.

[21] Herman DS, Lam L, Taylor MR, Wang L, Teekakirikul P, Christodoulou D, Conner L, DePalma SR, McDonough B, Sparks E, Teodorescu DL, Cirino AL, Banner NR, Pennell DJ, Graw S, Merlo M, Di Lenarda A, Sinagra G, Bos JM, Ackerman MJ, Mitchell RN, Murry CE, Lakdawala NK, Ho CY, Barton PJ, Cook SA, Mestroni L, Seidman JG, Seidman CE (2012). Truncations of titin causing dilated cardiomyopathy. N Engl J Med.

[22] Hojayev B, Rothermel BA, Gillette TG, Hill JA (2012). FHL2 binds calcineurin and represses pathological cardiac growth. Mol Cell Biol.

[23] Hutchinson KR, Saripalli C, Chung CS, Granzier H (2015). Increased myocardial stiffness due to cardiac titin isoform switching in a mouse model of volume overload limits eccentric remodeling. J Mol Cell Cardiol.

[24] Ibrahim M, Siedlecka U, Buyandelger B, Harada M, Rao C, Moshkov A, Bhargava A, Schneider M, Yacoub MH, Gorelik J, Knoll R, Terracciano CM (2013). A critical role for Telethonin in regulating t-tubule structure and function in the mammalian heart. Hum Mol Genet.

[25] Imai J, Yashiroda H, Maruya M, Yahara I, Tanaka K (2003). Proteasomes and molecular chaperones: cellular machinery responsible for folding and destruction of unfolded proteins. Cell Cycle.

[26] Judge LM, Perez-Bermejo JA, Truong A, Ribeiro AJ, Yoo JC, Jensen CL, Mandegar MA, Huebsch N, Kaake RM, So PL, Srivastava D, Pruitt BL, Krogan NJ, Conklin BR (2017). A BAG3 chaperone complex maintains cardiomyocyte function during proteotoxic stress. JCI Insight.

[27] Kalia SS, Adelman K, Bale SJ, Chung WK, Eng C, Evans JP, Herman GE, Hufnagel SB, Klein TE, Korf BR, McKelvey KD, Ormond KE, Richards CS, Vlangos CN, Watson M, Martin CL, Miller DT (2017). Recommendations for reporting of secondary findings in clinical exome and genome sequencing, 2016 update (ACMG SF v2.0): a policy statement of the American College of Medical Genetics and Genomics. Genet Med.

[28] Karczewski KJ, Francioli LC, Tiao G, Cummings BB, Alfoldi J, Wang Q, Collins RL, Laricchia KM, Ganna A, Birnbaum DP, Gauthier LD, Brand H, Solomonson M, Watts NA, Rhodes D, Singer-Berk M, England EM, Seaby EG, Kosmicki JA, Walters RK, Tashman K, Farjoun Y, Banks E, Poterba T, Wang A, Seed C, Whiffin N, Chong JX, Samocha KE, Pierce-Hoffman E, Zappala Z, O'Donnell-Luria AH, Minikel EV, Weisburd B, Lek M, Ware JS, Vittal C, Armean IM, Bergelson L, Cibulskis K, Connolly KM, Covarrubias M, Donnelly S, Ferriera S, Gabriel S, Gentry J, Gupta N, Jeandet T, Kaplan D, Llanwarne C, Munshi R, Novod S, Petrillo N, Roazen D, Ruano-Rubio V, Saltzman A, Schleicher M, Soto J, Tibbetts K, Tolonen C, Wade G, Talkowski ME, Genome Aggregation Database C, Neale BM, Daly MJ, MacArthur DG (2020). The mutational constraint spectrum quantified from variation in 141,456 humans. Nature.

[29] Knoll R, Hoshijima M, Hoffman HM, Person V, Lorenzen-Schmidt I, Bang ML, Hayashi T, Shiga N, Yasukawa H, Schaper W, McKenna W, Yokoyama M, Schork NJ, Omens JH, McCulloch AD, Kimura A, Gregorio CC, Poller W, Schaper J, Schultheiss HP, Chien KR (2002). The cardiac mechanical stretch sensor machinery involves a Z disc complex that is defective in a subset of human dilated cardiomyopathy. Cell.

[30] Knoll R, Linke WA, Zou P, Miocic S, Kostin S, Buyandelger B, Ku CH, Neef S, Bug M, Schafer K, Knoll G, Felkin LE, Wessels J, Toischer K, Hagn F, Kessler H, Didie M, Quentin T, Maier LS, Teucher N, Unsold B, Schmidt A, Birks EJ, Gunkel S, Lang P, Granzier H, Zimmermann WH, Field LJ, Faulkner G, Dobbelstein M, Barton PJ, Sattler M, Wilmanns M, Chien KR (2011). Telethonin deficiency is associated with maladaptation to biomechanical stress in the mammalian heart. Circ Res.

[31] Kong Y, Shelton JM, Rothermel B, Li X, Richardson JA, Bassel-Duby R, Williams RS (2001). Cardiac-specific LIM protein FHL2 modifies the hypertrophic response to beta-adrenergic stimulation. Circulation.

[32] Kruger M, Kotter S (2016). Titin, a central mediator for hypertrophic signaling, exercise-induced mechanosignaling and skeletal muscle remodeling. Front Physiol.

[33] Kruger M, Linke WA (2011). The giant protein titin: a regulatory node that integrates myocyte signaling pathways. J Biol Chem.

[34] Kruger M, Linke WA (2009). Titin-based mechanical signalling in normal and failing myocardium. J Mol Cell Cardiol.

[35] Lange S, Auerbach D, McLoughlin P, Perriard E, Schafer BW, Perriard JC, Ehler E (2002). Subcellular targeting of metabolic enzymes to titin in heart muscle may be mediated by DRAL/FHL-2. J Cell Sci.

[36] Lange S, Ehler E, Gautel M (2006). From A to Z and back? Multicompartment proteins in the sarcomere. Trends Cell Biol.

[37] Lange S, Gehmlich K, Lun AS, Blondelle J, Hooper C, Dalton ND, Alvarez EA, Zhang X, Bang ML, Abassi YA, Dos Remedios CG, Peterson KL, Chen J, Ehler E (2016). MLP and CARP are linked to chronic PKCalpha signalling in dilated cardiomyopathy. Nat Commun.

[38] Layland J, Grieve DJ, Cave AC, Sparks E, Solaro RJ, Shah AM (2004). Essential role of troponin I in the positive inotropic response to isoprenaline in mouse hearts contracting auxotonically. J Physiol.

[39] Lee EH, Gao M, Pinotsis N, Wilmanns M, Schulten K (2006). Mechanical strength of the titin Z1Z2-telethonin complex. Structure.

[40] Liang Y, Bradford WH, Zhang J, Sheikh F (2018). Four and a half LIM domain protein signaling and cardiomyopathy. Biophys Rev.

[41] Linke WA (2018). Titin gene and protein functions in passive and active muscle. Annu Rev Physiol.

[42] Luedde M, Ehlermann P, Weichenhan D, Will R, Zeller R, Rupp S, Muller A, Steen H, Ivandic BT, Ulmer HE, Kern M, Katus HA, Frey N (2010). Severe familial left ventricular non-compaction cardiomyopathy due to a novel troponin T (TNNT2) mutation. Cardiovasc Res.

[43] Mialet-Perez J, Vindis C (2017). Autophagy in health and disease: focus on the cardiovascular system. Essays Biochem.

[44] Mohun TJ, Weninger WJ (2012). Embedding embryos for high-resolution episcopic microscopy (HREM). Cold Spring Harb Protoc.

[45] Puchner EM, Alexandrovich A, Kho AL, Hensen U, Schafer LV, Brandmeier B, Grater F, Grubmuller H, Gaub HE, Gautel M (2008). Mechanoenzymatics of titin kinase. Proc Natl Acad Sci U S A.

[46] Purcell NH, Darwis D, Bueno OF, Muller JM, Schule R, Molkentin JD (2004). Extracellular signal-regulated kinase 2 interacts with and is negatively regulated by the LIM-only protein FHL2 in cardiomyocytes. Mol Cell Biol.

[47] Rogers S, Wells R, Rechsteiner M (1986). Amino acid sequences common to rapidly degraded proteins: the PEST hypothesis. Science.

[48] Schafer S, de Marvao A, Adami E, Fiedler LR, Ng B, Khin E, Rackham OJ, van Heesch S, Pua CJ, Kui M, Walsh R, Tayal U, Prasad SK, Dawes TJ, Ko NS, Sim D, Chan LL, Chin CW, Mazzarotto F, Barton PJ, Kreuchwig F, de Kleijn DP, Totman T, Biffi C, Tee N, Rueckert D, Schneider V, Faber A, Regitz-Zagrosek V, Seidman JG, Seidman CE, Linke WA, Kovalik JP, O'Regan D, Ware JS, Hubner N, Cook SA (2017). Titin-truncating variants affect heart function in disease cohorts and the general population. Nat Genet.

[49] Schlossarek S, Frey N, Carrier L (2014). Ubiquitin-proteasome system and hereditary cardiomyopathies. J Mol Cell Cardiol.

[50] Sikkel MB, Francis DP, Howard J, Gordon F, Rowlands C, Peters NS, Lyon AR, Harding SE, MacLeod KT (2017). Hierarchical statistical techniques are necessary to draw reliable conclusions from analysis of isolated cardiomyocyte studies. Cardiovasc Res.

[51] Singh SR, Zech ATL, Geertz B, Reischmann-Dusener S, Osinska H, Prondzynski M, Kramer E, Meng Q, Redwood C, van der Velden J, Robbins J, Schlossarek S, Carrier L (2017). Activation of autophagy ameliorates cardiomyopathy in Mybpc3-targeted knockin mice. Circ Heart Fail.

[52] Sparrow AJ, Sievert K, Patel S, Chang YF, Broyles CN, Brook FA, Watkins H, Geeves MA, Redwood CS, Robinson P, Daniels MJ (2019). Measurement of myofilament-localized calcium dynamics in adult cardiomyocytes and the effect of hypertrophic cardiomyopathy mutations. Circ Res.

[53] Su H, Wang X (2011). p62 Stages an interplay between the ubiquitin-proteasome system and autophagy in the heart of defense against proteotoxic stress. Trends Cardiovasc Med.

[54] Thomson KL, Ormondroyd E, Harper AR, Dent T, McGuire K, Baksi J, Blair E, Brennan P, Buchan R, Bueser T, Campbell C, Carr-White G, Cook S, Daniels M, Deevi SVV, Goodship J, Hayesmoore JBG, Henderson A, Lamb T, Prasad S, Rayner-Matthews P, Robert L, Sneddon L, Stark H, Walsh R, Ware JS, Farrall M, Watkins HC, Consortium NB-RD (2019). Analysis of 51 proposed hypertrophic cardiomyopathy genes from genome sequencing data in sarcomere negative cases has negligible diagnostic yield. Genet Med.

[55] Tskhovrebova L, Trinick J (2017). Titin and nebulin in thick and thin filament length regulation. Subcell Biochem.

[56] Verdonschot JAJ, Hazebroek MR, Derks KWJ, Barandiaran Aizpurua A, Merken JJ, Wang P, Bierau J, van den Wijngaard A, Schalla SM, Abdul Hamid MA, van Bilsen M, van Empel VPM, Knackstedt C, Brunner-La Rocca HP, Brunner HG, Krapels IPC, Heymans SRB (2018). Titin cardiomyopathy leads to altered mitochondrial energetics, increased fibrosis and long-term life-threatening arrhythmias. Eur Heart J.

[57] Vignier N, Schlossarek S, Fraysse B, Mearini G, Kramer E, Pointu H, Mougenot N, Guiard J, Reimer R, Hohenberg H, Schwartz K, Vernet M, Eschenhagen T, Carrier L (2009). Nonsense-mediated mRNA decay and ubiquitin-proteasome system regulate cardiac myosin-binding protein C mutant levels in cardiomyopathic mice. Circ Res.

[58] Ware JS, Amor-Salamanca A, Tayal U, Govind R, Serrano I, Salazar-Mendiguchia J, Garcia-Pinilla JM, Pascual-Figal DA, Nunez J, Guzzo-Merello G, Gonzalez-Vioque E, Bardaji A, Manito N, Lopez-Garrido MA, Padron-Barthe L, Edwards E, Whiffin N, Walsh R, Buchan RJ, Midwinter W, Wilk A, Prasad S, Pantazis A, Baski J, O'Regan DP, Alonso-Pulpon L, Cook SA, Lara-Pezzi E, Barton PJ, Garcia-Pavia P (2018). Genetic etiology for alcohol-induced cardiac toxicity. J Am Coll Cardiol.

[59] Yin X, Cuello F, Mayr U, Hao Z, Hornshaw M, Ehler E, Avkiran M, Mayr M (2010). Proteomics analysis of the cardiac myofilament subproteome reveals dynamic alterations in phosphatase subunit distribution. Mol Cell Proteom.

[60] Zheng Q, Su H, Tian Z, Wang X (2011). Proteasome malfunction activates macroautophagy in the heart. Am J Cardiovasc Dis.

