# Supplementary material for: Functional analysis of a gene-edited mouse model to gain insights into the disease mechanisms of a titin missense variant
Source: Basic Res Cardiol. 2021 Feb 26;116(1):14. doi: 10.1007/s00395-021-00853-z (PMC7910237; doi:10.1007/s00395-021-00853-z)
Supplement: Supplementary file 1 — Supplementary file1 (PDF 20035 KB) [file 395_2021_853_MOESM1_ESM.pdf]

**Functional analysis of a gene-edited mouse to gain insights into the disease mechanisms of a titin missense variant**

# Electronic Supplementary Material

He Jiang[1], Charlotte Hooper[1], Matthew Kelly[1], Violetta Steeples[1], Jillian N. Simon[1], Julia Beglov[1], Amar J. Azad[1], Lisa Leinhos[1], Pauline Bennett[2], Elisabeth Ehler[2], Jacinta I. Kalisch-Smith[3], Duncan B. Sparrow[3], Roman Fischer[4], Raphael Heilig[4], Henrik Isackson[5,6], Mehroz Ehsan[1], Giannino Patone[7], Norbert Huebner[7], Benjamin Davies[8], Hugh Watkins[1], Katja Gehmlich[1,9]

**Supplementary Methods**

*RNAseq*

LV tissue samples were powdered by freezing with liquid nitrogen and pulverised in a Biopulveriser (Stratech Scientific). 1 ml TRIzol Reagent (Invitrogen) was added to the tissue powder, mixed well and incubated at RT for 5 min. 200 µl Chloroform was added and the tube mixed for 15 sec manually and spun at 15,000 g for 15 min at 4 ºC. The aqueous phase (~400 µL) was transferred to a fresh tube. RNA was purified using the RNA Clean & Concentrator-25 kit (Zymo Research) according to the manufacturer’s protocol, DNA digested by addition of 2 U of DNAase (Ambion) and incubation at 37 ºC for 30 min, followed by a further round of RNA purification as above.

RNA-seq libraries were prepared as stranded mRNA using TruSeq Stranded mRNA Library Preparation Kit (Illumina) according to the manufacturer’s instructions. Barcoded cDNA fragments of poly(A)+ RNA were then sequenced on a HiSeq 4000 instrument from Illumina with 2 × 76 + 7 bp paired end reads followed by a read demultiplexing step using Illuminas BCL to FASTQ file converter bcl2fastq version bcl2fastq v2.18.0.12. Demultiplexed reads were mapped against the mouse reference genome GRCm38 using HISAT 2.1.0. Alignment

files were sorted and indexed with SAMtools 1.6 [14]. Differential gene expression analysis was performed using the R package DESeq2 1.26.0 as described in the vignette [15]. Genes were identified to be differentially expressed if FDR < 0.05 for the comparison between A178D and WT. Gene set enrichment analysis (GSEA) was performed using the DEseq2 normalised read counts and the gene sets defined in the KEGG pathways database downloaded from MiSigDB (http://www.broadinstitute.org/gsea/msigdb/) [20].

The RNAseq data are available on Gene Expression Omnibus [https://www.ncbi.nlm.nih.gov/geo/] and can be accessed with GSE154504.

Initially 4 samples per genotype were processed, but one WT sample was excluded from analysis due to poor library complexity.

*Mass spectrometry*

Samples were prepared using a 96-well S-Trap plate (Protifi) following the manufacturer's protocol. Briefly, the samples were reduced and alkylated for 30 min each, acidified with 12 % phosphoric acid 10:1 (v/v) sample to acid, transferred onto the S-Trap resin and precipitated with 7 parts 90 % methanol in 100 mM TEAB to 1 part sample (v/v). Samples were washed 4 times by spinning the S-Strap plate at 1500 g for 30 sec, the last step for 1 min, each time with fresh 90 % methanol in 100 mM TEAB. The sample was resuspended in 50 µL 50 mM TEAB and digested with 1.5 µg trypsin (Promega) over night at 37 ºC in an incubator. Peptides were eluted from the S-Trap by spinning for 1 min at 1500 g with 80 µL 50 mM ammonium bicarbonate, 80 µL 0.1 % FA and finally 80 µL 50 % ACN/0.1 % FA. The eluate was dried in a vacuum centrifuge and resuspended in loading buffer (2 % ACN 0.1 % TFA) prior to mass spectrometry acquisition.

For LC-MS/MS data acquisition peptides were injected into a LC-MS system comprised of a Dionex Ultimate 3000 nano LC and a Thermo Q-Exactive. Peptides were separated on a 50-cm-long EasySpray column (ES803; Thermo Fisher) with a 75-µm inner diameter and a 60 minute gradient of 2 to 35 % acetonitrile in 0.1 % formic acid and 5 % DMSO at flow rate of 250 nL/min. MS1 spectra were acquired with a resolution of 70,000 and

AGC target of 3e6 ions for a maximum injection time of 100 ms. The 15 most abundant peaks were fragmented after isolation with a mass window of 1.6 Th at a resolution of 17,500. Normalized collision energy was 28 % (HCD).

Raw data was analysed using PEAKS software (v 8.5). Mass tolerances were set to 10 ppm (precursor) and 0.05 Da (fragment). We allowed up to 3 missed cleavage sites under tryptic digest restrictions and set Carbamidomethylation (Cys) as fixed modification while Oxidation (M) and Deamidation (Asn, Gln) were set as variable modifications. Data were searched against the reference proteome from *Mus musculus*, (retrieved 16/07/2018 from Uniprot). Peptide false discovery rate (FDR) was adjusted to 1 % before protein quantitation in PEAKS using the 3 most abundant peptides were applicable.

Top3 normalised values were loaded into Perseus version 1.6.6.0 [21] and subjected to log2 transformation and median subtraction. Student's t-test with permutation based FDR correction was used to identify peptides differentially represented between groups. A q-value of ≤ 0.05 was considered differentially expressed.

The mass spectrometry proteomics data have been deposited to the ProteomeXchange Consortium via the PRIDE [18] partner repository with the dataset identifier PXD020390 and 10.6019/PXD020390.

*Ex vivo* studies

Tibial length measurements, mRNA isolation from ventricular tissue, reverse transcriptase and quantitative PCR (qPCR) were performed as described [6] with the Taqman probes (Applied Biosystems) listed in Table S11. Phostag-assays were performed as described [13].

To confirm the presence of the titin A178D variant at transcript level, the relevant portion of *Ttn* was amplified by PCR from cDNA of a HET heart using the following primers: 5'- GTAAAACGACGGCCAGTCAGAGCTCCCTTGATTTCCA-3' and 5'-CAGGAAACAGCTATGACCAATCCGGGTTTGTCTTGTT-3' and the product subjected to Sanger sequencing. cDNA where no reverse transcriptase had been added served as control.

*Titin SDS-PAGE*

Analysis of titin by SDS-PAGE was performed as described [19]. To confirm the presence of the variant at protein level, the titin band of a protein sample of an A178D heart was subjected to in gel tryptic digest and analysis by mass spectrometry was performed (see below).

Hearts were homogenized in sample buffer containing 8 M urea, 2 M thiourea, 3% SDS, 75 mM DTT, 10% glycerol, bromophenol blue and 0.05 M Tris· HCl, pH 6.8 (samples prepared as described in [16]). Samples were incubated for 5 min on ice and boiled for 5 min at 95°C, followed by centrifugation. Agarose-strengthened SDS-PAGE (2 % polyacrylamide; 0.5 % agarose) was performed, at 2 mA overnight, and protein bands were visualized by Coomassie Brilliant Blue staining. ProQDiamond stain (Thermofisher) to visualise phosphorylated titin was used according to the manufacturer's instructions.

*Protein fractionation*

Cardiac tissue powder was lysed in ice-cold lysis buffer (pH 7.5) containing 50 mM Tris-HCl, 5 mM EGTA, 2 mM EDTA, 5 mM DTT as well as 0.05% digitonin and protease inhibitor mixture (Roche Applied Science), using 100 µL per 10 mg of tissue. Lysates were then centrifuged at 14,000 g for 30 min at 4 °C, and the supernatant, which comprised the cytosolic fraction, was removed. The pellet was then solubilized in an equal volume of the digitonin-based lysis buffer containing 1% Triton X-100 and centrifuged at 14,000 g for 30 min at 4 °C, and the supernatant, which comprised the membrane fraction, was removed. The remaining Triton-insoluble pellet contained the myofilament fraction, which was used for proteomics analysis. Equal volumes of sample buffer (8 M urea, 2 M thiourea, 3% SDS, 75 mM DTT, 10% glycerol, bromophenol blue and 0.05 M Tris· HCl, pH 6.8) were added to cytosolic and myofilament fractions prior to Western blot analysis.

*Cardiomyocyte isolation*

12 mice of each genotype were heparinized (100 U) 10 min before being euthanized, hearts were rapidly excised, the aorta cannulated and perfused on a Langendorff system at 37 °C

with tyrode solution (130 mM NaCl, 5.6 mM KCl, 3.5mM $MgCl_2$), 5 mM 4-(2-hydroxyethyl)-1-piperazineethanesulphonic acid, HEPES, 0.4 mM $Na_2HPO_4$) for 3 minutes. Hearts were then perfused with enzyme solution (tyrode solution with 100 µM $CaCl_2$ and 2.5 mg/ml liberase TH, Roche). The left ventricle was dissected, cut into small pieces and dissociated by tituration at 37 °C to obtain a cell suspension which was passed through a 250 µm mesh, diluted with 3 volumes of 1% bovine serum albumin (BSA) in tyrode solution and centrifuged at 100 g for 3 minutes. Cell pellets were re-introduced to $Ca^{2+}$ in a step-wise fashion (8 mins in 1% BSA containing 500 µM $Ca^{2+}$ then 8 mins in 1% BSA containing 1 mM $Ca^{2+}$) and then resuspended in storage buffer (120 mM NaCl, 5.6 mM KCl, 5 mM $MgSO_4$, 5 mM sodium pyruvate, 10 mM HEPES, 200 mM glucose, 200 mM taurine, 0.5 mM $Ca^{2+}$) for further use.

*Cardiomyocyte contractility and calcium transients*

For measurements on the IonOptix µstep apparatus, isolated cardiomyocytes were allowed to settle on a 1.5 mm coverslip in the perfusion chamber for 5 minutes before being perfused with 37 °C tyrode solution supplemented with 1.4 mM $Ca^{2+}$ and electrically paced with 40 volts at a frequency of 1 Hz. Cardiomyocyte contraction was assessed using phase-contrast microscopy. Cells with poor morphology (e.g. excessive blebbing or asynchronous contraction) were not measured. Cells with basal sarcomere lengths or contraction times more than two standard deviations from the mean were excluded as an artefact of the deteriorating function of primary cells with time in culture.

Ratiometric measurement of intracellular calcium ($[Ca^{2+}]_i$) transients were done in fura-2 loaded LV cardiomyocytes isolated from the hearts of WT or TG mice. After isolation, cardiomyocytes were loaded for 8 mins with Fura2-AM (3 µM) in a low-$Ca^{2+}$ Tyrode solution (500 µM $Ca^{2+}$ + Pluronic) followed by two washes in low- $Ca^{2+}$ Tyrode to remove any unloaded Fura2-AM dye. Loaded cardiomyocytes were then placed in a glass-bottom chamber, mounted on to an inverted microscope, and perfused at 35 ± 1°C with 1.4 mM $Ca^{2+}$ Tyrode solution. Cardiomyocytes were field-stimulated at 3 Hz for several minutes to allow steady-state $[Ca^{2+}]_i$ transients to be reached before recordings were taken. For each set of recordings,

background fluorescence was taken from a cell-free field. $[Ca^{2+}]_i$ transient were measured as a function of the ratio of emissions from 340/380 excitation wavelength after subtraction of the background fluorescence (IonOptix). The baseline (i.e. diastolic) and peak $[Ca^{2+}]_i$ transient was determined from an average of at least 10 individual tracings, with the difference between the peak and diastolic $[Ca^{2+}]_i$ transient used to quantify the amplitude. The time constant (tau, τ) of the $[Ca^{2+}]_i$ decay was evaluated using a biexponential fit (Axon Clampfit).

*Cardiomyocyte size and immunofluorescence*

Isolated cardiomyocytes were deposited on poly-D-lysine coated slides using a StatSpin cytofuge at 700 rpm for 2 min, then fixed for 10 minutes with 4 % paraformaldehyde in PBS. 100x magnification bright-field images of 27-39 rod-shaped cardiomyocytes per mouse were obtained. ImageJ was used to measure the area, maximum width and maximum length of each cardiomyocyte. Immunofluorescence on fixed adult mouse cardiomyocytes on poly-D-lysine coated slides (see above) was performed as described [3, 10] using the primary antibodies indicated in Table S11.

For T-tubular visualisation, 1 µM of di-4-ANEPPS and 2 µM Pluronic® F-127 (Life Technologies) were directly added to freshly isolated cardiomyocytes for 20 minutes, cells were washed with storage buffer. They could then be visualised immediately using a Leica TCS SP5 X confocal microscope equipped with a 433 nm argon laser and a 63X oil lens. Images were acquired using a photomultiplier set between 500-600 nm for optimum image resolution and fluorescence intensity.

*High Resolution Episcopic Microscopy (HREM)*

Adult hearts were collected and flushed with PBS to remove blood. Hearts were fixed overnight in 4% paraformaldehyde in PBS prior to extensive washing with PBS. A methanol series was performed (10%, 20%, 30%, 40%, 50%, 60%, 70%, 80%, 90%, 95%, 100%, 100%) for 2 hours each prior to infiltration overnight in a 50:50 mixture of 100% methanol and JB-4 resin (18570-1, Polysciences), including 0.275g/100ml eosinB (Sigma Aldrich) and 0.055g/100ml acridine

orange (Sigma Aldrich). Hearts were washed in the JB-4 infiltration solution for 1 hour prior to incubation for 1 week in fresh JB-4 solution. Hearts were sectioned at 3 µm using an optical HREM microscope (Indigo Scientific). Sections were reconstructed in 3D using Horos DICOM viewer (The Horos Project) and Amira (ThermoFisher Scientific) software. Horos was used to calculate ventricular length and width, expressed as a ratio of length:width. Average myocardial wall thickness was calculated from 5 separate measurements for each heart along the ventricular wall. To assess the fractal dimensions of left ventricle trabeculae, 20 evenly spaced sections from base to apex of the heart were assessed using the LV Fractals Analysis plugin for Osirix and Horos [2].

*Electron microscopy*

Specimens for electron microscopy were prepared from frozen samples which were brought to 0 °C in a buffer containing PBS, 1 mM $MgCl_2$, 5 mM EGTA and 20 mM 2,4, butanedione monoxime (BDM). The pieces were then fixed in 2.5 % glutaraldehyde, 2 % PFA in the same buffer for 90 min on ice. After washing, small pieces were subsequently fixed in 1 % osmium tetroxide, dehydrated in alcohol and embedded in Araldite. Thin sections were stained with UranyLess stain followed by lead citrate. Sections were viewed in a JEM 1400 transmission electron microscope in the Centre for Ultrastructure Imaging, King's College London.

For assessment of Z-disc width, data was obtained from scans of Z-discs in Image J (v.1.53c) where it was judged there was little or no overlap of the ends of the thick filaments with the Z-disc. Width limits were taken as positions at half the density difference between the central Z-disc and the neighbouring I-band.

*Passive tension measurements*

Left ventricular endocardial septal trabeculae were dissected in dissecting solution and demembranated over night at 4 °C in skinning solution. Before the experiment, preparations were further thinned out, and aluminium "T-clips" were attached to each end. The Aurora Scientific (Aurora, ON, Canada) 1400A permeabilised fibre apparatus, with 403A force

transducer and 315C-I length controller, was used for tension mesurements and length control. After mounting, the fibre the baseline sarcomere length (SL) was set at 2.0 µm (1.0 L0) using laser diffraction, and allowed to stabilise for ten minutes in relaxing solution. The preparation length and thickness in two dimension was measured. The latter were used to approximate cross sectional area to an elliptical form. A series of four incremental stretches of 50 ms duration, increasing the fibre length on 5 % increments, length clamped for 1 s at each increment, to a maximum of 1.2 $L_0$ was performed followed by a return to SL 2.0. Incubation in relaxing solution supplemented with 0.9 M KCl and then relaxing solution with 0.6 M KI as described elsewhere [12] removed the titin derived passive tension after which the stretch procedure was repeated. To derive titin generated tension, post-treatment values were subtracted from pre-treatment values. All tension values were normalised to cross sectional area (mN/mm2) and titin fraction of total tissue derived by dividing titin derived tension by total tension. All experiments were carried out at 15 °C.

Solutions used: Relaxing solution: EGTA (10 mM), KCl (100 mM), $MgCl_2$ (5.6 mM), $Na_2ATP$ (5 mM), phosphocreatine (10mM), imidazol (20 mM), EDTA free protease inhibitor cocktail (Roche Diagnostics). Dissecting solution: Relaxing solution supplemented with 20 mM BDM and NaF (1mM), Skinning solution: Dissecting solution supplemented with 1 % v/v Triton X-100. All solutions were set to pH 7.0 at 15 °C using KOH. All chemicals purchased from Sigma Aldrich unless otherwise stated.

*Immunofluorescence on cryo-sections*

Cryo-section were done on O.C.T. (VWR) embedded tissue on a ryotome Cryotome FSE (Thermo Scientific; 10 µm thickness). Cryo-sections from *tibialis anterior* (cross-sections) were performed as described [1], using laminin to visualise the extracellular matrix [11]. Cryo-sections on cardiac tissue were performed as above and unfixed sections stained and imaged as described for isolated cardiomyocytes. Antibodies and their dilutions are given in Table S11.

*TUBE-assay*

Lysis of frozen tissue was performed as described [7] using following buffer 1% Triton X-100, 20 mM Tris-HCl (pH 7.6), 138 mM sodium chloride, 5 mM dithiothreitol, 5 % glycerol, phosphatase and protease inhibitors (Roche), 100 mM N-ethylmaleimide (Sigma) and 50 µM PR-619 (LifeSensors via Tebu-Bio). Lysates (250 ug protein) were incubated with 20 µL TUBE1 agarose (LifeSensors via Tebu-Bio) in 500 µL buffer overnight at 4 °C (shaking). As control, the same agarose matrix without TUBEs was used (LifeSensors via Tebu-Bio). Next day, the beads were washed 3 times with 1 mL buffer, bound proteins were eluted in 30 µL of SDS sample buffer and subsequently heated (100 °C 3 min) and analysed by Western blotting.

*Histology*

Histology on paraffin embedded samples (7 µm sections) was performed with hematoxylin and eosin or with Sirius red, using standard protocols.

**Supplementary Figures:**

**Figure S1:** Generation of the mouse model harbouring the *Ttn* A178D variant. A – Scheme of position of genotyping primers, the A178D variant and the introduced DdeI restriction site. DdeI digest of PCR product from the WT allele results in a 401 bp fragment (black arrow in B), while the *Ttn* A178D allele is digested into a 245 bp and a 155 bp fragment (white arrows in B). B – Examples of digested PCR products used for genotyping. The positions of marker bands are indicated. Ctr – PCR control without genomic DNA. C Left – reverse transcriptase PCR was performed on mRNA isolated from WT and HET hearts (n = 2, age 45/40 d, all females), a PCR product is only obtained in the presence of reverse transcriptase (RT) enzyme. The positions of marker bands are indicated. Right – Sanger sequencing trace of this PCR product shows the presence of both WT and A178D allele (arrow). D – Identification of titin A178D protein in a homozygous heart (417 d, male) by mass spectrometry: peptides containing A178D (arrow) were identified based on their molecular weight.

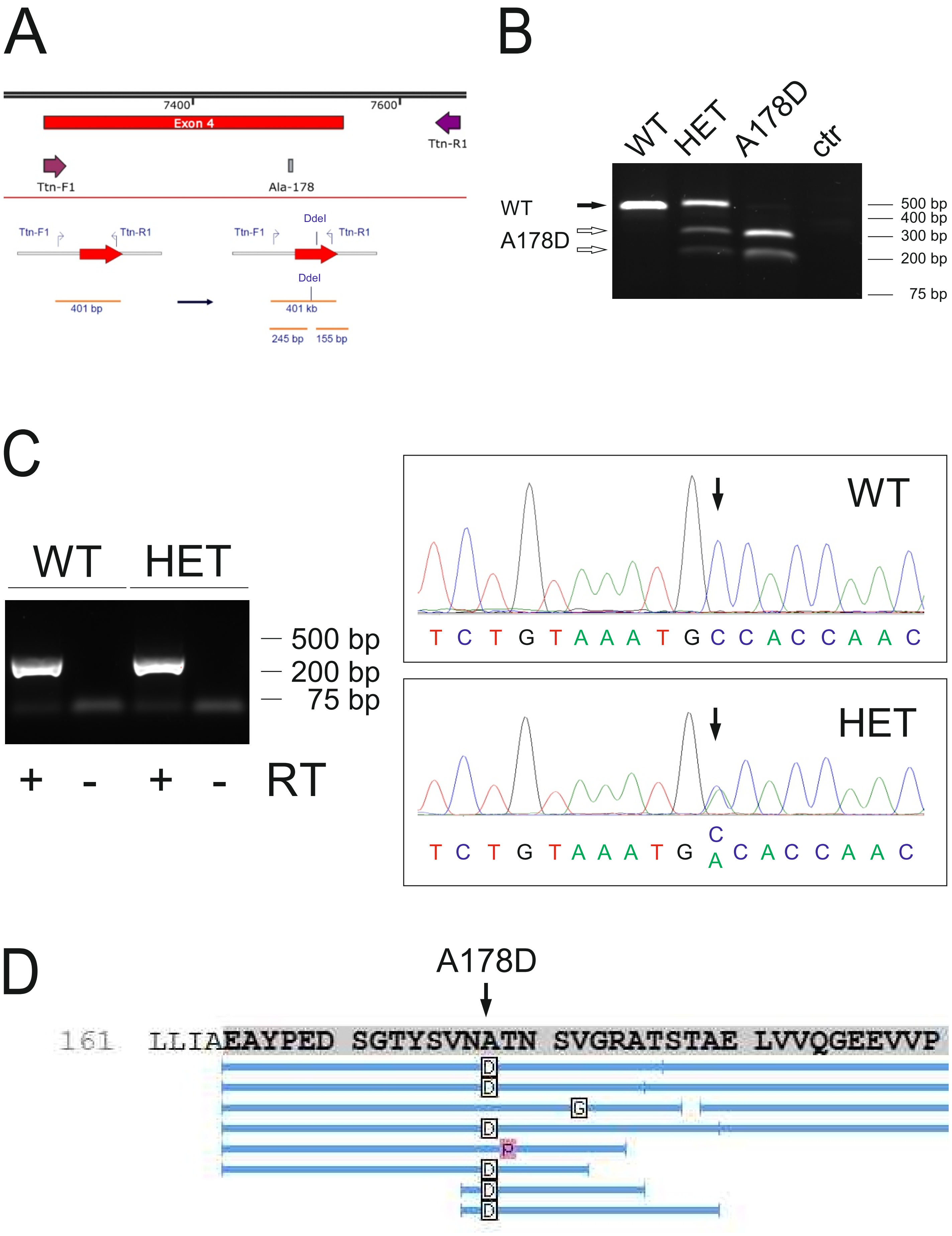
A
7400
7600
Exon 4
Ttn-R1
Ttn-F1
Ala-178
Ttn-F1
Ttn-R1
DdeI
Ttn-F1
Ttn-R1
DdeI
401 bp
401 kb
245 bp
155 bp
B
WT
HET
A178D
ctr
WT
A178D
500 bp
400 bp
300 bp
200 bp
75 bp
C
WT
HET
500 bp
200 bp
75 bp
+ - + - RT
WT
T C T G T A A A T G C C A C C A A C
HET
T C T G T A A A T G C/A C A C C A A C
D
A178D
161 LLIAEAYPED SGTYSVNATN SVGRATSTAE LVVQGEEVVP
D
D
G
D
P
D
D
D



Figure S1

**Figure S2:** A - *Ttn* transcript is normally expressed in young (left) and aged (right) titin A178D mice as assessed by qPCR. (Scatterplots with mean ± SEM, left: WT: n = 10 (4M/6F), age 114.2 ± 0.7 d; A178D n = 12 (6M/6F), age 113.4 ± 0.5 d; Mann-Whitney U-test, right: WT: n = 5, age 411 ± 3 d, HET n = 6, age 412 ± 2 d, A178D n = 6, age 423 ± 3 d, all male, Kruskal-Wallis followed by Dunn’s multiple comparison’s. B – Normal titin expression, isoform composition and phosphorylation in young A178D hearts. titin isoforms N2BA and N2B were resolved by agarose-SDS-PAGE and stained with Coomassie (top). The position of the T2 band is also indicated. ProQDiamond stain (middle) failed to detect changes in global titin phosphorylation. (Please note, the bottom right corner of the gel cracked off.) A conventional Coomassie stained SDS-PAGE (10 % PAA) served as loading control (bottom). WT/A178D n = 4, age 95 d, all males.

C – Same analysis (without ProQDiamond stain) as in B for aged HET and homozygous titin A178D mice. No changes in titin expression or isoform composition were observed. WT/HET/A178D n = 4, age 410 ± 2 d/413 ± 2 d/424 ± 5 d, all males.

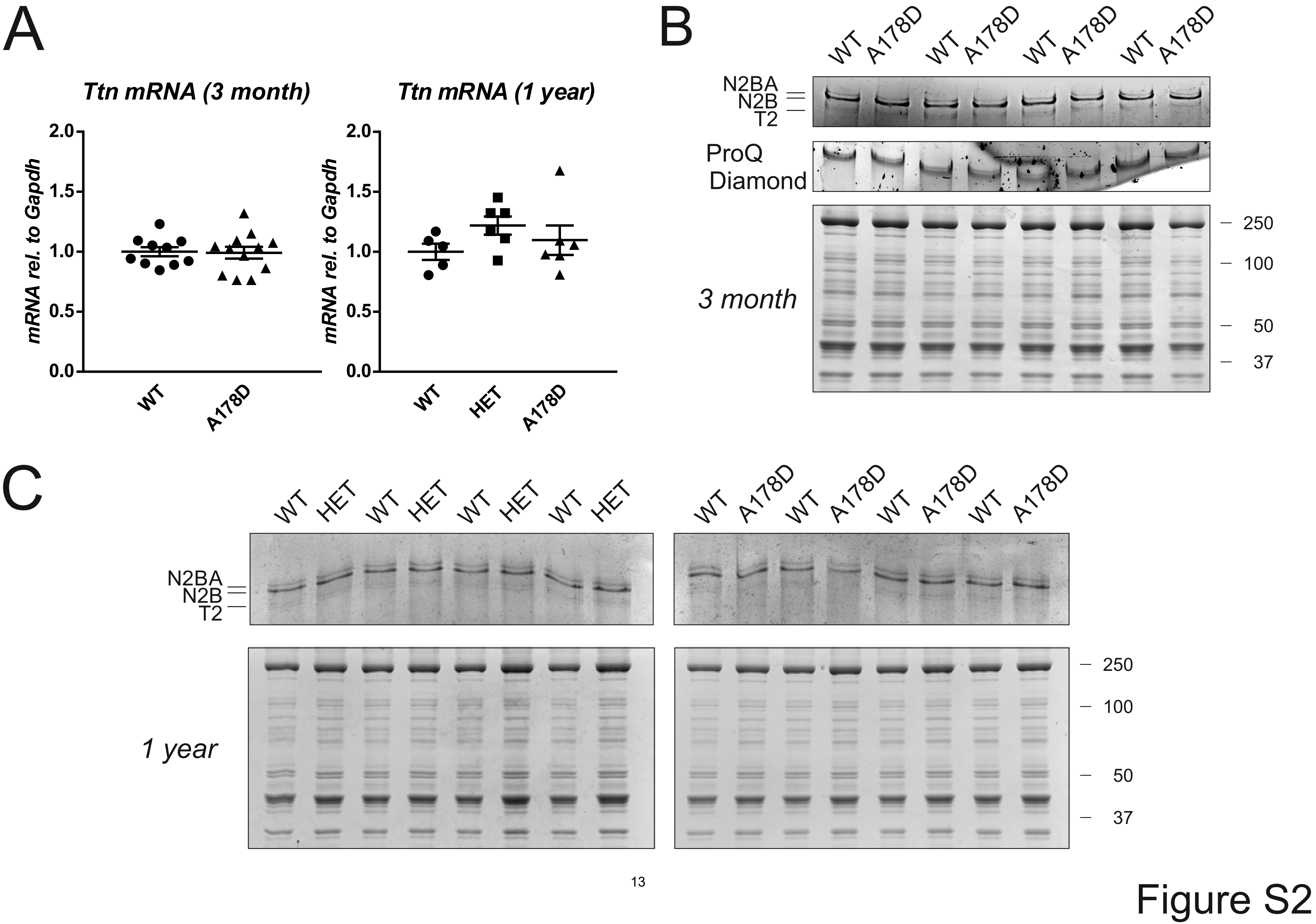
A
Ttn mRNA (3 month)
Ttn mRNA (1 year)
mRNA rel. to Gapdh
2.0
1.5
1.0
0.5
0.0
WT
A178D
HET
B
WT
A178D
N2BA
N2B
T2
ProQ
Diamond
3 month
250
100
50
37
C
1 year
Figure S2

**Figure S3:** Quantification of titin blots from Figure S2. A – Amount of titin (measured as N2BA+N2B+T2) relative to the sarcomeric protein myosin heavy chain. B – Amount of titin T1 = N2BA+N2B relative to T2. Changes in this ration can be indicative of altered turnover. C – Titin phosphorylation assessed by ProQDiamond stain from Fig. S2B (N2BA+N2B bands) normalised to Coomassie stain of the same titin bands.

No significant changes were observed (Mann-Whitney U-test, n = 4). 3 month: gels from Fig. S2B, 1 year HET gels from Fig. S2C left, 1 year A178D gels from Fig. S2C right.

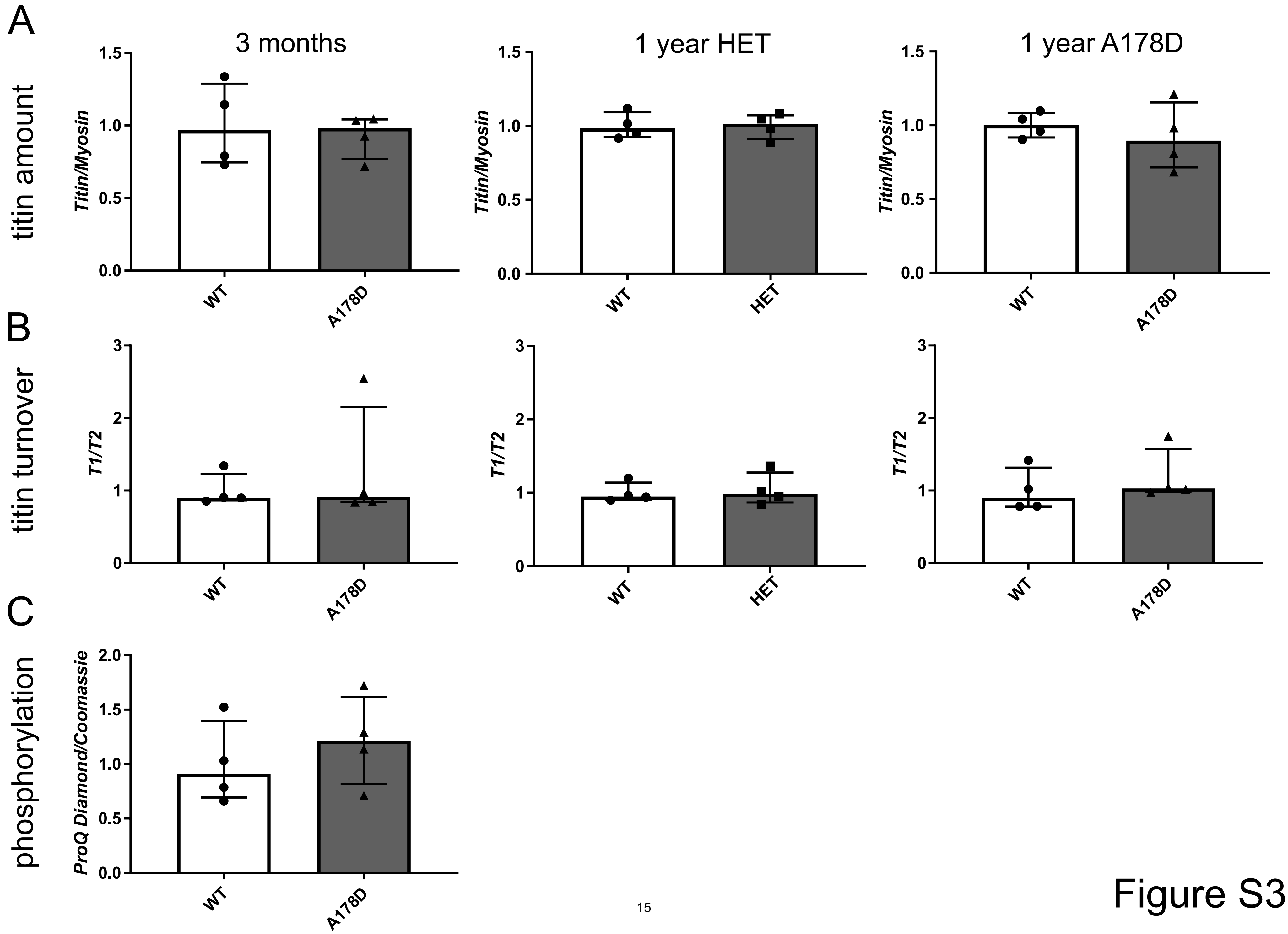
A
B
C
3 months
1 year HET
1 year A178D
titin amount
titin turnover
phosphorylation
Titin/Myosin
T1/T2
ProQ Diamond/Coomassie
WT
A178D
HET
0.0
0.5
1.0
1.5
2.0
0
1
2
3

Figure S3

**Figure S4:** Normal organisation of titin epitopes in cardiomyocytes isolated from A178D hearts: Isolated cardiomyocytes from WT and A178D mice were stained with following antibody combinations: first panel - titin Z1Z2 epitope and Z-disc marker alpha-actinin; merged images: Z1Z2 green, alpha-actinin red; second panel: titin M-band epitope m8 and M-band marker myomesin; merged images: m8 green, myomesin red; third panel – titin M-band epitope m8 and a titin epitope near the Z-disc (T12); merged images: m8 green, T12 red; scale bars represent 5 µm. WT: female, age 118 d, A178D male, age 168 d.

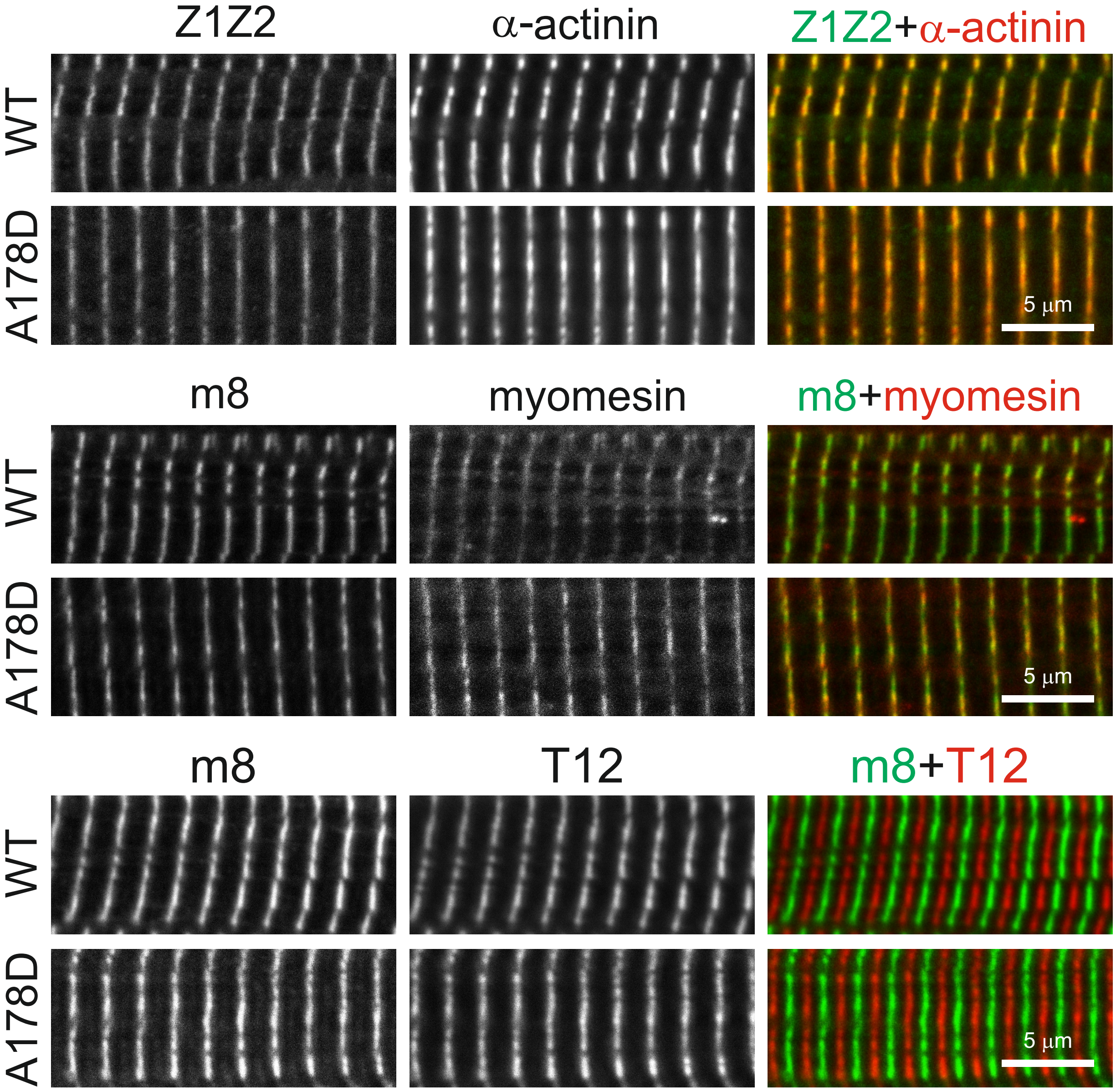
Z1Z2
α-actinin
Z1Z2+α-actinin
WT
A178D
5 μm
m8
myomesin
m8+myomesin
WT
A178D
5 μm
m8
T12
m8+T12
WT
A178D
5 μm



Figure S4

**Figure S5:** A – Normal ultrastructure of A178D hearts compared to WT hearts. Electron microscopy images from frozen tissue. Scale bar represents 1 micron, Z-discs are indicated by white arrows. B – Histogram of Z-disc width of WT and A178D samples, average 90 ± 3 nm and 93 ± 4 nm, respectively. (WT n = 33, A178D n = 19 from two mice each; age 112 d/115 d WT/A178D; all males).

A

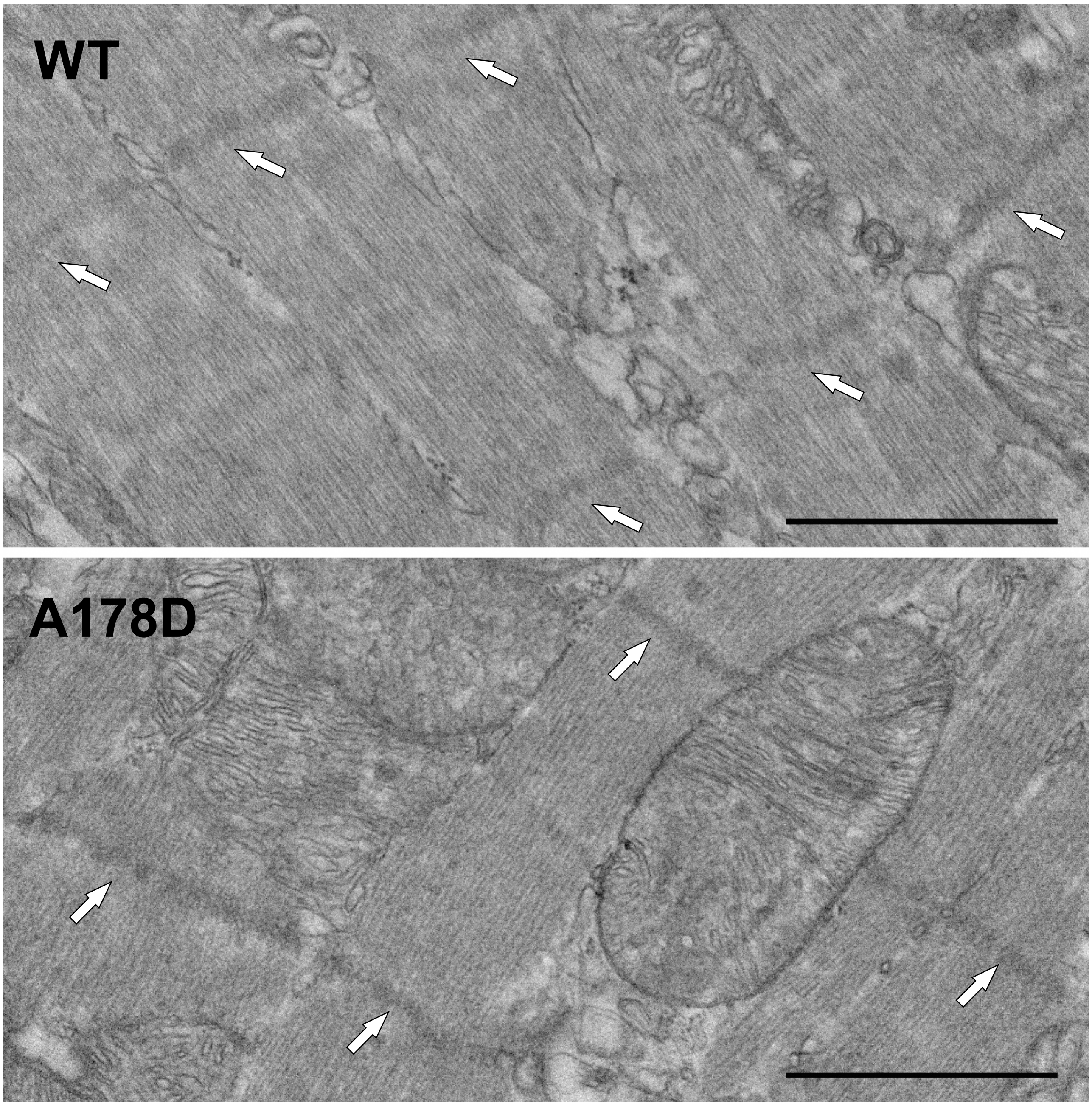

WT
A178D


B

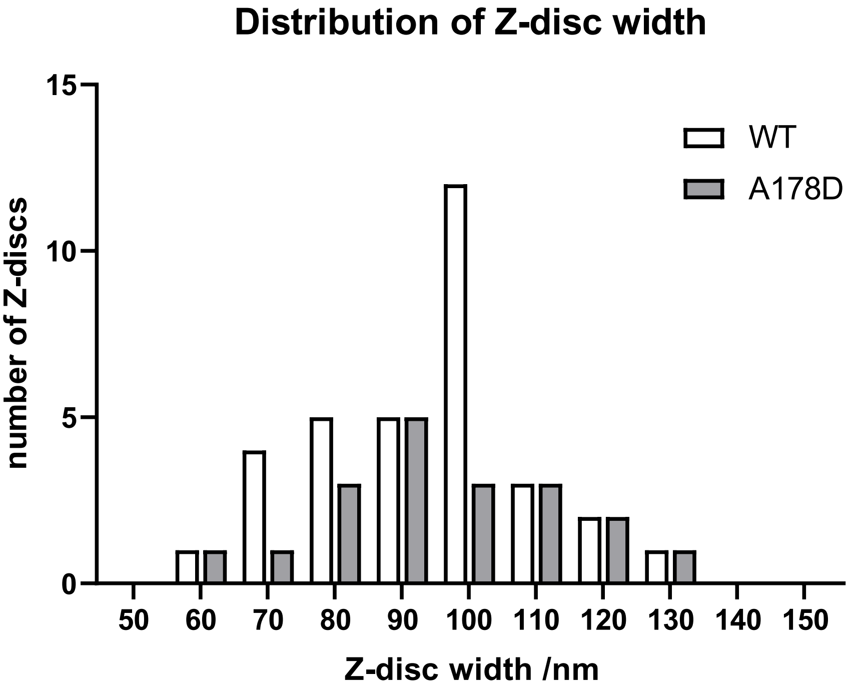

Distribution of Z-disc width
number of Z-discs
0
5
10
15
WT
A178D
50
60
70
80
90
100
110
120
130
140
150
Z-disc width /nm


Figure S5

**Figure S6:** Mice heterozygous for the *Ttn* A178D (HET) do not have an overt cardiac phenotype. All experiments were performed on male mice. A – Echocardiographic measurements: fractional shortening and end-diastolic dimensions are normal at all time points investigated. For cohort characteristics and a wider set of echocardiographic parameters please refer to Table S1 (Mann-Whitney U-test at each age; n = 9 WT, n = 10/9/8 A178D 3 months (96-97 d)/6 months (183-184 d)/1 year (385-386 d)). B – Heart weight normalised to tibial length is normal in 1 year old HET hearts (n = 9/8 WT/HET; Student's t-test, 385-386 d). C – Invasive haemodynamic assessment of LV performance indicates normal systolic and diastolic function in 1 year old HETs: $dP/dt_{max}$, $dP/dt_{min}$ and relaxation constant Tau are shown at baseline conditions, and under adrenergic stimulation (dobutamine infusion at 4 ng $g^{-1}$ BW $min^{-1}$ and 16 ng $g^{-1}$ BW $min^{-1}$). For cohort characteristics and a wider set of parameters refer to Table S2 (n= 8/7 WT/HET; 2-Way-ANOVA, age 424-425 d).

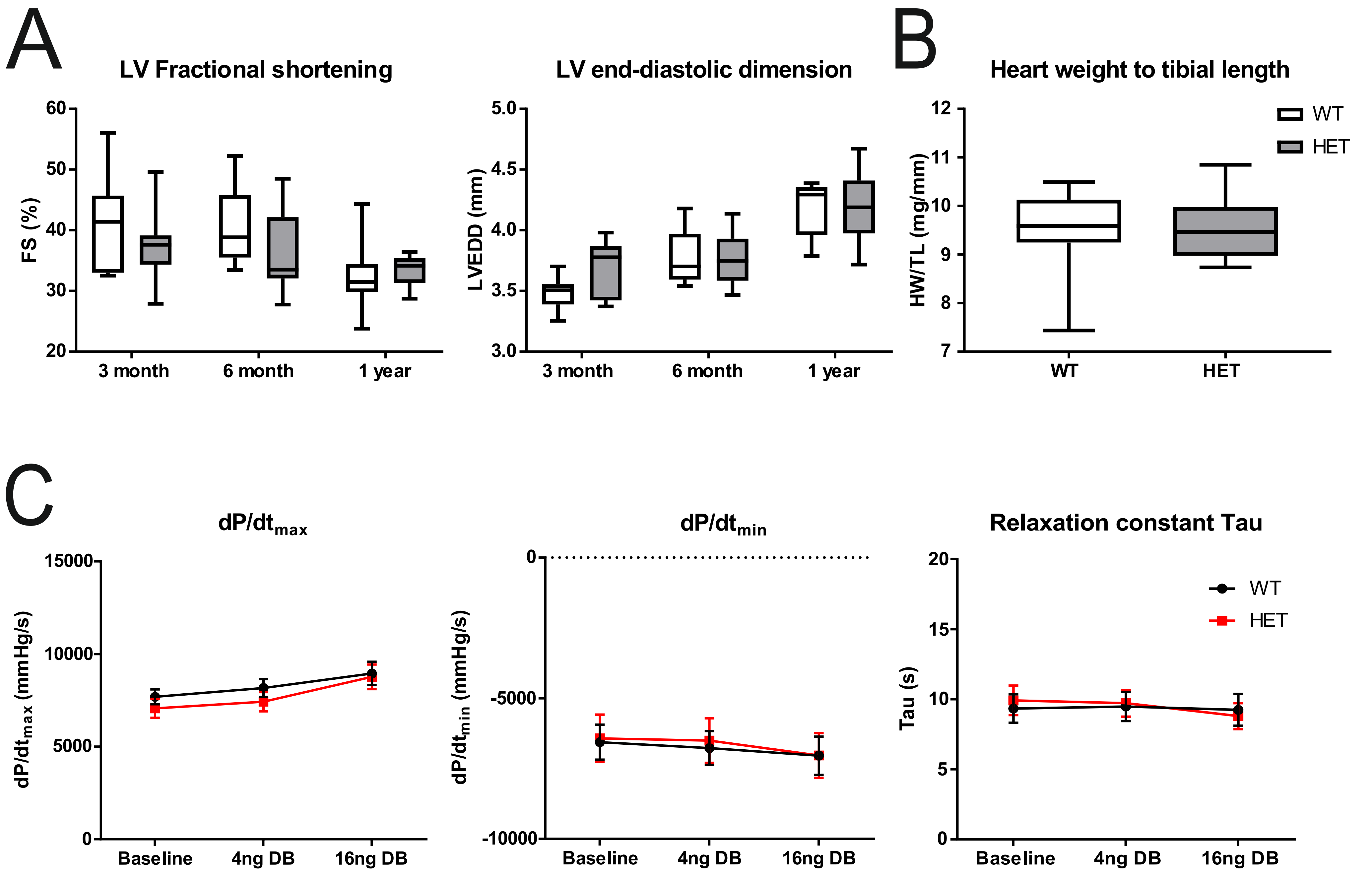
A
LV Fractional shortening
FS (%)
60
50
40
30
20
3 month
6 month
1 year
LV end-diastolic dimension
LVEDD (mm)
5.0
4.5
4.0
3.5
3.0
3 month
6 month
1 year
B
Heart weight to tibial length
HW/TL (mg/mm)
12
11
10
9
8
7
WT
HET
WT
HET
C
dP/dtmax
dP/dtmax (mmHg/s)
15000
10000
5000
0
Baseline
4ng DB
16ng DB
dP/dtmin
dP/dtmin (mmHg/s)
0
-5000
-10000
Baseline
4ng DB
16ng DB
Relaxation constant Tau
Tau (s)
20
15
10
5
0
WT
HET
Baseline
4ng DB
16ng DB



Figure S6

**Figure S7:** HREM 3D reconstructions of WT and A178D hearts. (A-D) Wild-type. (E-H) A178D mutant. (A, B, E, F) Volume rendered representations of right ventricle (blue) and left ventricle (red) luminal surfaces. (C, D, G, H) Greyscale images show 3D reconstructions of hearts. (A, E) Ventral and (B, F) dorsal views are shown. (C, G) Dorsal cross-sections through heart and (D, H) sagittal cross-sections through right ventricle are shown. (D, H) The interior aspect of the left ventricle is also shown, perpendicular to the septum. (I, J) Volumes of left and right ventricle lumens. (K, L) Total volumes of left and right ventricles including the myocardial portion. (M) Length:width ratio. (N) Myocardial wall thickness. (O) Fractal dimensions of left ventricle trabeculae. Data was tested for statistical significance (set as $p < 0.05$) by Student's t-test (M, N), Mann Whitney U-test (I, J, K, L), or one-way ANOVA with Tukey's post-hoc test (O). n = 5 per group. WT – black bars or circles, A178D – grey bars or red triangles. All data is expressed as mean ± SEM. Age: WT 116 ± 1 d (all male), A178D 115 ± 1 d (1 female, 4 males).

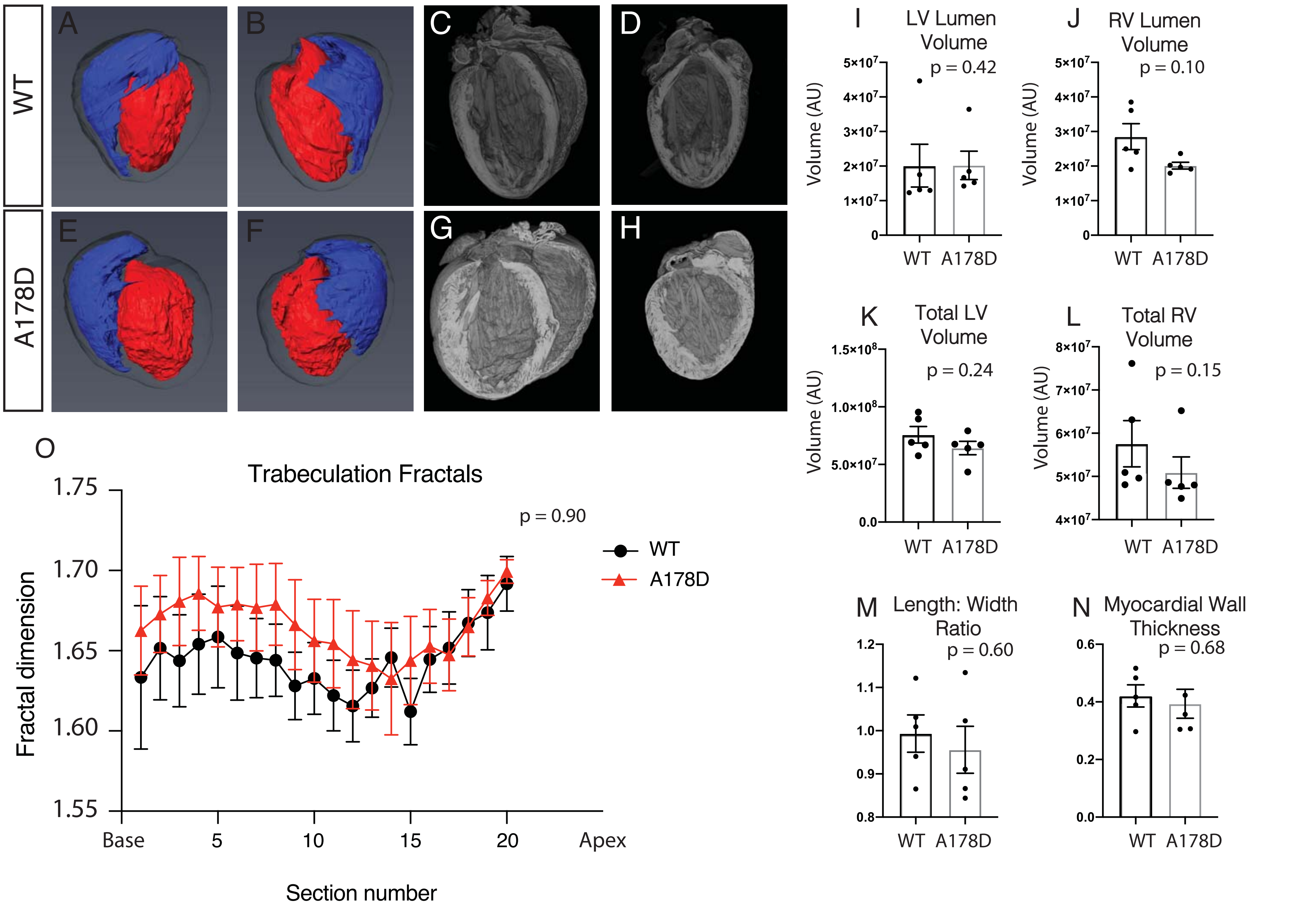

WT
A178D
A
B
C
D
E
F
G
H
I
LV Lumen Volume
p = 0.42
Volume (AU)
5×10^7
4×10^7
3×10^7
2×10^7
1×10^7
0
WT A178D
J
RV Lumen Volume
p = 0.10
Volume (AU)
WT A178D
K
Total LV Volume
p = 0.24
Volume (AU)
1.5×10^8
1.0×10^8
5.0×10^7
0.0
WT A178D
L
Total RV Volume
p = 0.15
Volume (AU)
8×10^7
7×10^7
6×10^7
5×10^7
4×10^7
WT A178D
M
Length: Width Ratio
p = 0.60
1.2
1.1
1.0
0.9
0.8
WT A178D
N
Myocardial Wall Thickness
p = 0.68
0.6
0.4
0.2
0.0
WT A178D
O
Trabeculation Fractals
p = 0.90
Fractal dimension
1.75
1.70
1.65
1.60
1.55
Base
5
10
15
20
Apex
Section number
WT
A178D




Figure S7

**Figure S8:** No skeletal muscle abnormalities in A178D mice: cross-sections of *tibialis anterior* stained for laminin (green) and nuclei (blue). Fibre size appears normal and there is no evidence of increased central nuclei localisation in A178D skeletal muscle fibres, which would be indicative of muscle regeneration. There is no suggestion of increased fibrosis, either. Scale bar represents 50 microns. All animals in the experiment are male; n = 3, age 100 d WT, 96 d A178D.

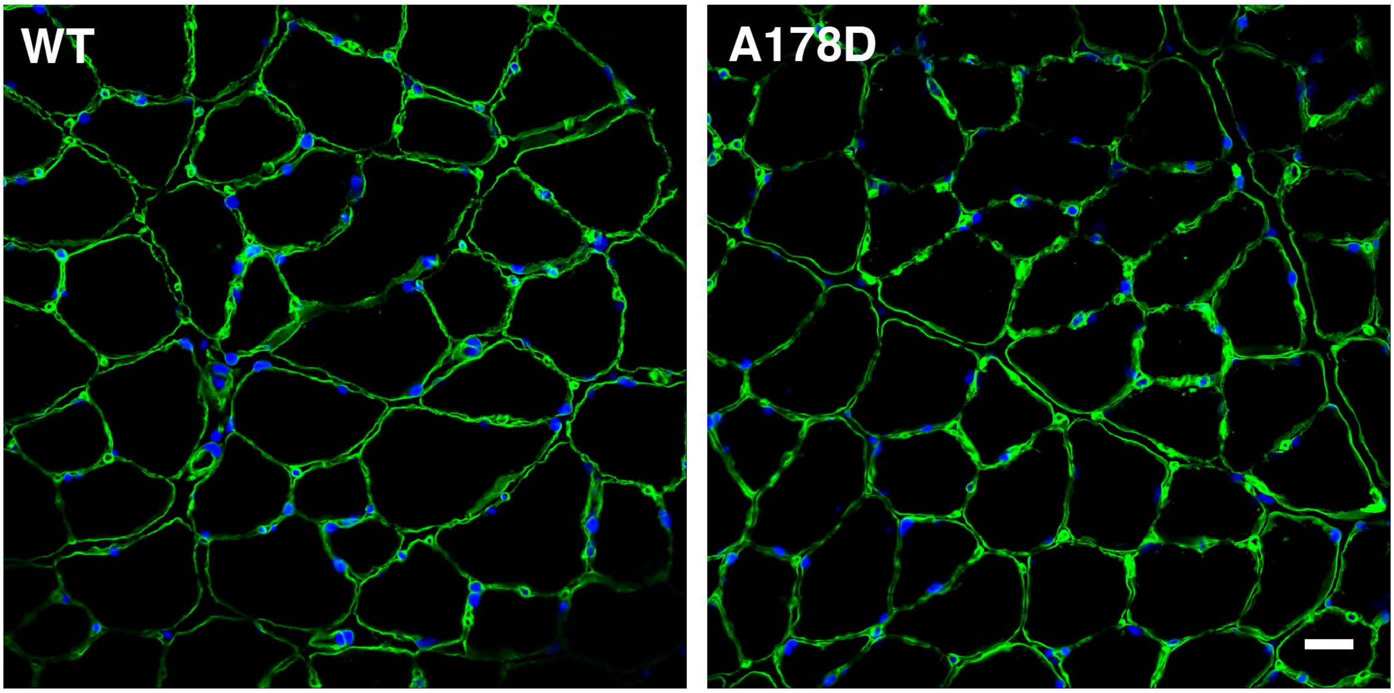




Figure S8

**Figure S9:** Cardiac phenotype of aged A178D mice. Echocardiographic measurements: fractional shortening, end-systolic and end-diastolic dimensions are shown (Student’s test, n = 13/10, age 385±4 d/391 ± 2 d WT/A178D). For cohort characteristics and a wider set of echocardiographic parameters please refer to Table S4. Heart weight normalised to tibial length on the same animals is also shown (Student’s test). All animals in this experiment were male.

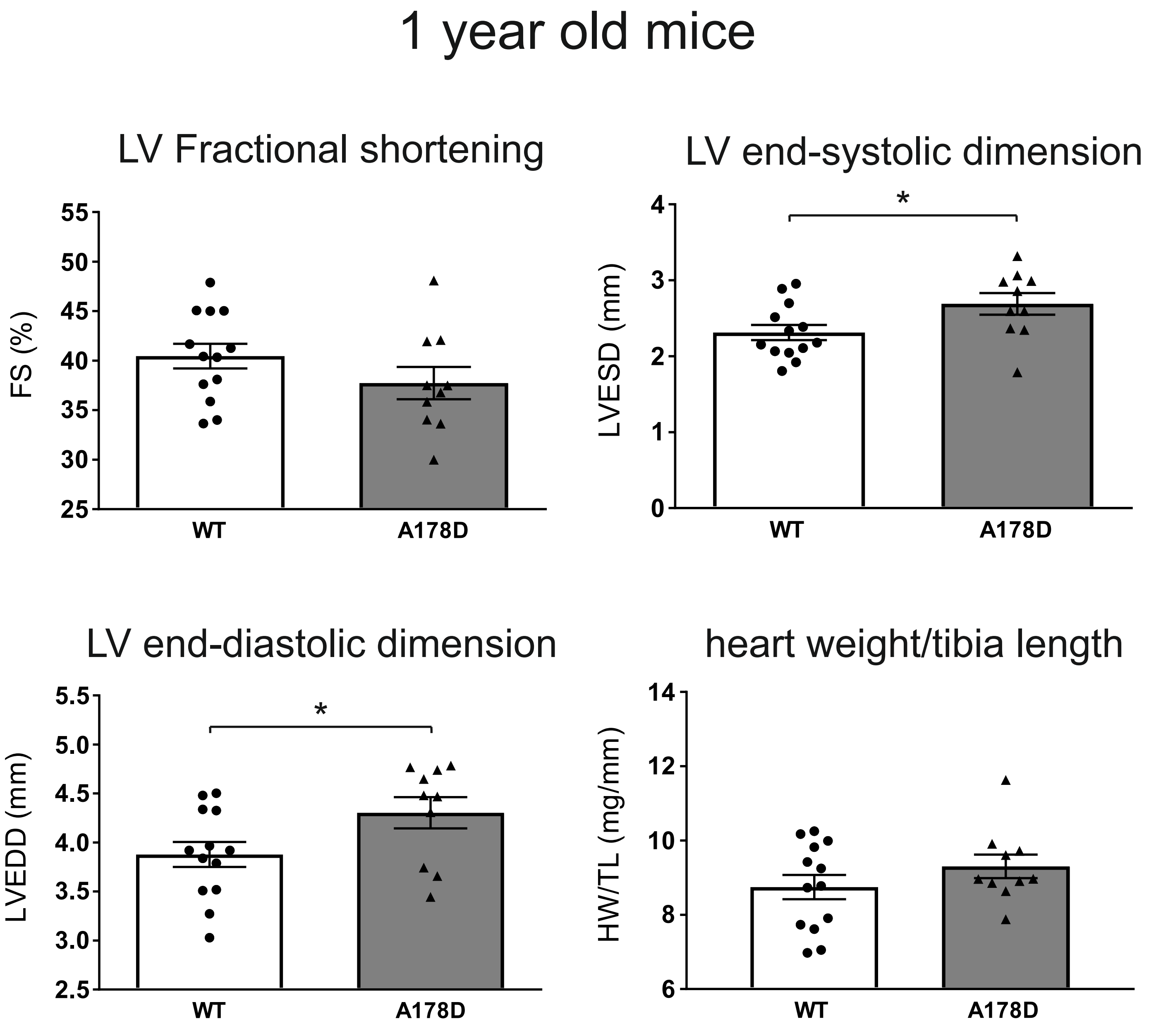
1 year old mice
LV Fractional shortening
FS (%)
55
50
45
40
35
30
25
WT
A178D
LV end-systolic dimension
LVESD (mm)
4
3
2
1
0
*
WT
A178D
LV end-diastolic dimension
LVEDD (mm)
5.5
5.0
4.5
4.0
3.5
3.0
2.5
*
WT
A178D
heart weight/tibia length
HW/TL (mg/mm)
14
12
10
8
6
WT
A178D

Figure S9

**Figure S10:** Frequency distribution (medium smoothing) of cell area (top), cell length (middle) and cell width (bottom) distribution for each individual cell isolation (see Figure 2) shown by violin plots displaying median and quartiles. (WT: isolations from 12 hearts (4F/8M), age 128 ± 8 d; A178D isolations from 12 hearts (4F/8M), age 132 ± 10 d).

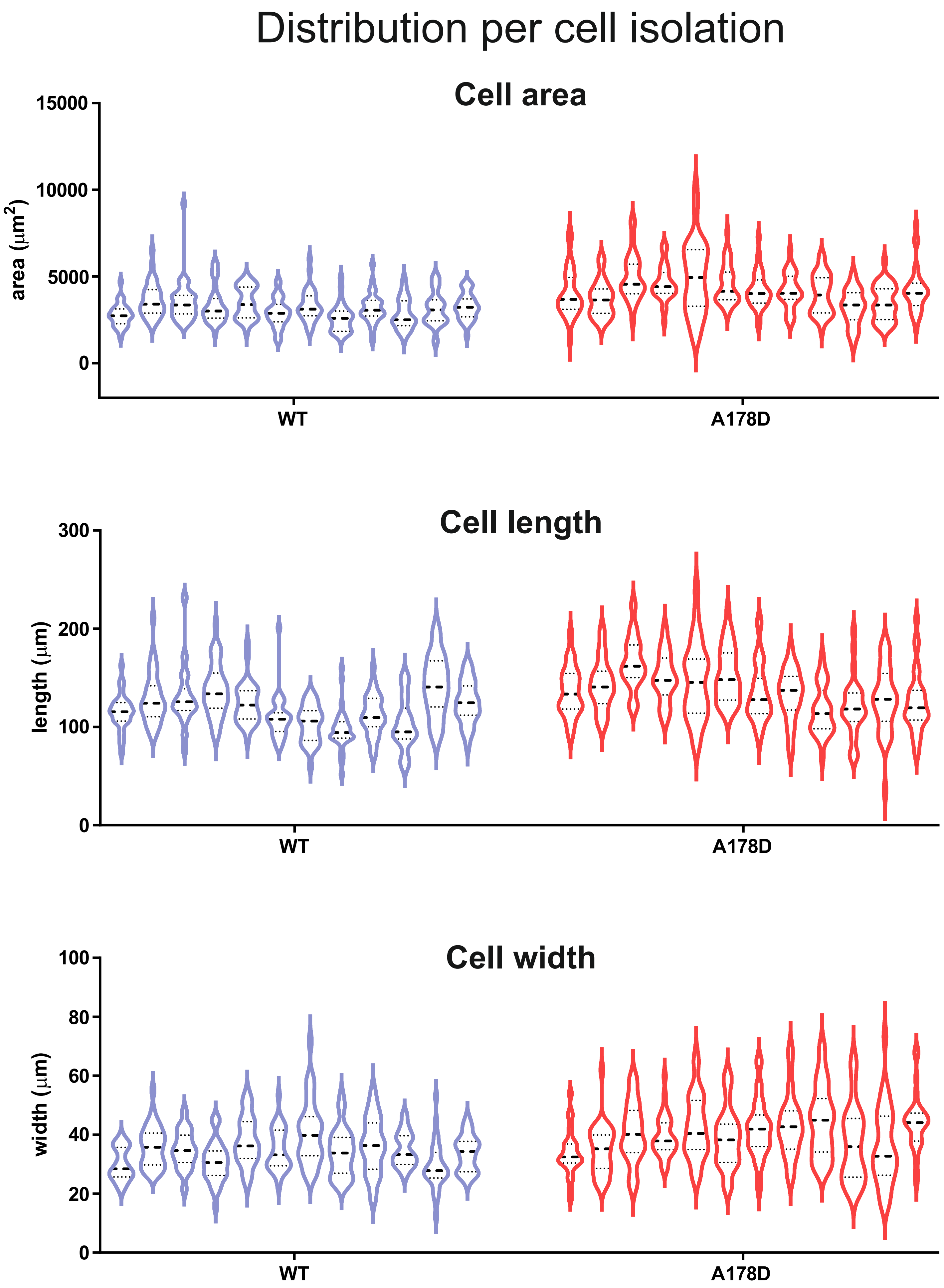

Distribution per cell isolation
Cell area
area (μm²)
15000
10000
5000
0
WT
A178D
Cell length
length (μm)
300
200
100
0
WT
A178D
Cell width
width (μm)
100
80
60
40
20
0
WT
A178D




Figure S10

**Figure S11:** A – Left: contractility traces of isolated cells from WT and A178D mice, there is no statistically significant difference in the traces (Student's t-test using hierarchical clustering). Right: Contractility traces normalised for resting sarcomere length are identical in WT and A178D cells. For further contractile parameters see Table S6. (WT: isolations from 12 hearts (4F/8M), age 128 ± 8 d; A178D isolations from 12 hearts (4F/8M), age 132 ± 10 d). B – Intracellular calcium ($[Ca^{2+}]_i$) transient measurements (35±1°C, 3Hz) were taken in Fura-2 loaded cardiomyocytes under perfusion with standard Tyrode solution + 1.4 mM $Ca^{2+}$. Top left: averaged $[Ca^{2+}]_i$ transient traces in cardiomyocytes isolated from WT or A178D mice, changes are not statistically significant. Top right: $[Ca^{2+}]_i$ diastolic levels were not significantly changed. The $[Ca^{2+}]_i$ transient amplitude (bottom left) and time decay constant (tau, bottom right) were equally unaltered; n=63/59 cardiomyocytes WT/A178D from 5 isolations each (2F/3M), age WT 147±7 d/ A178D 159±10 d. Student's t-test using hierarchical clustering (see Materials and Methods). Non-normally-distributed data (bottom panels) were log-transformed to achieve normality before statistical analysis was performed. Data are summarised in Table S7.

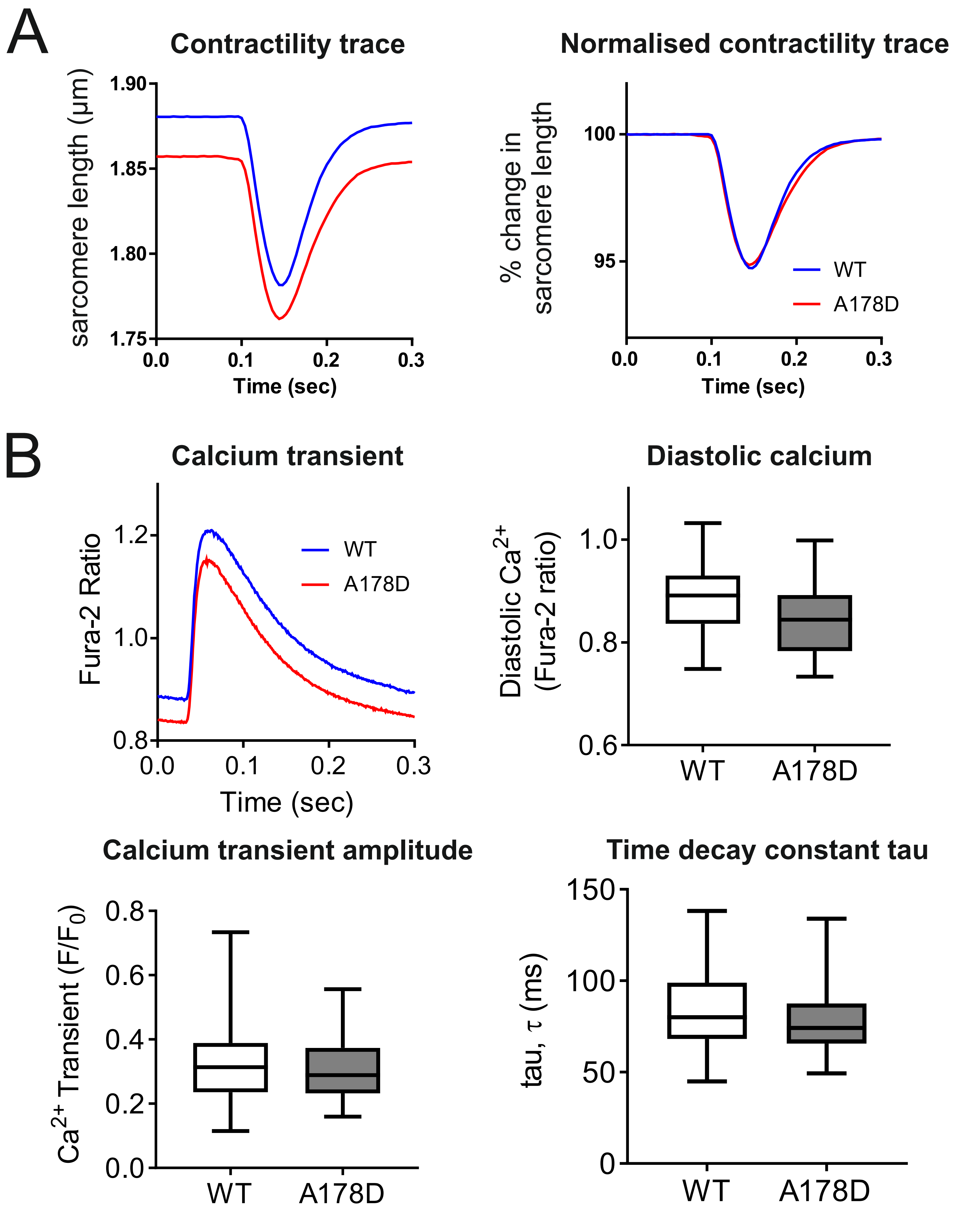
A
Contractility trace
sarcomere length (μm)
1.90
1.85
1.80
1.75
0.0
0.1
0.2
0.3
Time (sec)
Normalised contractility trace
% change in sarcomere length
100
95
WT
A178D
0.0
0.1
0.2
0.3
Time (sec)
B
Calcium transient
Fura-2 Ratio
1.2
1.0
0.8
WT
A178D
0.0
0.1
0.2
0.3
Time (sec)
Diastolic calcium
Diastolic Ca2+ (Fura-2 ratio)
1.0
0.8
0.6
WT
A178D
Calcium transient amplitude
Ca2+ Transient (F/F0)
0.8
0.6
0.4
0.2
0.0
WT
A178D
Time decay constant tau
tau, τ (ms)
150
100
50
0
WT
A178D

Figure S11

**Figure S12:** Passive tension measurements in loaded, demembranised fibres from WT and A178D hearts. A **–** Passive tension (mN/mm$^2$) was assessed by serial stretches. B - Titin derived fraction of total passive tension was assessed, C – After extraction of titin derived tension, the remaining tension was assumed ascribable to extracellular collagen. D – Titin derived fraction of total passive tension was assessed. All parameter were found to be normal (Student's t-test; n = 6, age WT 132 ± 2 d/A178D 129 ± 1 d, 4M/2F).

The plateau in titin derived tension (B), and reduced titin derived fraction of tension (D) at longer stretches reflect the known reduced dependency of titin in passive tension regulation at the higher sarcomere lengths [4].

For values at each stretch length, see Table S8.

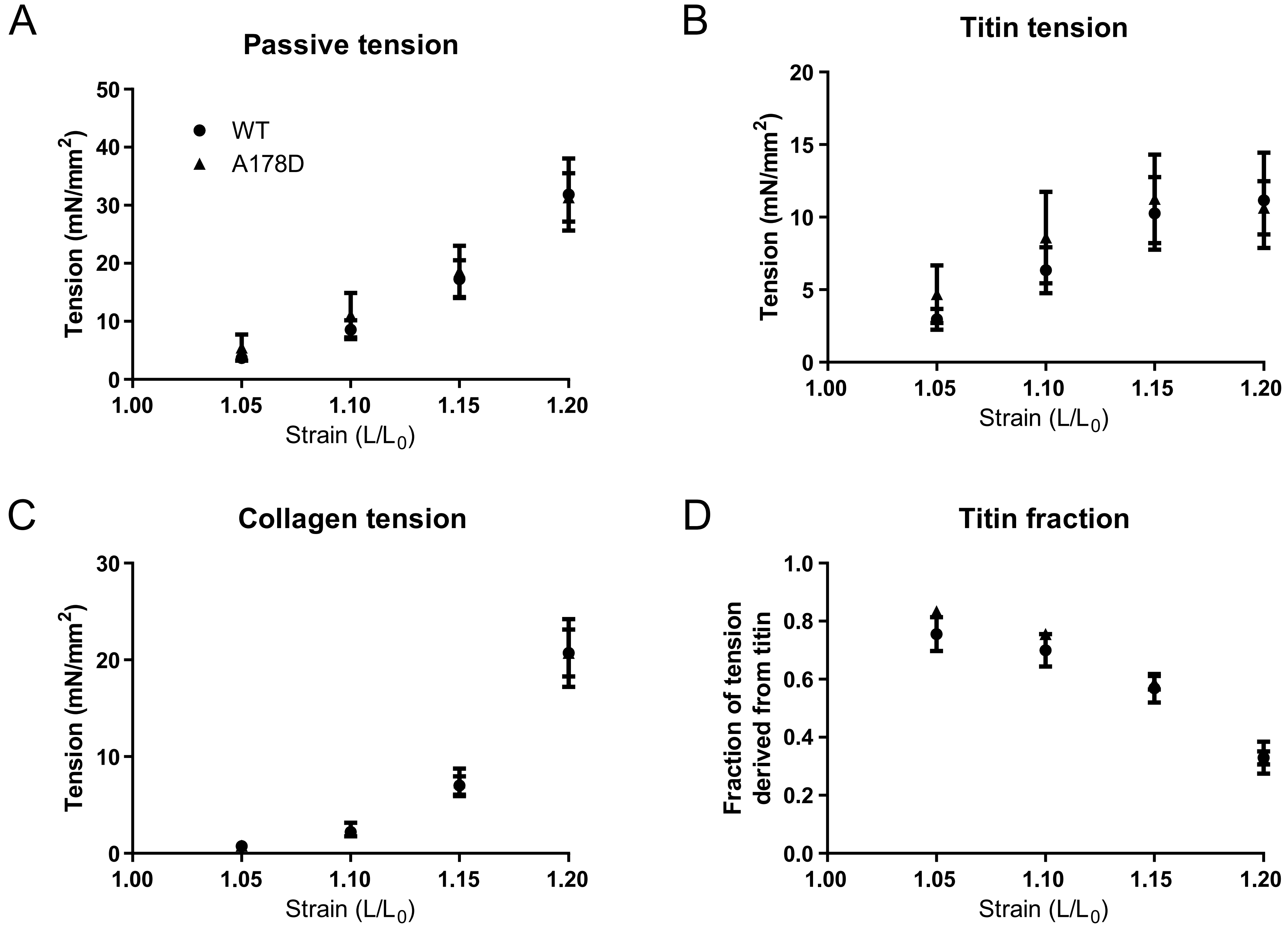
A
Passive tension
WT
A178D
Tension (mN/mm²)
Strain (L/L0)
B
Titin tension
Tension (mN/mm²)
Strain (L/L0)
C
Collagen tension
Tension (mN/mm²)
Strain (L/L0)
D
Titin fraction
Fraction of tension derived from titin
Strain (L/L0)



Figure S12

**Figure S13:** A – RNAseq results visualised as heat map showing the 50 significantly dysregulated transcripts with the largest fold change. Heat map shows genes with the strongest up-regulation in the A178D hearts at the top and the genes showing the strongest down-regulation at the bottom. Colours range from dark red to dark blue representing respectively the highest and lowest expression of a gene (n = 3/4 (WT/A178D), age: 135 d, all males; DESeq2 normalised counts). Please note that originally 4 WT samples were used, but WT2 was excluded because of poor library complexity. B – Gene set enrichment identified KEGG pathway "proteasome" was enriched in A178D hearts. Enrichment plots and heat maps are shown. The heat map shows genes in the KEGG pathways gene sets with the strongest up-regulation in the A178D hearts at the top and the genes showing the strongest down-regulation at the bottom. Colours range from dark red to dark blue representing respectively the highest and lowest expression of a gene. (n = 3/4 (WT/A178D), age: 135 d, all males; gene set enrichment analysis software, Broad Institute, with the KEGG pathways dataset; FDR q-val – false recovery rate, NES – normalised enrichment score).

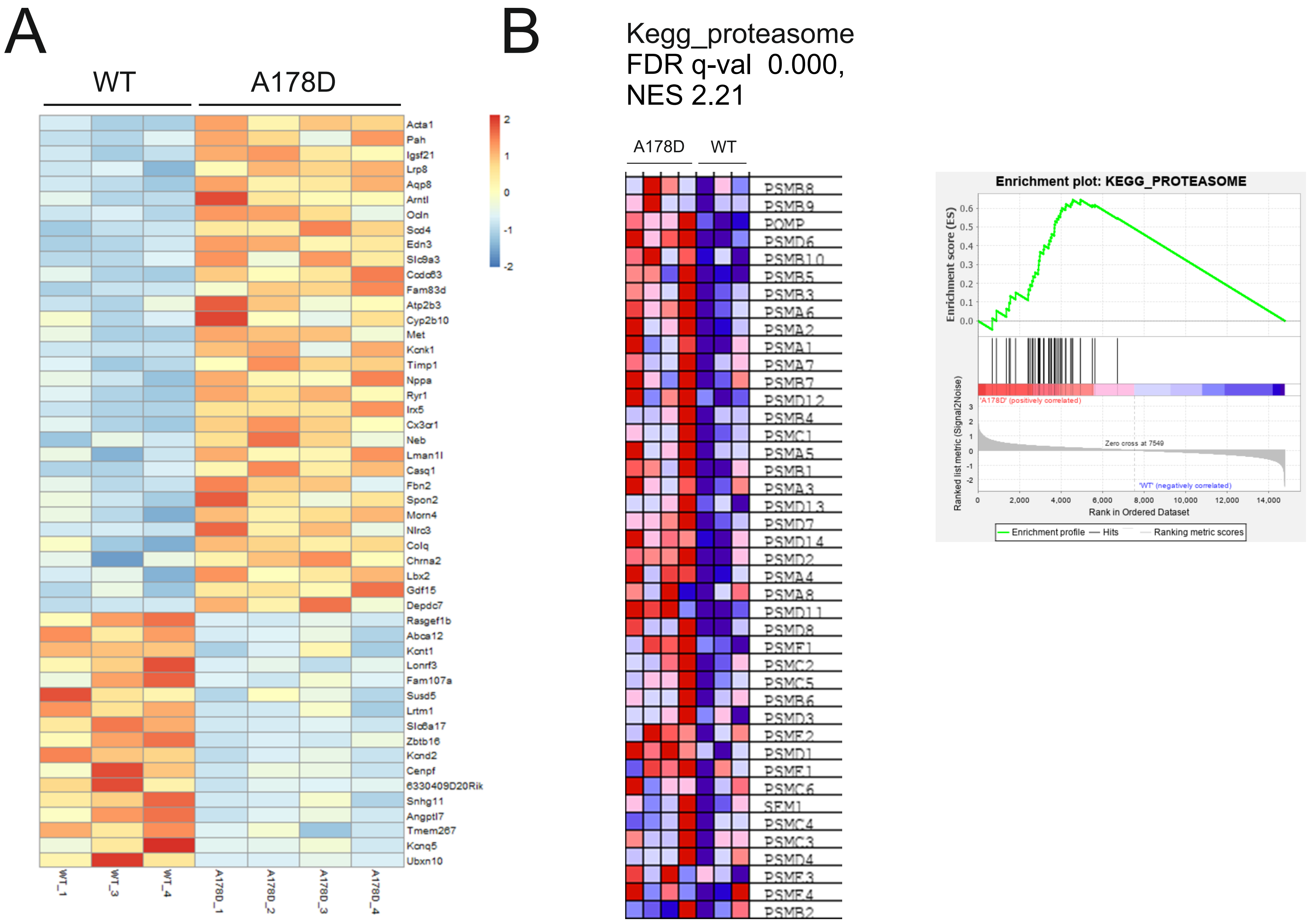
A
WT
A178D
Acta1
Pah
Igsf21
Lrp8
Aqp8
Arntl
Ocln
Scd4
Edn3
Slc9a3
Ccdc63
Fam83d
Atp2b3
Cyp2b10
Met
Kcnk1
Timp1
Nppa
Ryr1
Irx5
Cx3cr1
Neb
Lman1l
Casq1
Fbn2
Spon2
Morn4
Nlrc3
Colq
Chrna2
Lbx2
Gdf15
Depdc7
Rasgef1b
Abca12
Kcnt1
Lonrf3
Fam107a
Susd5
Lrtm1
Slc6a17
Zbtb16
Kcnd2
Cenpf
6330409D20Rik
Snhg11
Angptl7
Tmem267
Kcnq5
Ubxn10
2
1
0
-1
-2
WT_1
WT_3
WT_4
A178D_1
A178D_2
A178D_3
A178D_4
B
Kegg_proteasome
FDR q-val 0.000,
NES 2.21
A178D
WT
PSMB8
PSMB9
POMP
PSMD6
PSMB10
PSMB5
PSMB3
PSMA6
PSMA2
PSMA1
PSMA7
PSMB7
PSMD12
PSMB4
PSMC1
PSMA5
PSMB1
PSMA3
PSMD13
PSMD7
PSMD14
PSMD2
PSMA4
PSMA8
PSMD11
PSMD8
PSMF1
PSMC2
PSMC5
PSMB6
PSMD3
PSME2
PSMD1
PSME1
PSMC6
SEM1
PSMC4
PSMC3
PSMD4
PSME3
PSME4
PSMB2
Enrichment plot: KEGG_PROTEASOME
Enrichment score (ES)
0.6
0.5
0.4
0.3
0.2
0.1
0.0
'A178D' (positively correlated)
Zero cross at 7549
'WT' (negatively correlated)
Ranked list metric (Signal2Noise)
3
2
1
0
-1
-2
0
2,000
4,000
6,000
8,000
10,000
12,000
14,000
Rank in Ordered Dataset
Enrichment profile
Hits
Ranking metric scores

Figure S13

**Figure S14:** Top: Gross cardiac morphology shown on haematoxylin-eosin stained paraffin heart sections for WT and A178D. In higher magnification, haematoxylin-eosin (H&E, middle) as well as Picrosirius Red (bottom) stainings are normal. H&E: age 417 d, Picrosirius Red WT 429 d/A178D 417 d, all males.

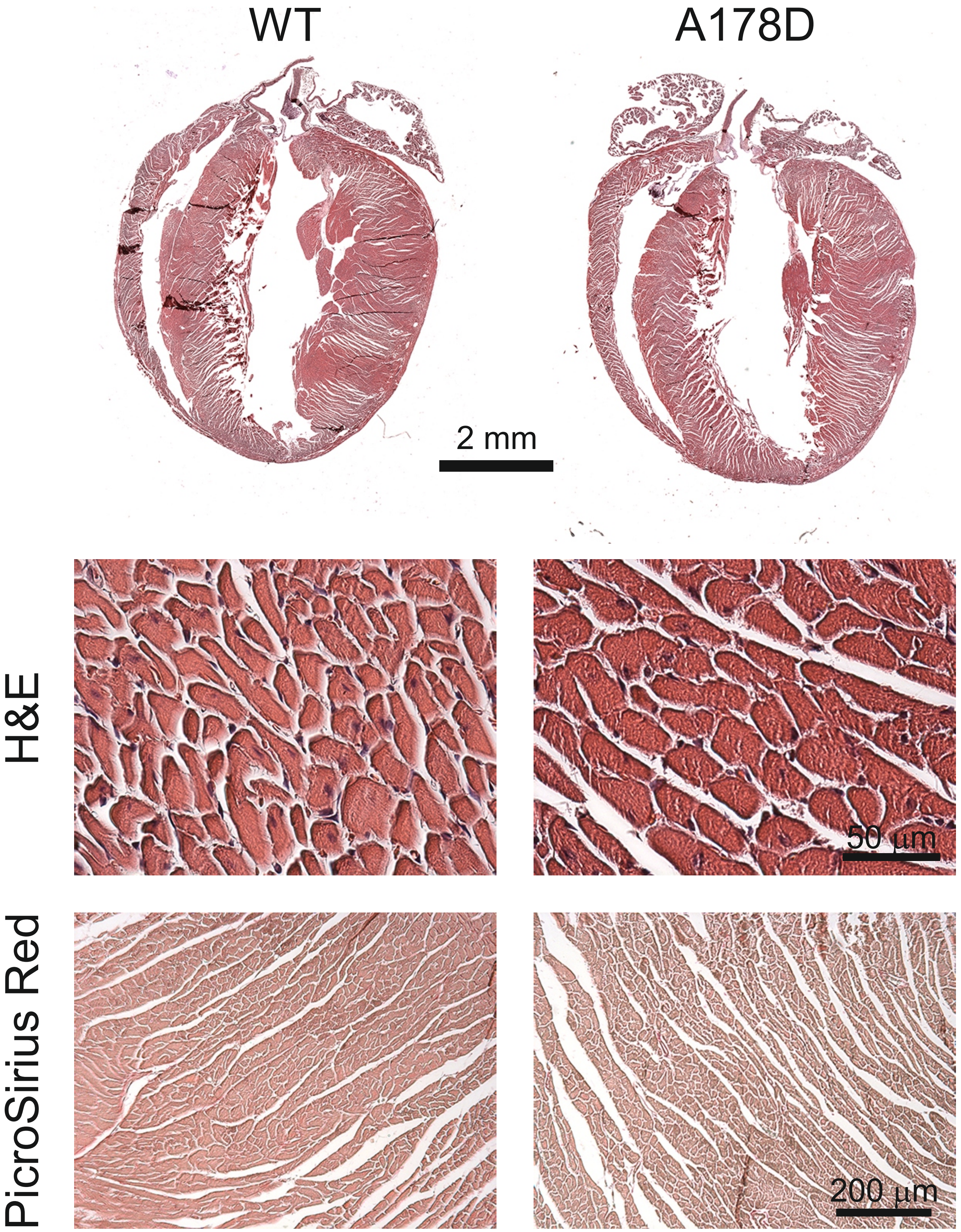


Figure S14

**Figure S15:** Assessment of transcriptional changes by qPCR for genes related to fetal gene programme (top), hypertrophic signalling (middle) and fibrosis (bottom) upon Iso/PE infusion. All measurements are normalised to *Gapdh*; (n = 6; Kruskal-Wallis followed by Wilcoxon rank sum test with Bonferroni correction). All mice in this experiment were male, age 120 ± 2 d.

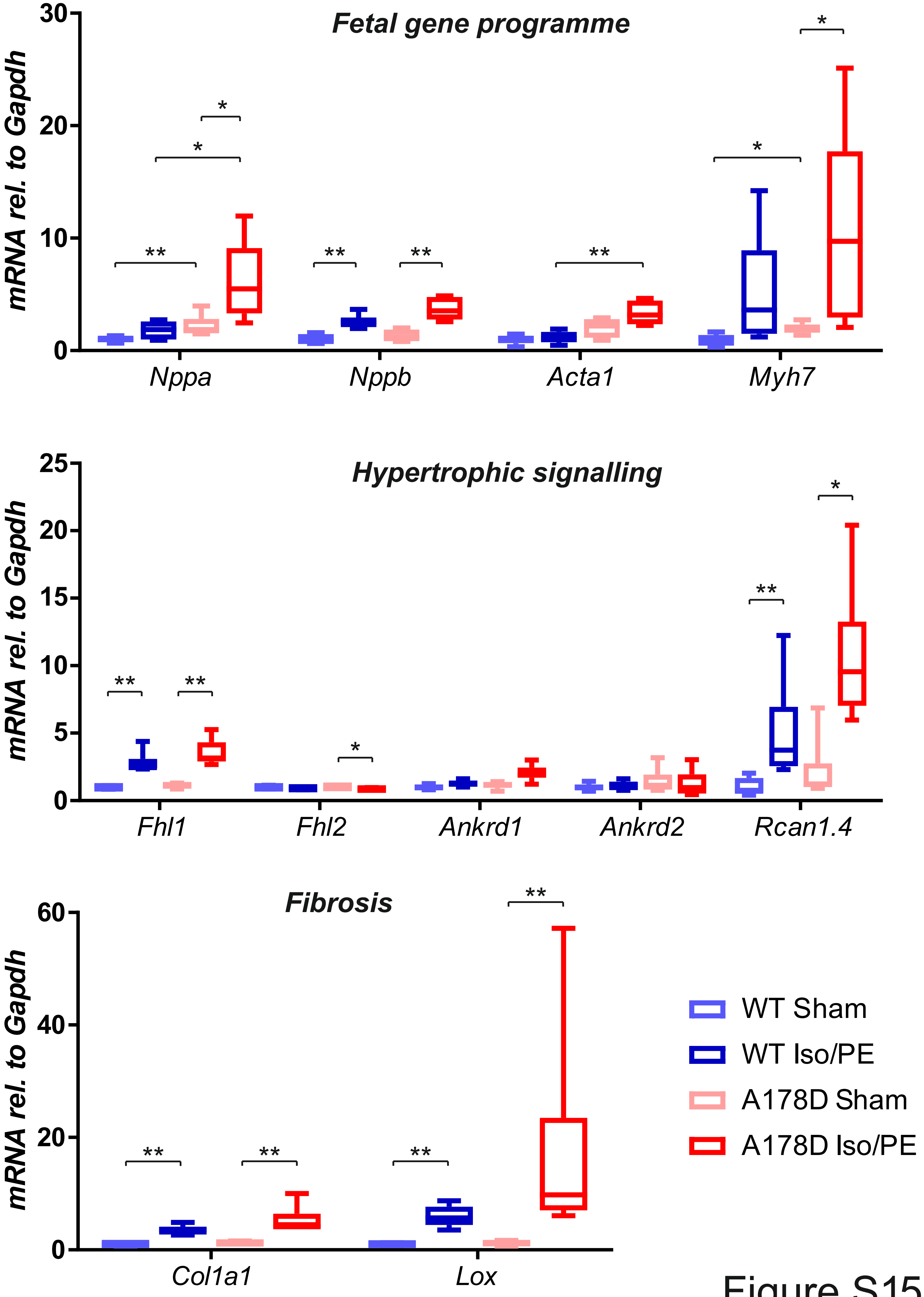
Fetal gene programme
mRNA rel. to Gapdh
30
20
10
0
Nppa
Nppb
Acta1
Myh7
**
*
*
**
**
**
*
*
Hypertrophic signalling
mRNA rel. to Gapdh
25
20
15
10
5
0
Fhl1
Fhl2
Ankrd1
Ankrd2
Rcan1.4
**
**
*
**
*
Fibrosis
mRNA rel. to Gapdh
60
40
20
0
Col1a1
Lox
**
**
**
**
WT Sham
WT Iso/PE
A178D Sham
A178D Iso/PE

Figure S15

**Figure S16:** Protein levels of beta-myosin in the three experimental cohorts A – 3 months old WT and A178D, B – 1 year old WT, HET and A178D and C – WT and A178D mice undergoing adrenergic challenge (Iso/PE, saline infusion as control = Sham). Blotting for Gapdh served as loading control (A, B), or the PonceauS stained membrane (C). Position of marker proteins (molecular weight in kD) is indicated. D – Quantification of Western blots, values are shown as median with interquartile range. Statistical tests: 3 months and 1 year – Mann-Whitney U-test, adrenergic challenge – Kruskal-Wallis followed by Wilcoxon rank sum test with Bonferroni correction, n = 6 per group.

Age: 3 months WT/A178D 113 ± 1 d, 1 year: WT/HET/A178D 416 ± 2 d/418± 2 d/423 ± 3 d; adrenergic challenge age: 120 ± 2 d, all males.

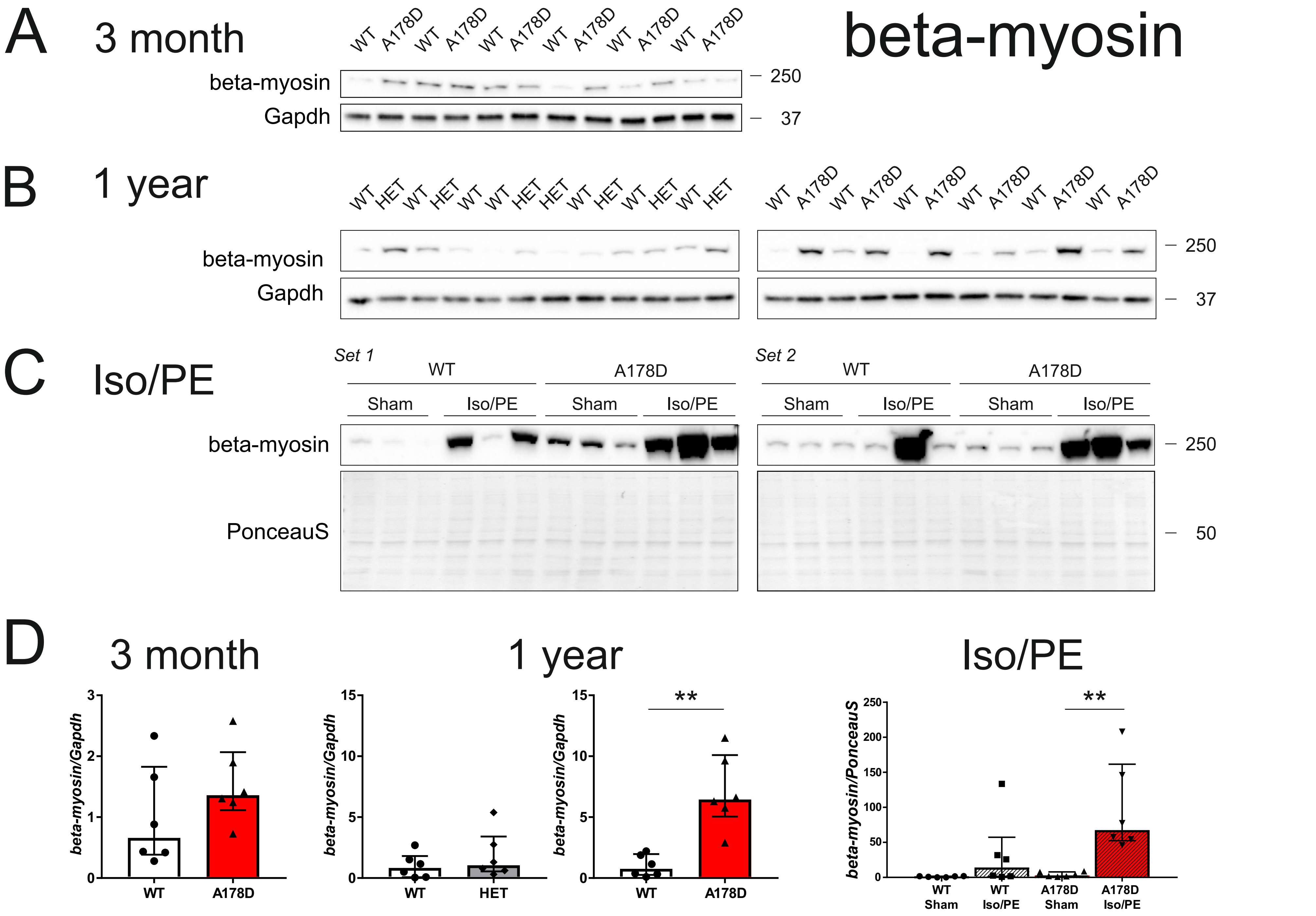

A
3 month
beta-myosin
WT A178D WT A178D WT A178D WT A178D WT A178D WT A178D
beta-myosin
Gapdh
250
37
B
1 year
WT HET WT HET WT WT HET HET WT HET WT HET WT HET
WT A178D WT A178D WT A178D WT A178D WT A178D WT A178D
beta-myosin
Gapdh
250
37
C
Iso/PE
Set 1
Set 2
WT
A178D
Sham
Iso/PE
beta-myosin
PonceauS
250
50
D
3 month
1 year
Iso/PE
beta-myosin/Gapdh
beta-myosin/PonceauS
WT
A178D
HET
**
WT Sham
WT Iso/PE
A178D Sham
A178D Iso/PE




Figure S16

**Figure S17:** Analysis for Fhl1 as in described in Figure S16. Please note, in the 3 months cohort (A), Fhl1 (arrow) was not detectable in WT or A178D samples, but clearly detectable in a positive control (‘C’ = Csrp3 null heart). * Gapdh signal bleed through on the membrane. D – 3 months cohort not quantified.

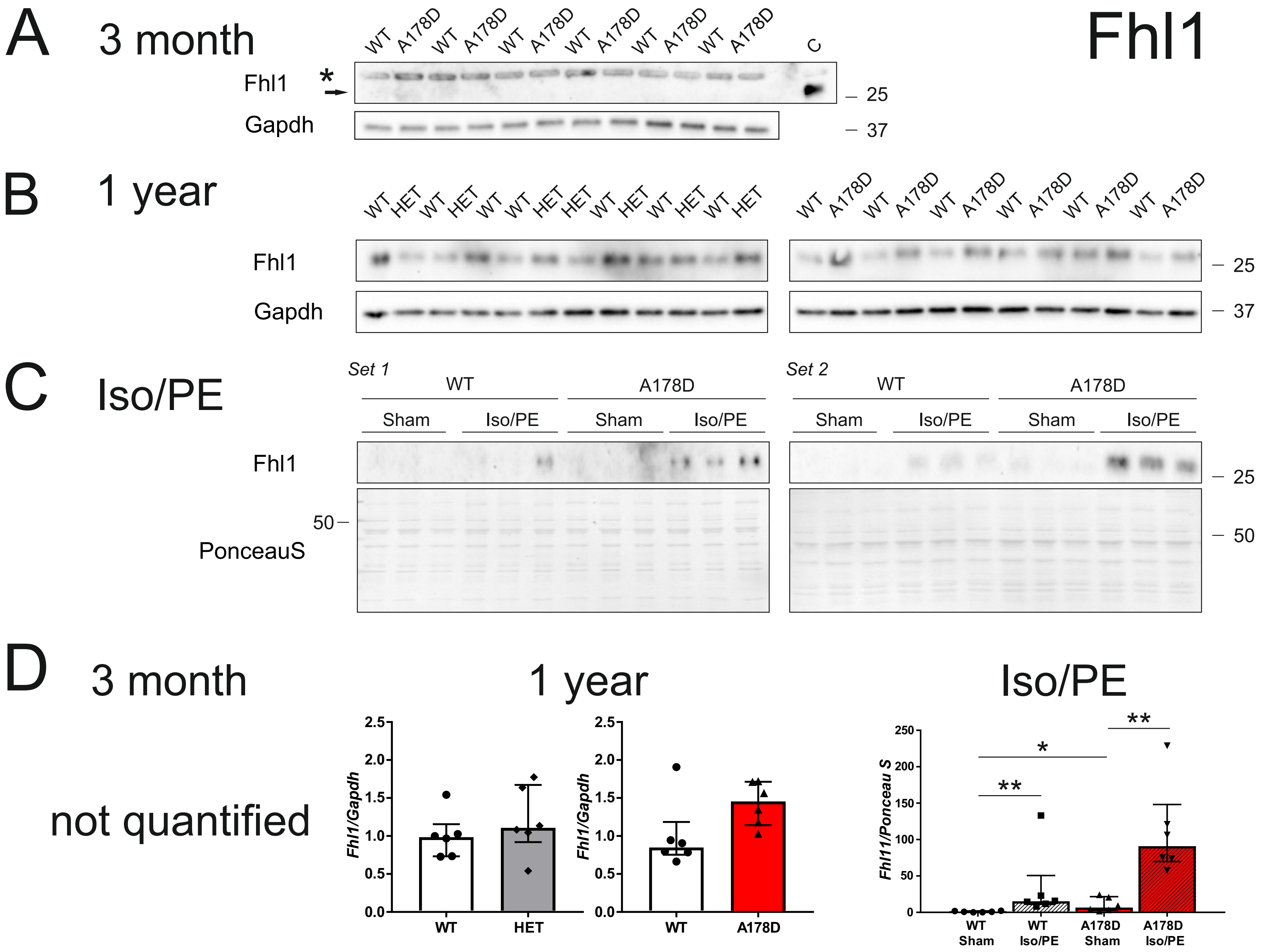

A
3 month
Fhl1
WT A178D WT A178D WT A178D WT A178D WT A178D WT A178D C
Fhl1
*
Gapdh
25
37
B
1 year
WT HET WT HET WT WT HET HET WT HET WT HET WT HET
WT A178D WT A178D WT A178D WT A178D WT A178D WT A178D
Fhl1
Gapdh
25
37
C
Iso/PE
Set 1
WT
A178D
Sham
Iso/PE
Sham
Iso/PE
Set 2
WT
A178D
Sham
Iso/PE
Sham
Iso/PE
Fhl1
25
50
PonceauS
50
D
3 month
not quantified
1 year
Iso/PE
Fhl1/Gapdh
WT
HET
WT
A178D
Fhl11/Ponceau S
WT Sham
WT Iso/PE
A178D Sham
A178D Iso/PE
**
*
**




Figure S17

**Figure S18:** Analysis for Ankrd1 as in described in Figure S16. Please note, in the 3 months cohort (A), Ankrd1 was not detectable in most samples, but clearly detectable in a positive control (‘C’ = Csrp3 null heart). In 1 year old mice (B), it was most readily detectable in A178D samples, D – 3 month and 1 year cohorts not quantified (too high background signals).

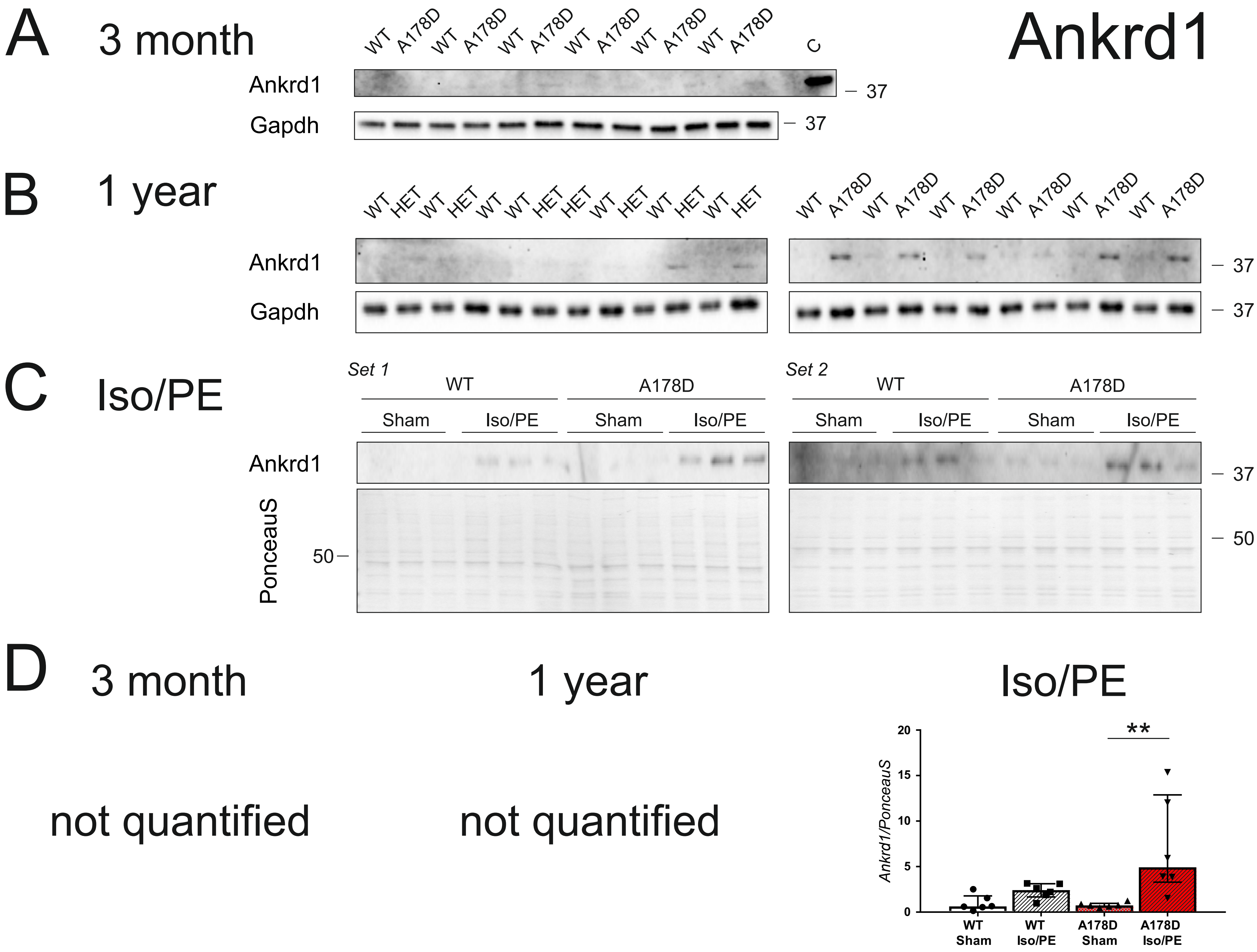

A
3 month
Ankrd1
WT
A178D
C
Ankrd1
Gapdh
37
B
1 year
WT
HET
A178D
Ankrd1
Gapdh
37
C
Iso/PE
Set 1
Set 2
WT
A178D
Sham
Iso/PE
Ankrd1
PonceauS
50
37
D
3 month
1 year
Iso/PE
not quantified
not quantified
Ankrd1/PonceauS
20
15
10
5
0
**
WT Sham
WT Iso/PE
A178D Sham
A178D Iso/PE




Figure S18

**Figure S19:** Analysis for Rcan1 as in Figure S16. Arrow indicates lane excluded from quantification due to high background.

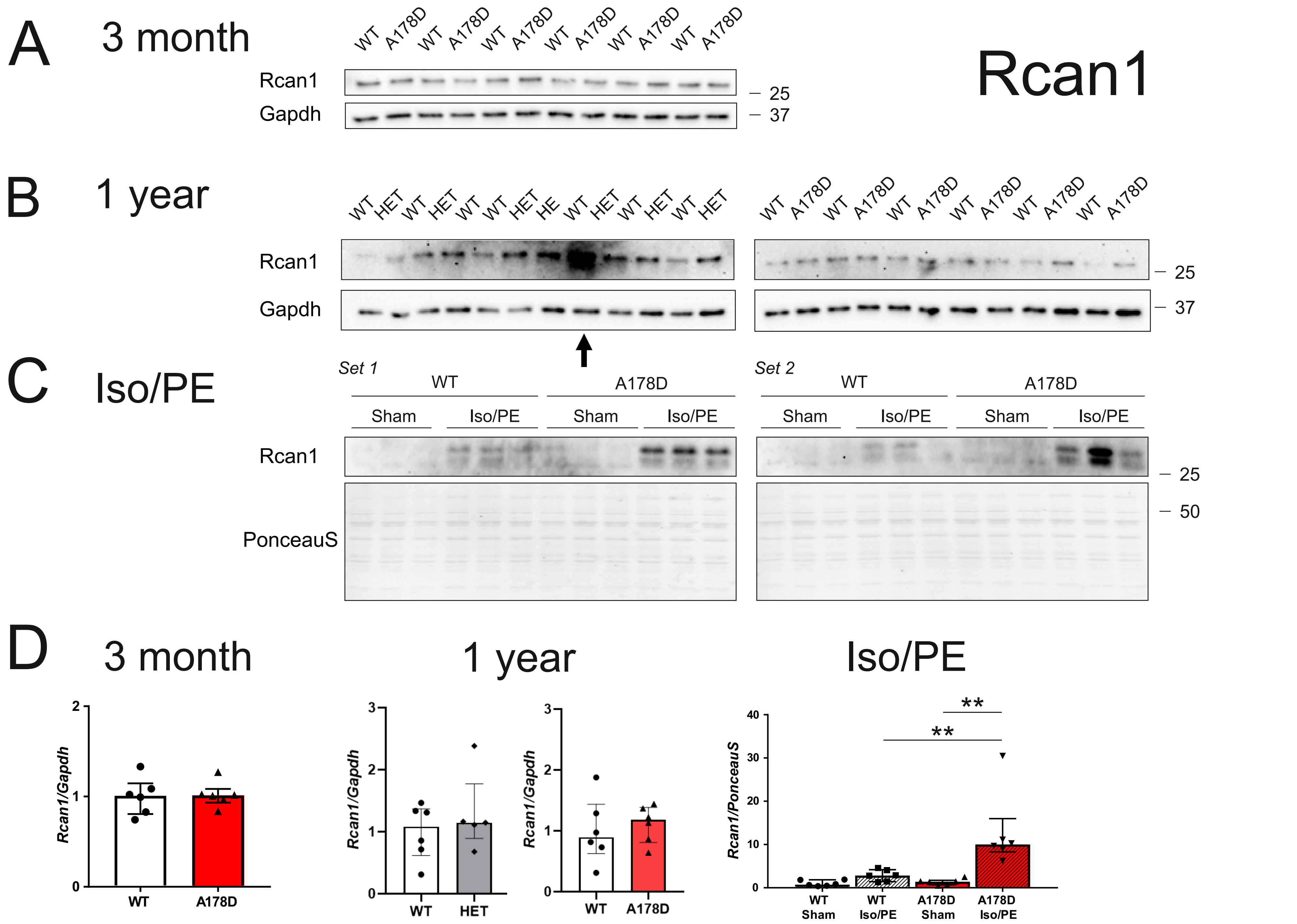

A
3 month
WT A178D WT A178D WT A178D WT A178D WT A178D WT A178D
Rcan1
Gapdh
25
37
Rcan1
B
1 year
WT HET WT HET WT WT HET HE WT HET WT HET WT HET
WT A178D WT A178D WT A178D WT A178D WT A178D WT A178D
Rcan1
Gapdh
25
37
C
Iso/PE
Set 1
WT
A178D
Sham
Iso/PE
Sham
Iso/PE
Set 2
WT
A178D
Sham
Iso/PE
Sham
Iso/PE
Rcan1
PonceauS
25
50
D
3 month
1 year
Iso/PE
Rcan1/Gapdh
WT
A178D
HET
Rcan1/PonceauS
WT Sham
WT Iso/PE
A178D Sham
A178D Iso/PE
**
**


Figure S19

**Figure S20:** Analysis for Cspr3/MLP as described in Figure S16.

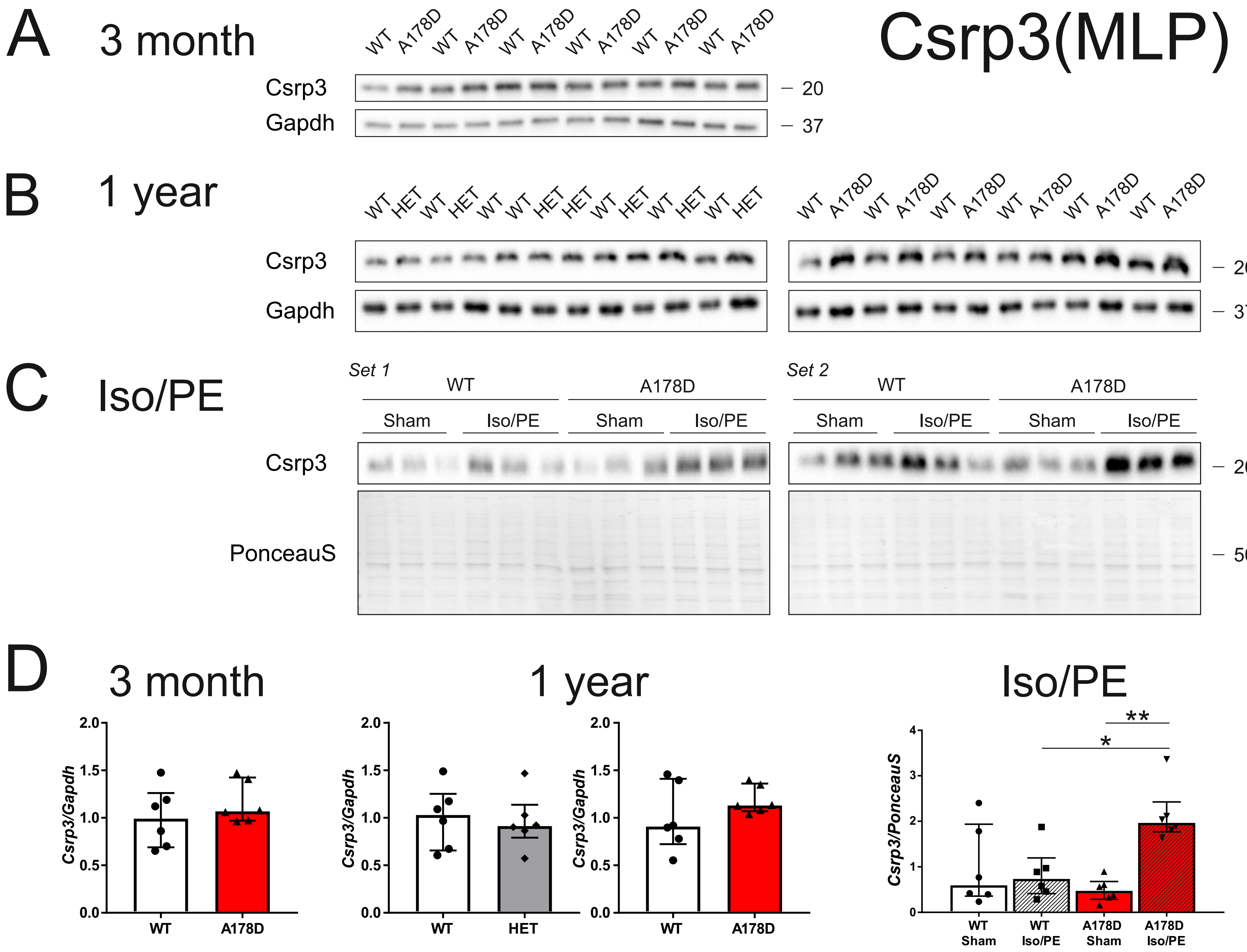

Csrp3(MLP)
A 3 month
WT A178D WT A178D WT A178D WT A178D WT A178D WT A178D
Csrp3
Gapdh
20
37
B 1 year
WT HET WT HET WT WT HET HET WT HET WT HET WT HET
WT A178D WT A178D WT A178D WT A178D WT A178D WT A178D
Csrp3
Gapdh
20
37
C Iso/PE
Set 1
Set 2
WT
A178D
Sham
Iso/PE
Csrp3
PonceauS
20
50
D
3 month
1 year
Iso/PE
Csrp3/Gapdh
Csrp3/PonceauS
2.0
1.5
1.0
0.5
0.0
4
3
2
1
0
WT
A178D
HET
WT Sham
WT Iso/PE
A178D Sham
A178D Iso/PE
**
*




Figure S20

**Figure S21:** A – Controls for protein fractionation (see Supplementary Methods): Cytoplasmic (cyto) and myofilament (myof) protein fractions were prepared from WT and A178D hearts and blotted for cytoplasmic protein Gapdh and the myofilament protein alpha-actinin, both proteins are found exclusively in the expected fractions. (n = 2, 112 d, male). B – Tandem ubiquitin binding entities (TUBE) assay: Ubiquitinylated proteins were pulled down from A178D heart lysate (treated *in vivo* with proteasomal inhibitor epoxomicin, 113 d, male) using immobilised tandem ubiquitin-binding entities (TUBE). Agarose matrix without TUBE served as control (ctr). Lysate controls are shown (LC, 1 % of input). Blotting for ubiquitin indicates the enrichment of ubiquitinylated proteins in the TUBE pulldown. Blotting for Fhl2 shows specific pulldown of the protein with TUBE, indicating ubiquitinylation. C – Fhl2 downregulation in myofilament protein fraction. Left - Western blot demonstrating the downregulation of Fhl2 in myofilament protein samples of A178D mice. Alpha-actinin and PonceauS stain serve as loading controls. Right – Quantification of blot (normalized to PonceauS stain) indicates down-regulation of Fhl2 by approximately 50 % in the myofilament protein fraction; (n = 6, age WT 114 ± 1 d/A178D 113 ± 1 d, 3M/3F; Student's t-test). D – Immunofluorescence of Fhl2 in isolated cardiomyocytes from WT and A178D mice (red), counter-stained with Z-disc marker alpha-actinin (green), and merged images. Insert at the top left: magnification of an area of the cell. Fhl2 is down-regulated at the sarcomeres in A178D cells, but no aggregates are observed. Age WT: 97 d, A178D 98 d, both male.

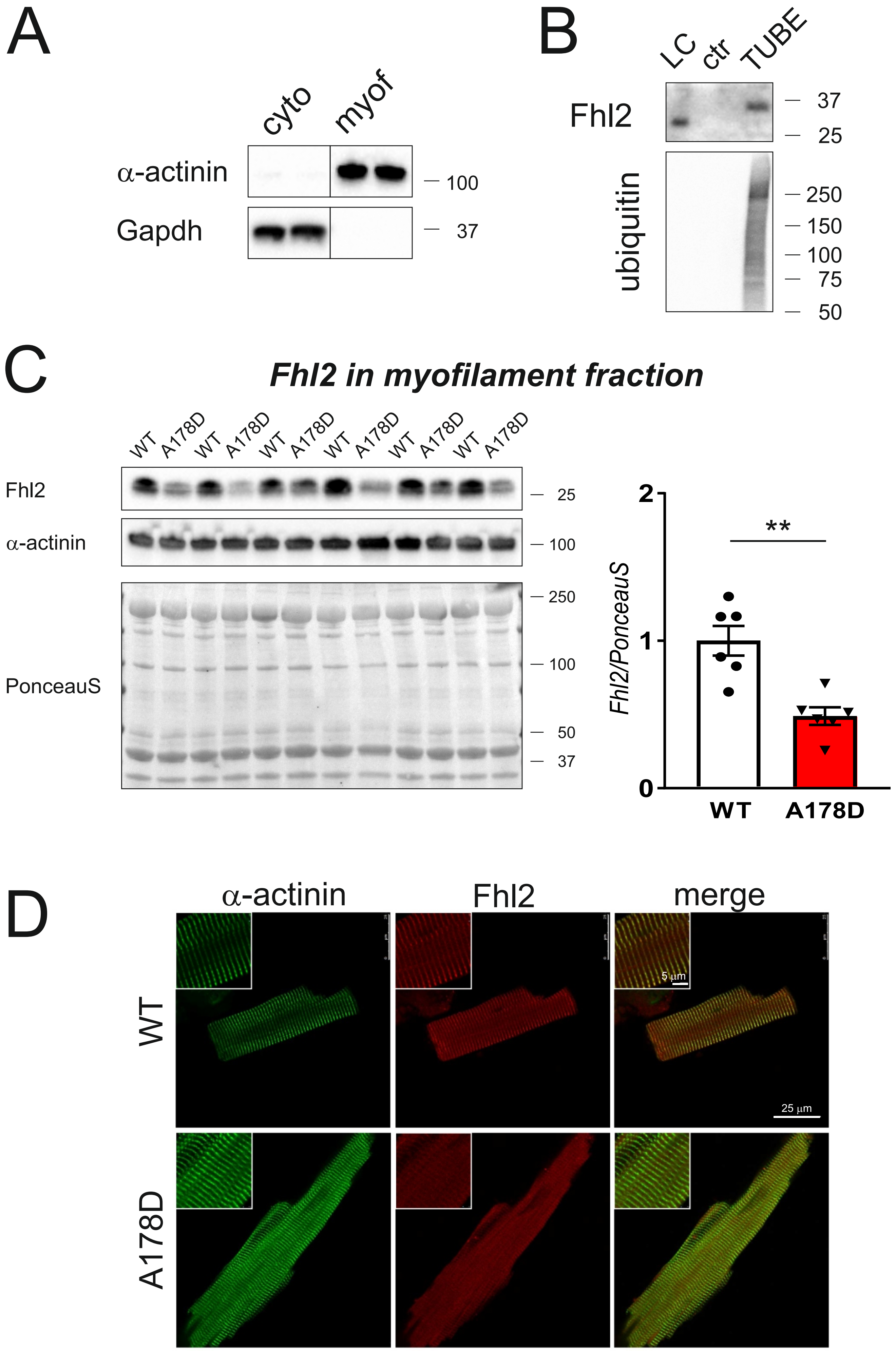
A
cyto
myof
α-actinin
Gapdh
100
37
B
LC
ctr
TUBE
Fhl2
ubiquitin
37
25
250
150
100
75
50
C
Fhl2 in myofilament fraction
WT
A178D
Fhl2
α-actinin
PonceauS
25
100
250
100
50
37
Fhl2/PonceauS
2
1
0
**
WT
A178D
D
α-actinin
Fhl2
merge
WT
A178D
5 µm
25 µm



Figure S21

**Figure S22:** Induction of Mlf1 upon Iso/PE infusion. A – Western blots are shown; PonceauS stain serves as loading control. B – Quantification of blots indicates more pronounced induction of Mlf1 in A178D hearts, independent of adrenergic stimulation. (n = 6/5 WT/A178D, 120 ± 2 d, all males; Kruskal-Wallis followed by Wilcoxon rank sum test with Bonferroni correction; arrow indicates band excluded from quantification).

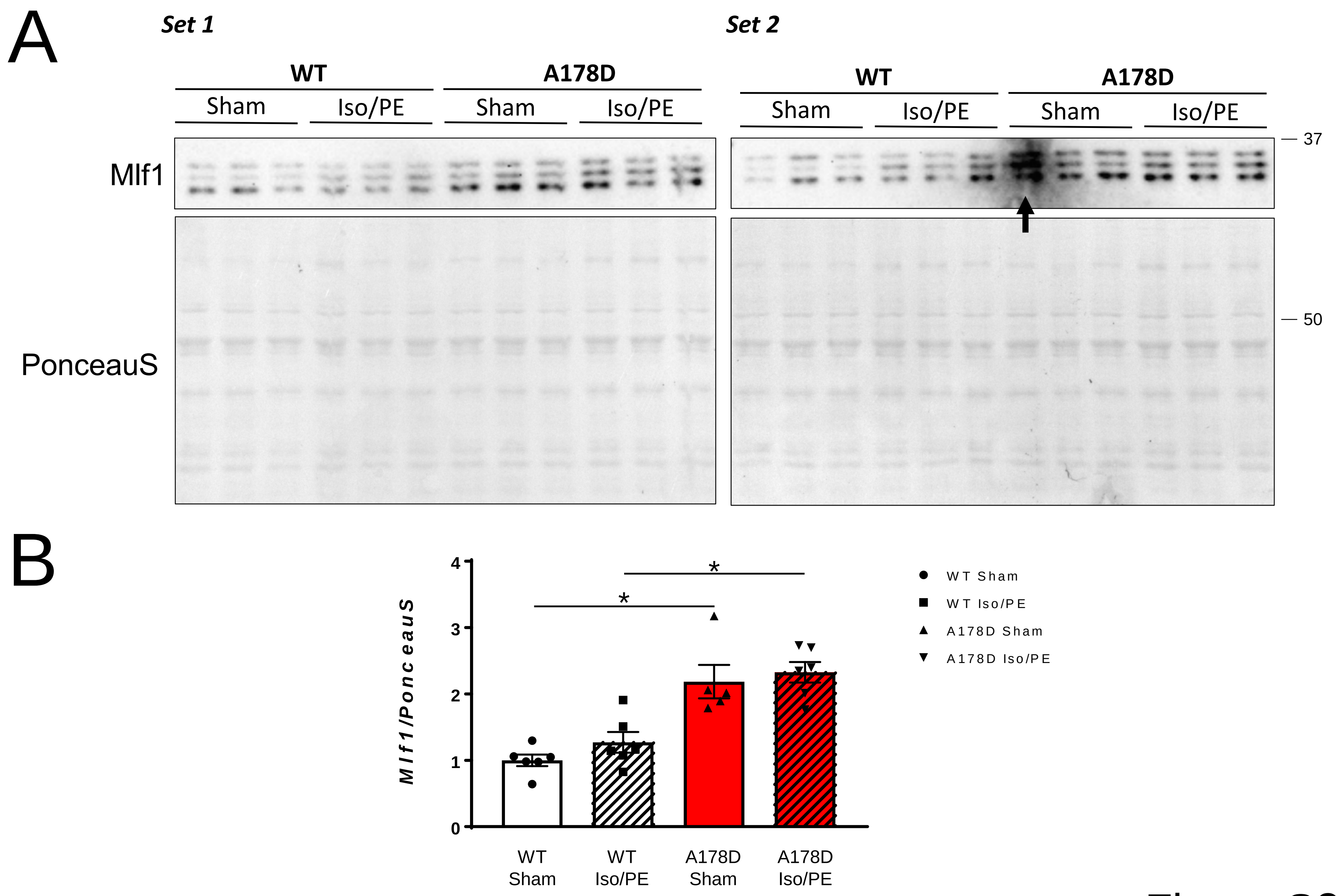
A
Set 1
Set 2
WT
A178D
WT
A178D
Sham
Iso/PE
Sham
Iso/PE
Sham
Iso/PE
Sham
Iso/PE
Mlf1
PonceauS
37
50
B
Mlf1/PonceauS
0
1
2
3
4
*
*
WT Sham
WT Iso/PE
A178D Sham
A178D Iso/PE
WT Sham
WT Iso/PE
A178D Sham
A178D Iso/PE



Figure S22

**Figure S23:** A – Tandem ubiquitin binding entities (TUBE) assay: Ubiquitinylated proteins were pulled down from A178D heart lysate (treated *in vivo* with proteasomal inhibitor epoxomicin, 113 d, male) using immobilised tandem ubiquitin-binding entities (TUBE). Agarose matrix without TUBE served as control (ctr). Lysate controls are shown (LC, 1 % of input). Blotting for ubiquitin indicates the enrichment of ubiquitinylated proteins in the TUBE pulldown. Blotting for telethonin shows specific pulldown of the protein with TUBE, indicating ubiquitinylation.

B-D**:** Reduced protein levels of telethonin in 1 year old A178D mice (B) and in A178D undergoing adrenergic challenge or sham treatment (C). Blotting for Gapdh served as loading control (B), or the PonceauS stained membrane (C). Position of marker proteins (molecular weight in kD) is indicated. D – Quantification of Western blots, values are shown as median with interquartile range. Statistical tests: 1 year – Mann-Whitney U-test, adrenergic challenge – Kruskal-Wallis followed by Wilcoxon rank sum test with Bonferroni correction, n=6 per group. Age: 1 year: WT/HET/A178D 416 ± 2 d/418± 2 d/423 ± 3 d; adrenergic challenge age: 120 ± 2 d, all males.

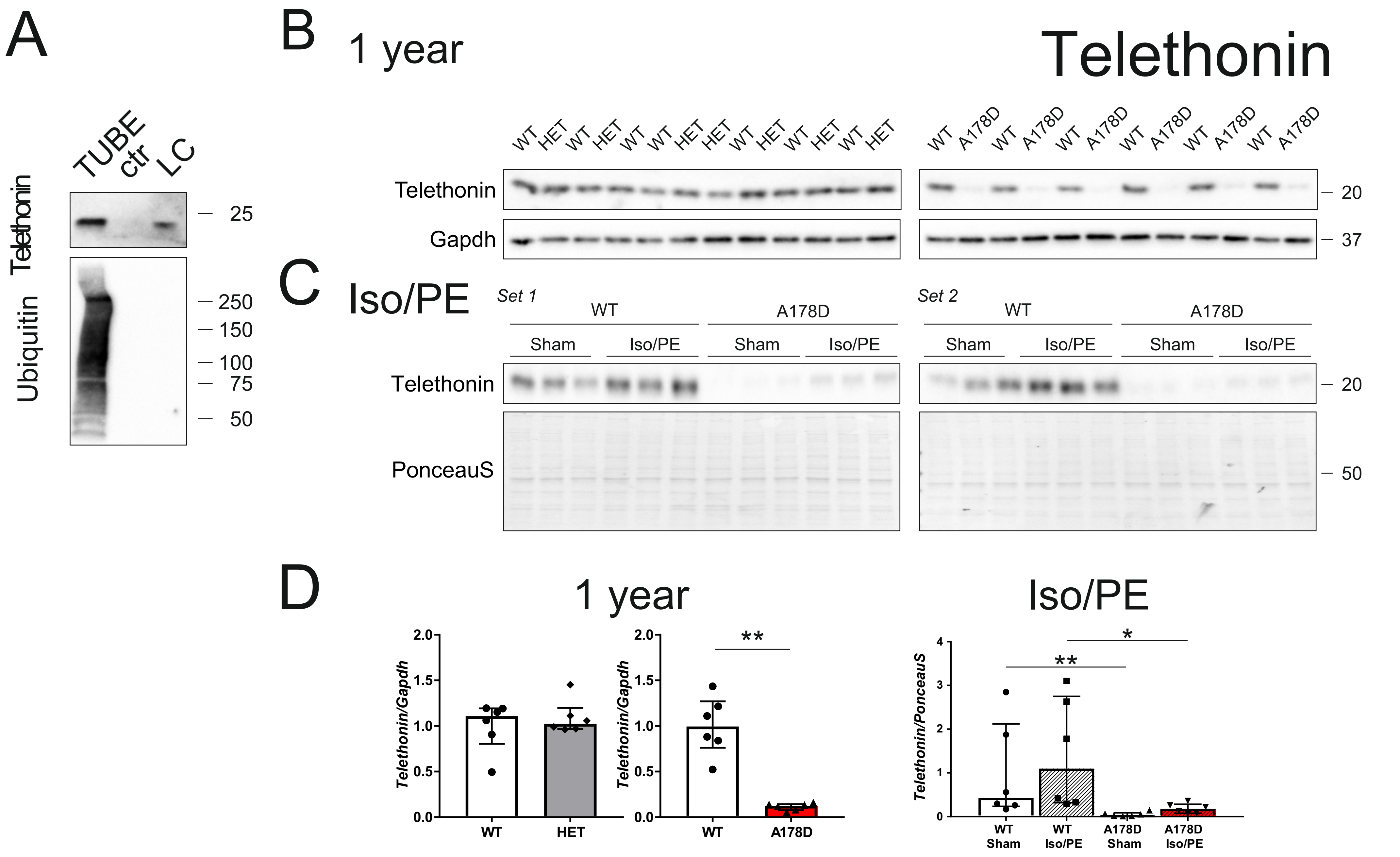
A
TUBE
ctr
LC
Telethonin
Ubiquitin
25
250
150
100
75
50
B
1 year
Telethonin
WT HET WT HET WT WT HET HET WT HET WT HET WT HET
WT A178D WT A178D WT A178D WT A178D WT A178D WT A178D
Telethonin
Gapdh
20
37
C
Iso/PE
Set 1
Set 2
WT
A178D
Sham
Iso/PE
Telethonin
PonceauS
20
50
D
1 year
Iso/PE
Telethonin/Gapdh
Telethonin/PonceauS
WT
HET
A178D
**
*
WT Sham
WT Iso/PE
A178D Sham
A178D Iso/PE



Figure S23

**Figure S24:** Top – Cryo-sections of hearts from WT mice, stained for titin Z-disc epitope Z1Z2 and Fhl2; nuclei are visualised with DAPI. Fhl2 displays a sarcomeric stain. Middle – Same staining in A178D hearts treated with vehicle (10 % dimethyl sulfoxide in 0.9 % saline). Fhl2 signal is diminished at the sarcomeres. Bottom – A178D hearts treated with epoxomicin (0.5 mg $kg^{-1}$ bodyweight per day for 7 days) show partial restoration of Fhl2 signal at the sarcomeres. Scale bar represents 25 microns. All mice in this experiment are male (n=2 per group; WT age 116 d, A178D age 115 ± 2 d).

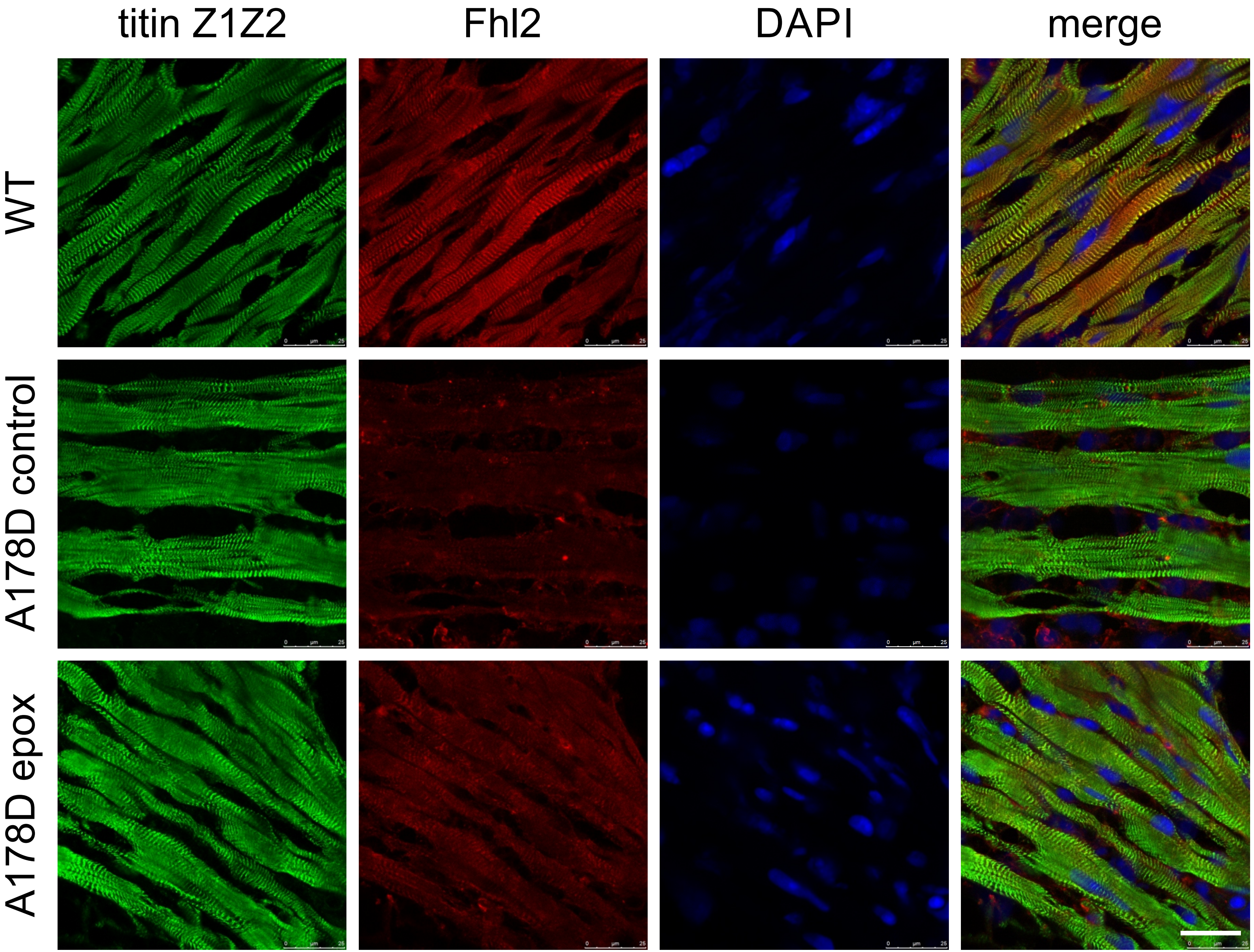




Figure S24

**Figures S25:** Assessment of changes in autophagy marker p62 as in described in Figure S16.

The visual trend in C does not reach statistical significance.

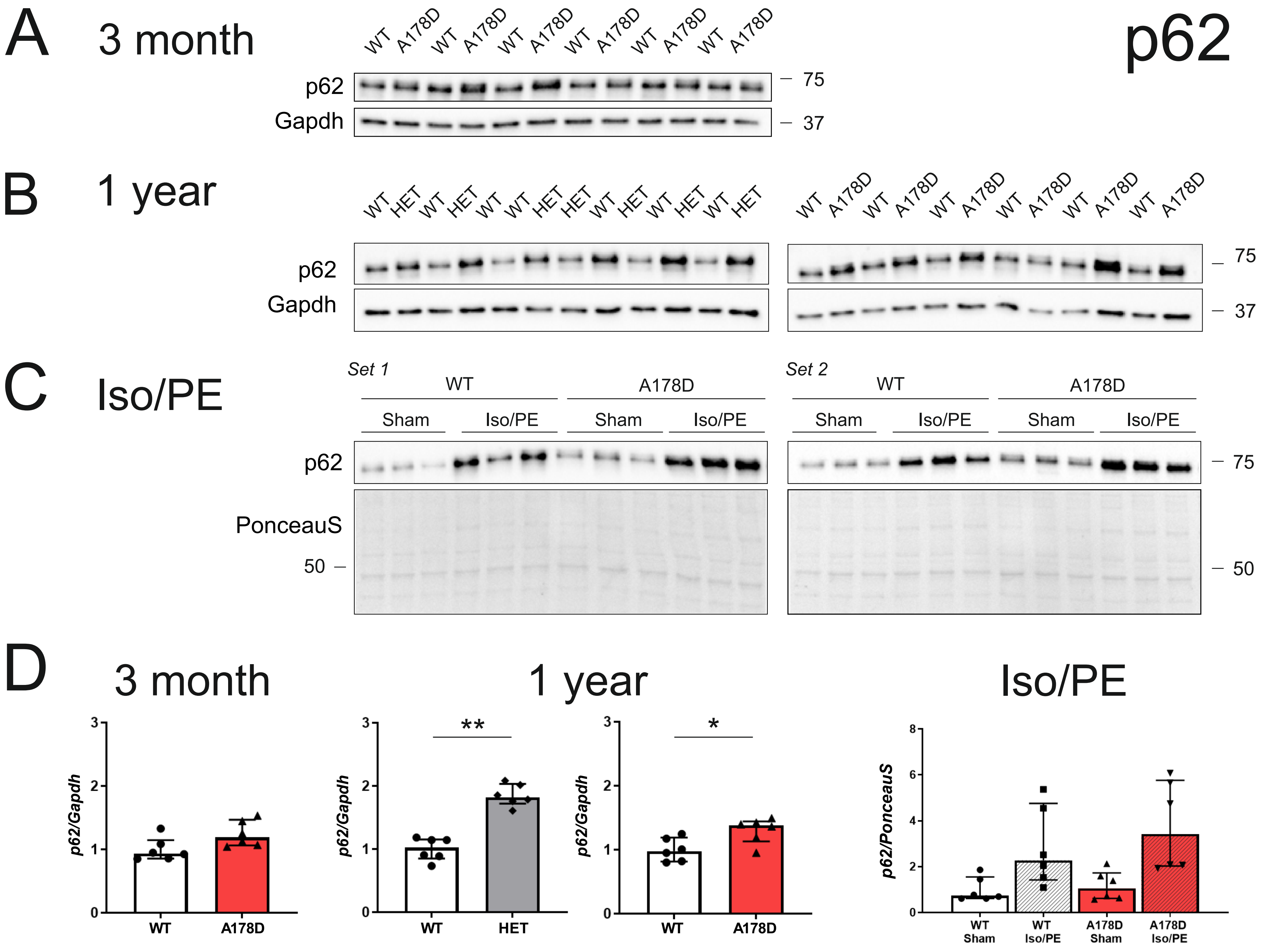
A
3 month
p62
WT A178D WT A178D WT A178D WT A178D WT A178D WT A178D
p62
Gapdh
75
37
B
1 year
WT HET WT HET WT WT HET HET WT HET WT HET WT HET
WT A178D WT A178D WT A178D WT A178D WT A178D WT A178D
p62
Gapdh
75
37
C
Iso/PE
Set 1
Set 2
WT
A178D
Sham
Iso/PE
p62
PonceauS
75
50
D
3 month
1 year
Iso/PE
p62/Gapdh
p62/PonceauS
WT
A178D
HET
**
*
WT Sham
WT Iso/PE
A178D Sham
A178D Iso/PE

Figure S25

**Figure S26:** A – Phostag blot reveals normal phosphorylation of the residual telethonin in A178D hearts. The position of non-phosphorylated (P0), mono-phosphorylated (P1) and bis-phosphorylated (P2) species is indicated. Please note, more lysate (8-fold) was loaded for the A178D samples (n = 2, age 113 ± 1 d, males). B – Quantification of percentage of P2 and P1 in WT and A178D hearts (no P0 was detectable). C – Normal T-tubular organisation in A178D cardiomyocytes visualised by di-4-ANNEPPS staining (age 102 d, male).

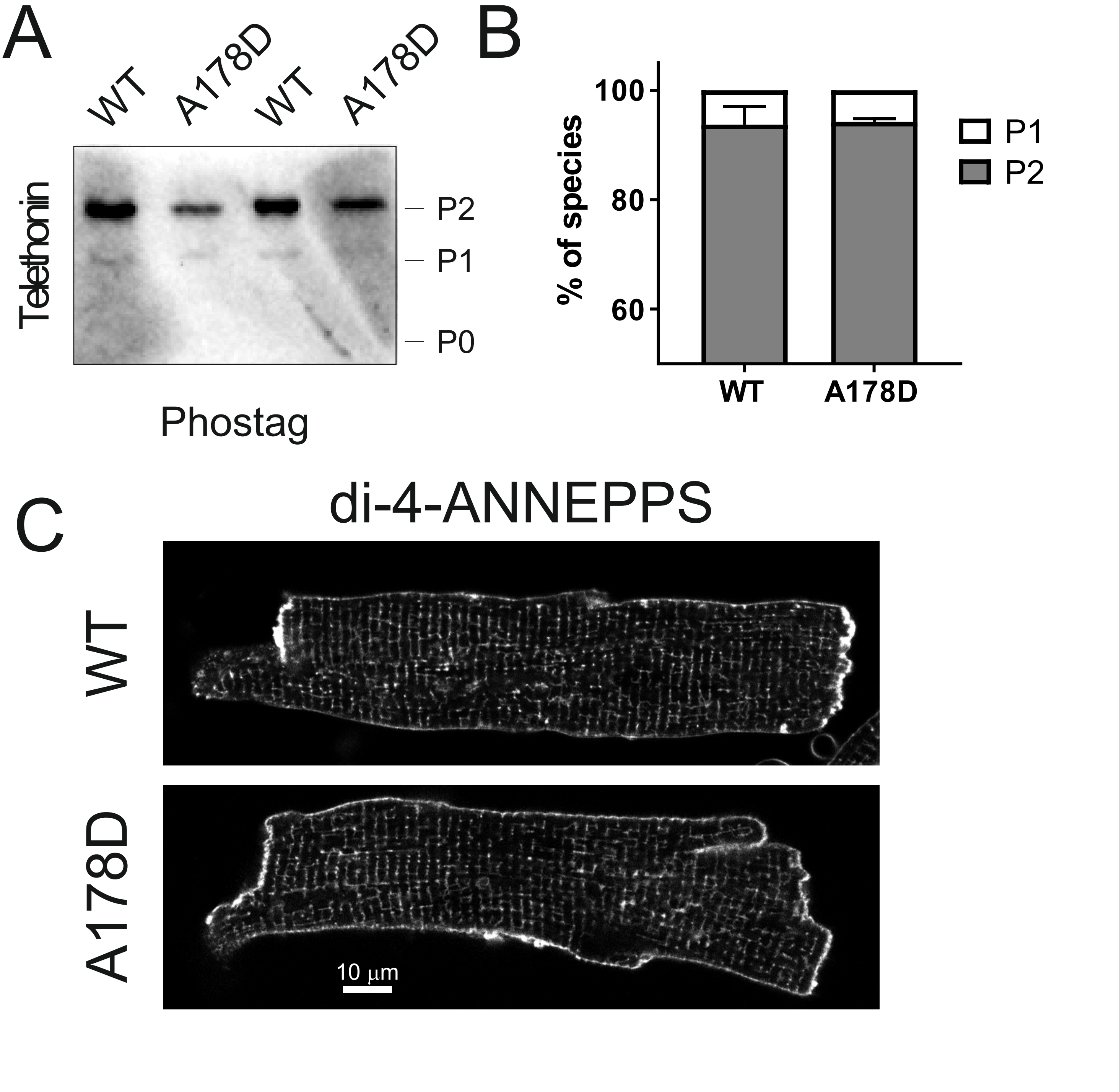
A
WT
A178D
WT
A178D
Telethonin
P2
P1
P0
Phostag
B
% of species
100
80
60
WT
A178D
P1
P2
C
di-4-ANNEPPS
WT
A178D
10 µm



Figure S26

**Figure S27:** No evidence of increased apoptosis in A178D hearts: A – Assessment of transcriptional changes by qPCR for genes related to apoptosis in 3 month old hearts. All measurements are normalised to *Gapdh*; (Mann-Whitney U-test, WT: n = 10, 4M/6F, age 114 ± 1 d, A178D: n = 12, 6M/6F, age 113 ± 1 d). B – p53 expression is normal in A178D hearts when assessed by Western blotting. Gapdh serves as loading control and the band corresponding to p53 is indicated by an arrow. (n = 4, age 113 ± 1 d, all males). C – Assessment of transcriptional changes by qPCR for genes related to apoptosis in 1 year old hearts. All measurements are normalised to *Gapdh*; (Kruskal-Wallis followed by Dunn's multiple comparison's test; WT: n = 5, age 411 ± 3 d, HET n = 6, age 412 ± 2 d, A178D n = 6, age 423 ± 3 d, all male). D – Assessment of transcriptional changes by qPCR for genes related to apoptosis upon Iso/PE infusion. All measurements are normalised to *Gapdh*; (n = 6; age 120 ± 2 d, all males; Kruskal-Wallis followed by Wilcoxon rank sum test with Bonferroni correction). There is no indication of a stronger response to Iso/PE infusion in A178D hearts.

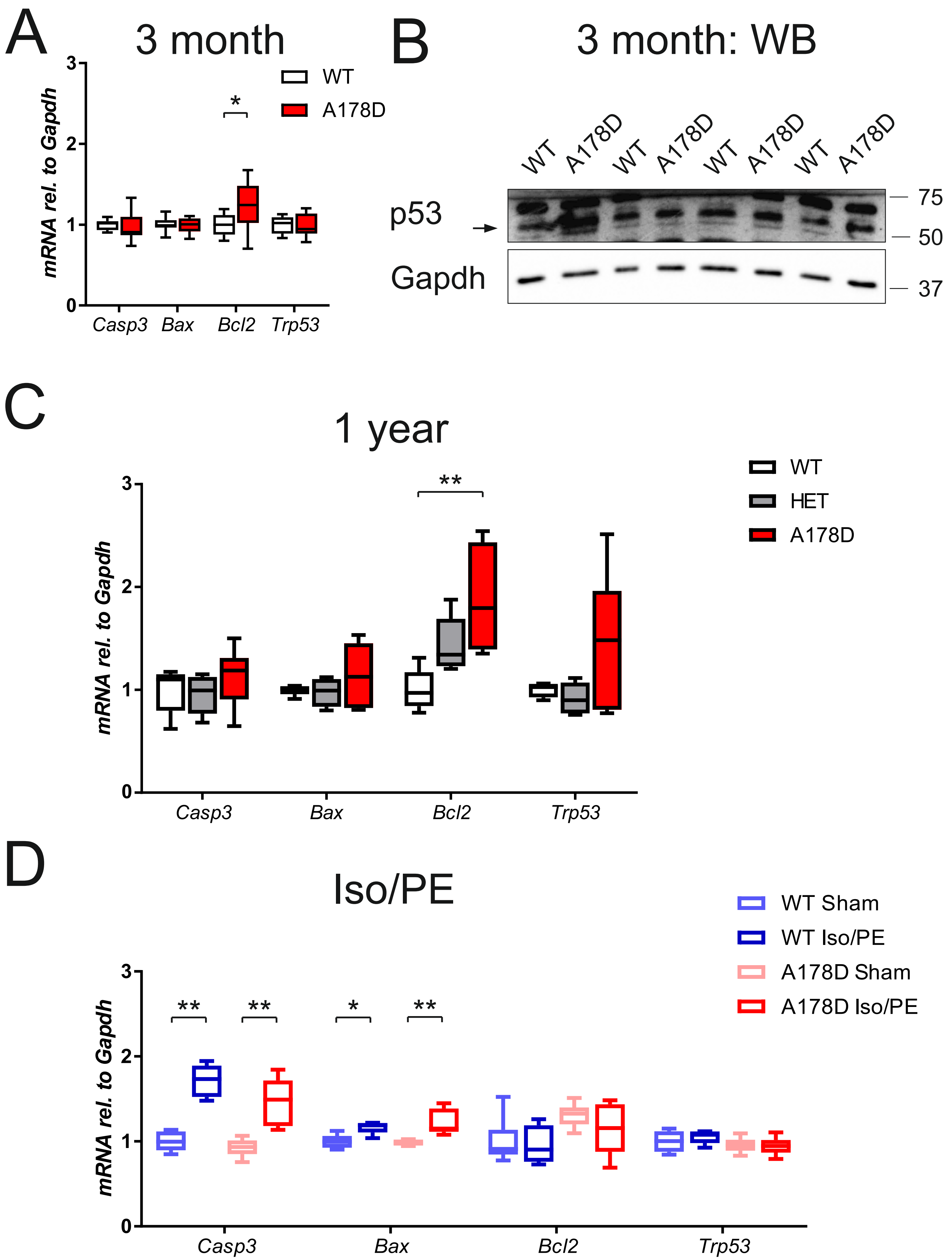

A
3 month
mRNA rel. to Gapdh
0
1
2
3
*
WT
A178D
Casp3
Bax
Bcl2
Trp53
B
3 month: WB
WT
A178D
WT
A178D
WT
A178D
WT
A178D
p53
Gapdh
75
50
37
C
1 year
mRNA rel. to Gapdh
0
1
2
3
**
WT
HET
A178D
Casp3
Bax
Bcl2
Trp53
D
Iso/PE
mRNA rel. to Gapdh
0
1
2
3
**
**
*
**
WT Sham
WT Iso/PE
A178D Sham
A178D Iso/PE
Casp3
Bax
Bcl2
Trp53




Figure S27

**Figure S28:** Assessment of transcriptional changes by qPCR for genes related to proteo-toxic response: A – Assessment in 3 month old hearts. All measurements are normalised to *Gapdh*; (Mann-Whitney U-test, n = 9 (4M/5F), age 114 ± 1 d). B – Assessment in 1 year old hearts. All measurements are normalised to *Gapdh*; Kruskal-Wallis followed by Dunn's multiple comparison's test; n = 6, all males, age WT 412 ± 2 d , HET 412 ± 2 d, A178D 423 ± 3 d. C – Assessment in the adrenergic challenge cohort. All measurements are normalised to *Gapdh*; (n = 6; age 120 ± 2 d, all males; Kruskal-Wallis followed by Wilcoxon rank sum test with Bonferroni correction). *Hspa1a* is shown separately on the right with a different scaling of the y-axis.

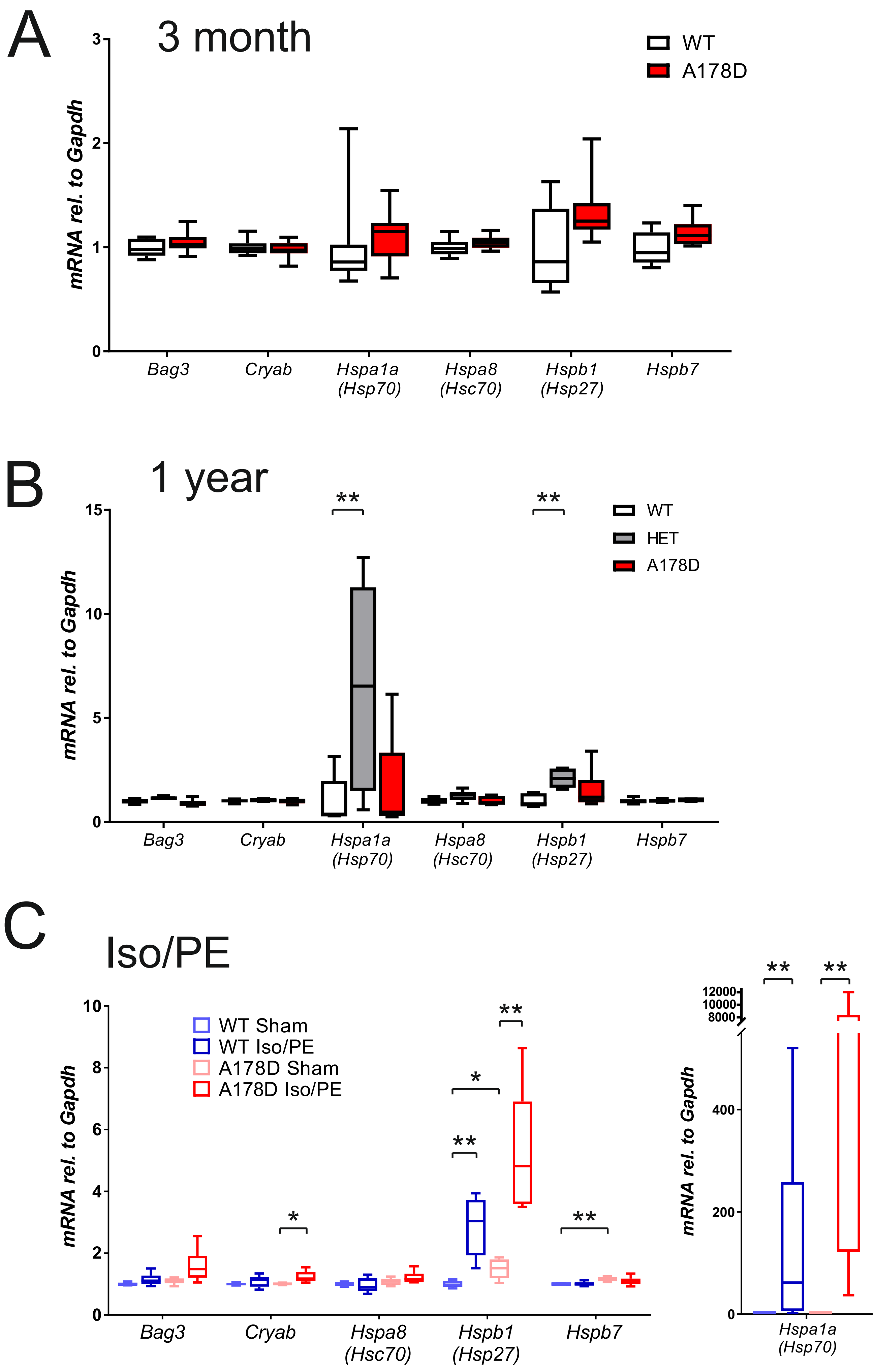
A
3 month
WT
A178D
mRNA rel. to Gapdh
3
2
1
0
Bag3
Cryab
Hspa1a (Hsp70)
Hspa8 (Hsc70)
Hspb1 (Hsp27)
Hspb7
B
1 year
**
**
WT
HET
A178D
15
10
5
0
C
Iso/PE
WT Sham
WT Iso/PE
A178D Sham
A178D Iso/PE
*
**
12000
10000
8000
400
200
Hspa1a (Hsp70)



Figure S28

**Figures S29-S34:** Assessment of changes for proteins related to a proteo-toxic response:

**Figure S29:** Analysis for Bag3 as in described in Figure S16.

**Figure S30:** Analysis for Hsp70 as in described in Figure S16.

**Figure S31:** Analysis for Hsc70 as in described in Figure S16.

**Figure S32:** Analysis for αβ-crystallin as in described in Figure S16.

**Figure S33:** Analysis for Hsp27 as in described in Figure S16.

**Figure S34:** Analysis for Hspb7 as in described in Figure S16.

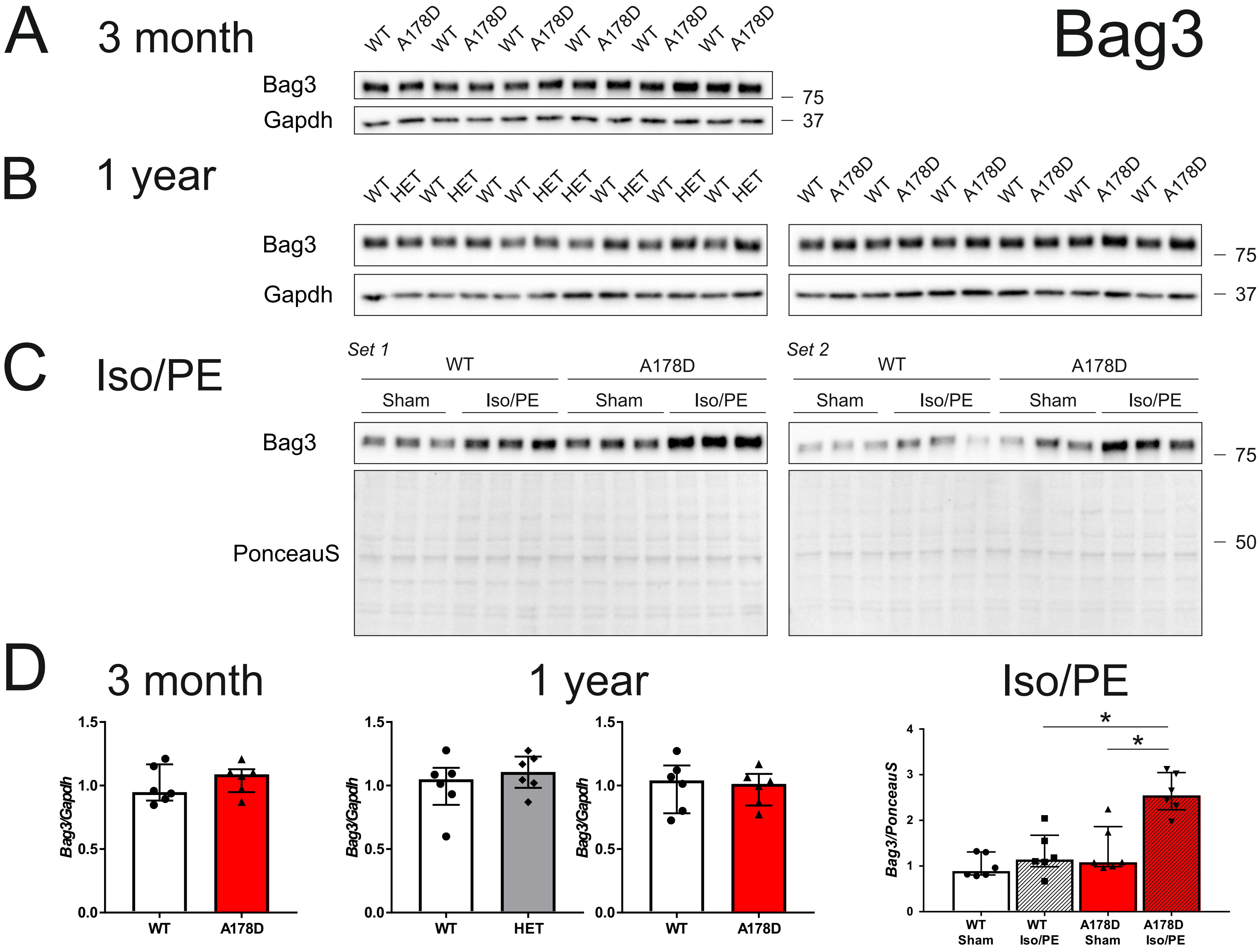

A
3 month
Bag3
WT A178D WT A178D WT A178D WT A178D WT A178D WT A178D
Bag3
Gapdh
75
37
B
1 year
WT HET WT HET WT WT HET HET WT HET WT HET WT HET
WT A178D WT A178D WT A178D WT A178D WT A178D WT A178D
Bag3
Gapdh
75
37
C
Iso/PE
Set 1
Set 2
WT
A178D
Sham
Iso/PE
Bag3
PonceauS
75
50
D
3 month
1 year
Iso/PE
Bag3/Gapdh
Bag3/PonceauS
WT
A178D
HET
WT Sham
WT Iso/PE
A178D Sham
A178D Iso/PE




Figure S29

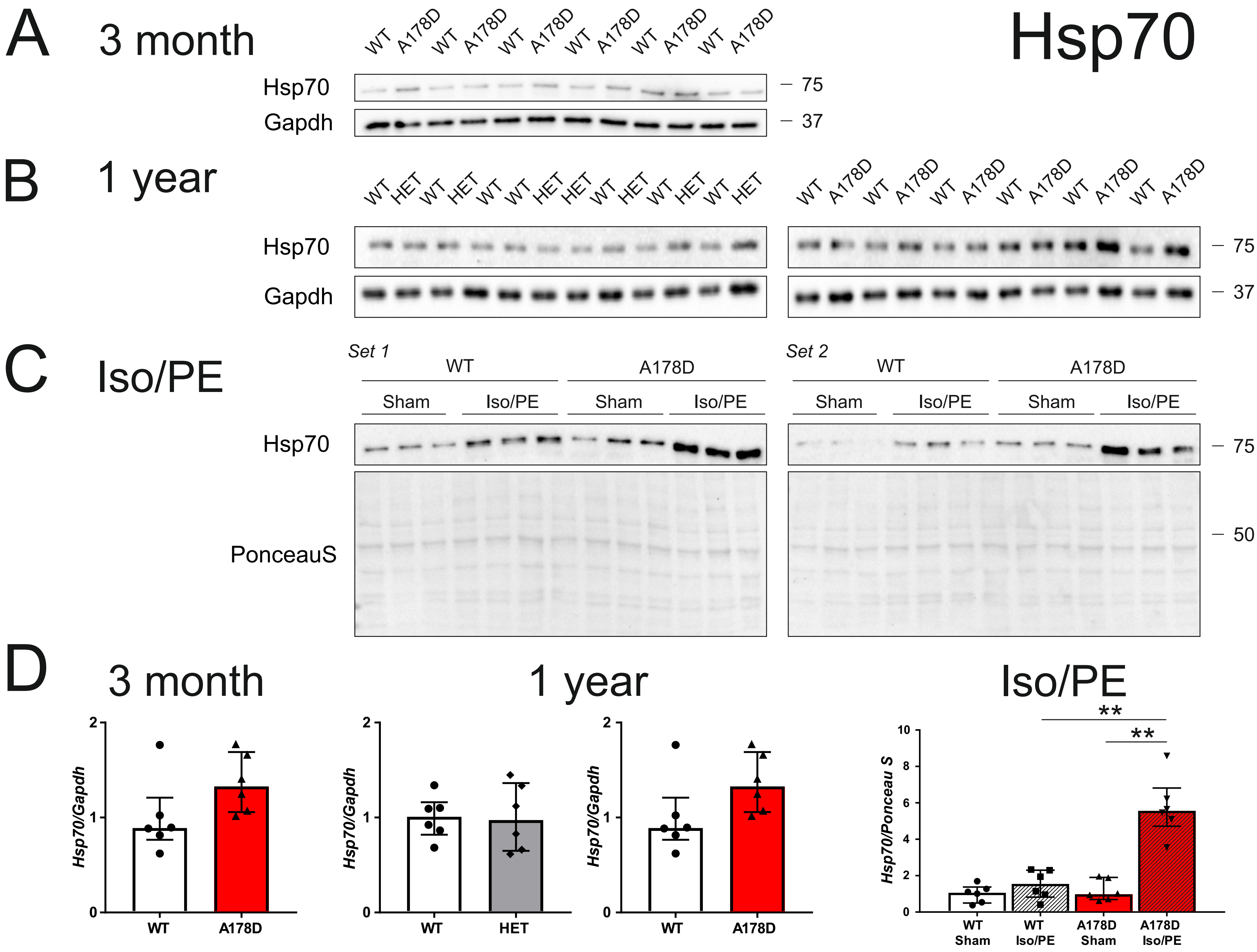

A
3 month
Hsp70
WT A178D WT A178D WT A178D WT A178D WT A178D WT A178D
Hsp70
Gapdh
75
37
B
1 year
WT HET WT HET WT WT HET HET WT HET WT HET WT HET
WT A178D WT A178D WT A178D WT A178D WT A178D WT A178D
Hsp70
Gapdh
75
37
C
Iso/PE
Set 1
Set 2
WT
A178D
Sham
Iso/PE
Hsp70
PonceauS
75
50
D
3 month
1 year
Iso/PE
Hsp70/Gapdh
Hsp70/Ponceau S
WT
A178D
HET
WT Sham
WT Iso/PE
A178D Sham
A178D Iso/PE
**




Figure S30

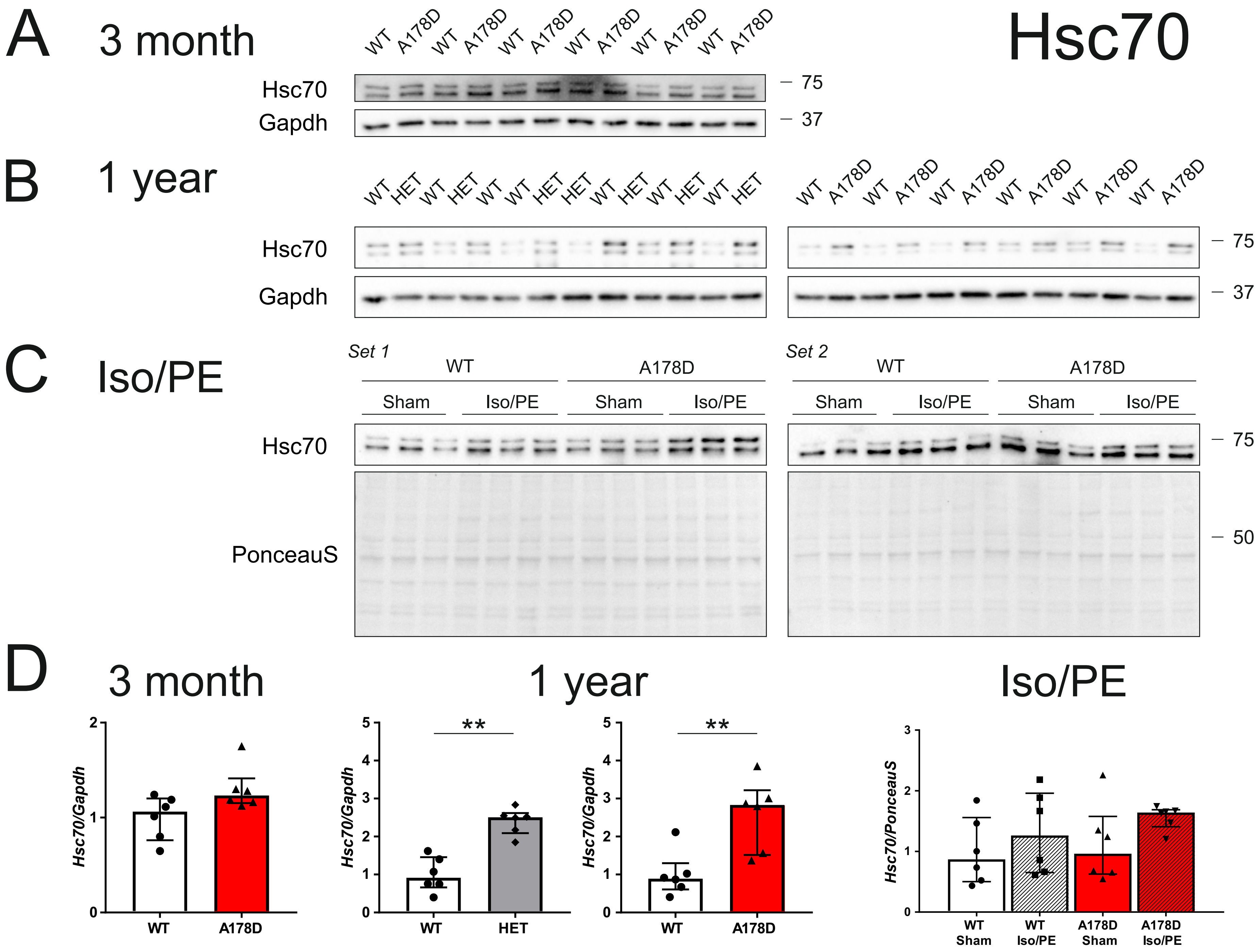
A
3 month
Hsc70
WT A178D WT A178D WT A178D WT A178D WT A178D WT A178D
Hsc70
Gapdh
75
37
B
1 year
WT HET WT HET WT WT HET HET WT HET WT HET WT HET
WT A178D WT A178D WT A178D WT A178D WT A178D WT A178D
Hsc70
Gapdh
75
37
C
Iso/PE
Set 1
Set 2
WT
A178D
Sham
Iso/PE
Hsc70
PonceauS
75
50
D
3 month
1 year
Iso/PE
Hsc70/Gapdh
Hsc70/PonceauS
WT
A178D
HET
**
WT Sham
WT Iso/PE
A178D Sham
A178D Iso/PE



Figure S31

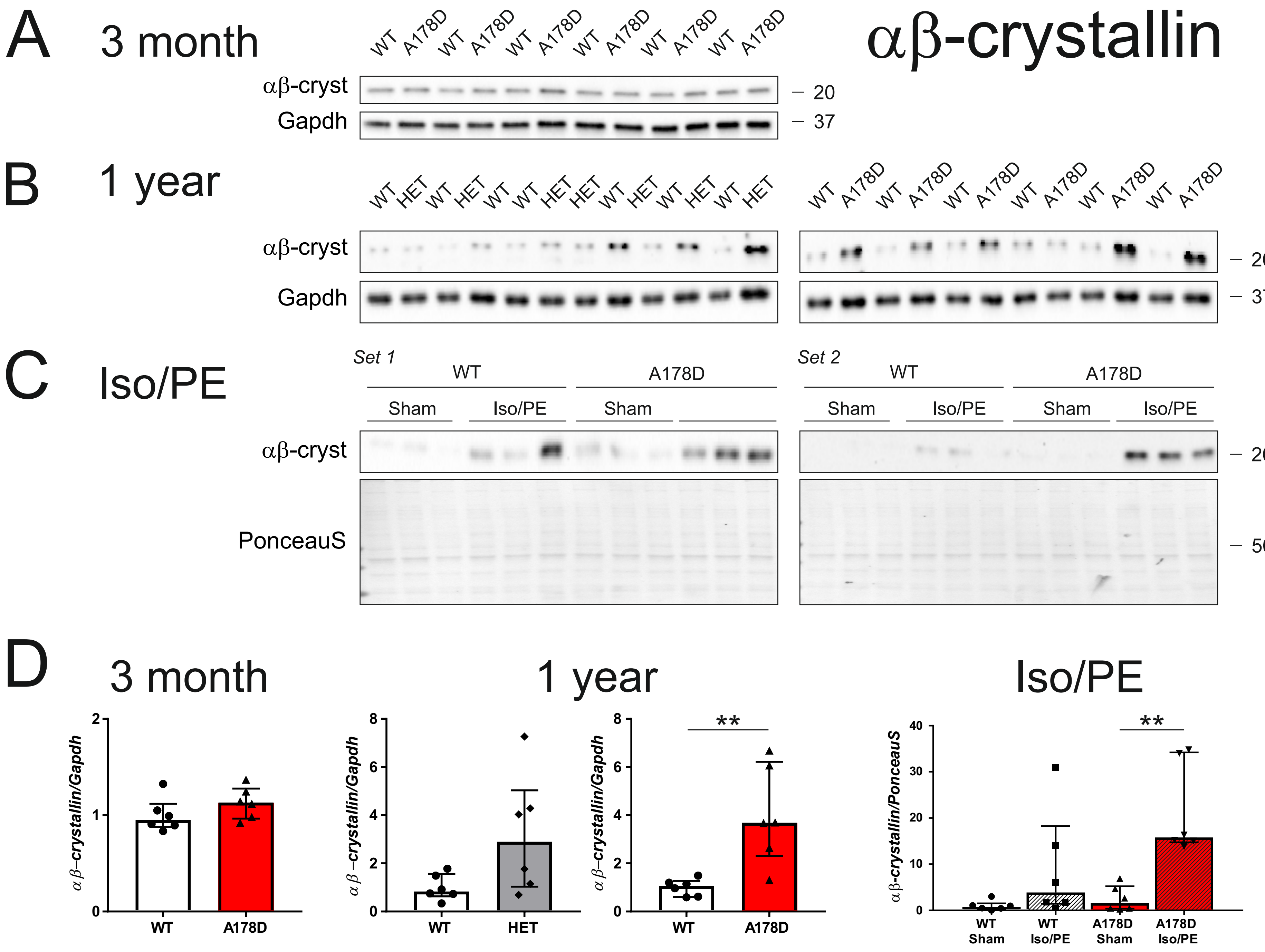

A
3 month
αβ-crystallin
WT A178D WT A178D WT A178D WT A178D WT A178D WT A178D
αβ-cryst
Gapdh
20
37
B
1 year
WT HET WT HET WT WT HET HET WT HET WT HET WT HET
WT A178D WT A178D WT A178D WT A178D WT A178D WT A178D
αβ-cryst
Gapdh
20
37
C
Iso/PE
Set 1
WT
A178D
Sham
Iso/PE
Sham
Set 2
WT
A178D
Sham
Iso/PE
Sham
Iso/PE
αβ-cryst
PonceauS
20
50
D
3 month
1 year
Iso/PE
αβ-crystallin/Gapdh
αβ-crystallin/PonceauS
WT
A178D
HET
**
WT Sham
WT Iso/PE
A178D Sham
A178D Iso/PE




Figure S32

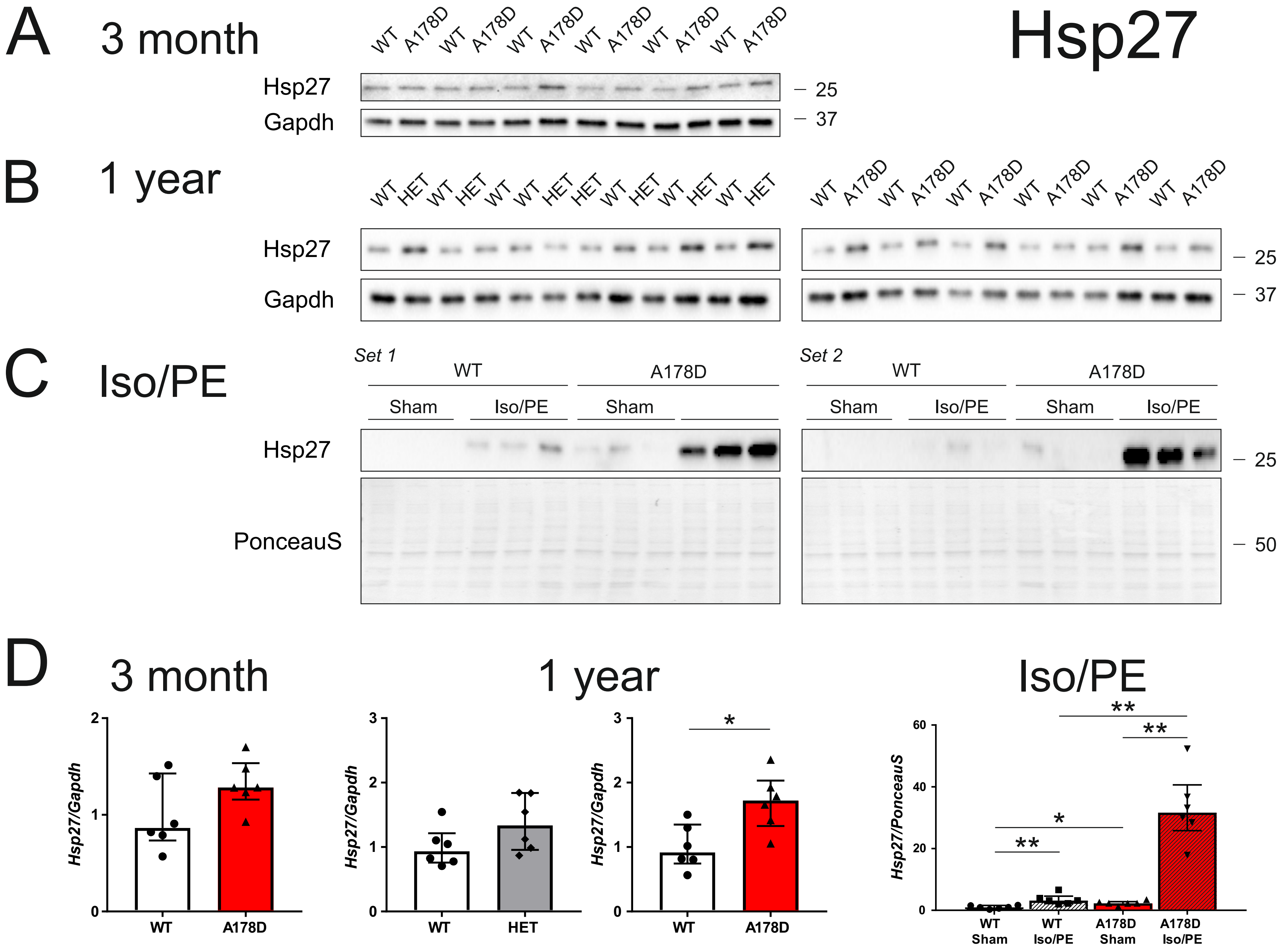
A
3 month
Hsp27
WT A178D WT A178D WT A178D WT A178D WT A178D WT A178D
Hsp27
Gapdh
25
37
B
1 year
WT HET WT HET WT WT HET HET WT HET WT HET WT HET
WT A178D WT A178D WT A178D WT A178D WT A178D WT A178D
Hsp27
Gapdh
25
37
C
Iso/PE
Set 1
WT
A178D
Sham
Iso/PE
Sham
Set 2
WT
A178D
Sham
Iso/PE
Sham
Iso/PE
Hsp27
PonceauS
25
50
D
3 month
1 year
Iso/PE
Hsp27/Gapdh
Hsp27/Gapdh
Hsp27/Gapdh
Hsp27/PonceauS
WT
A178D
WT
HET
WT
A178D
WT Sham
WT Iso/PE
A178D Sham
A178D Iso/PE
*
**
**
*
**



Figure S33

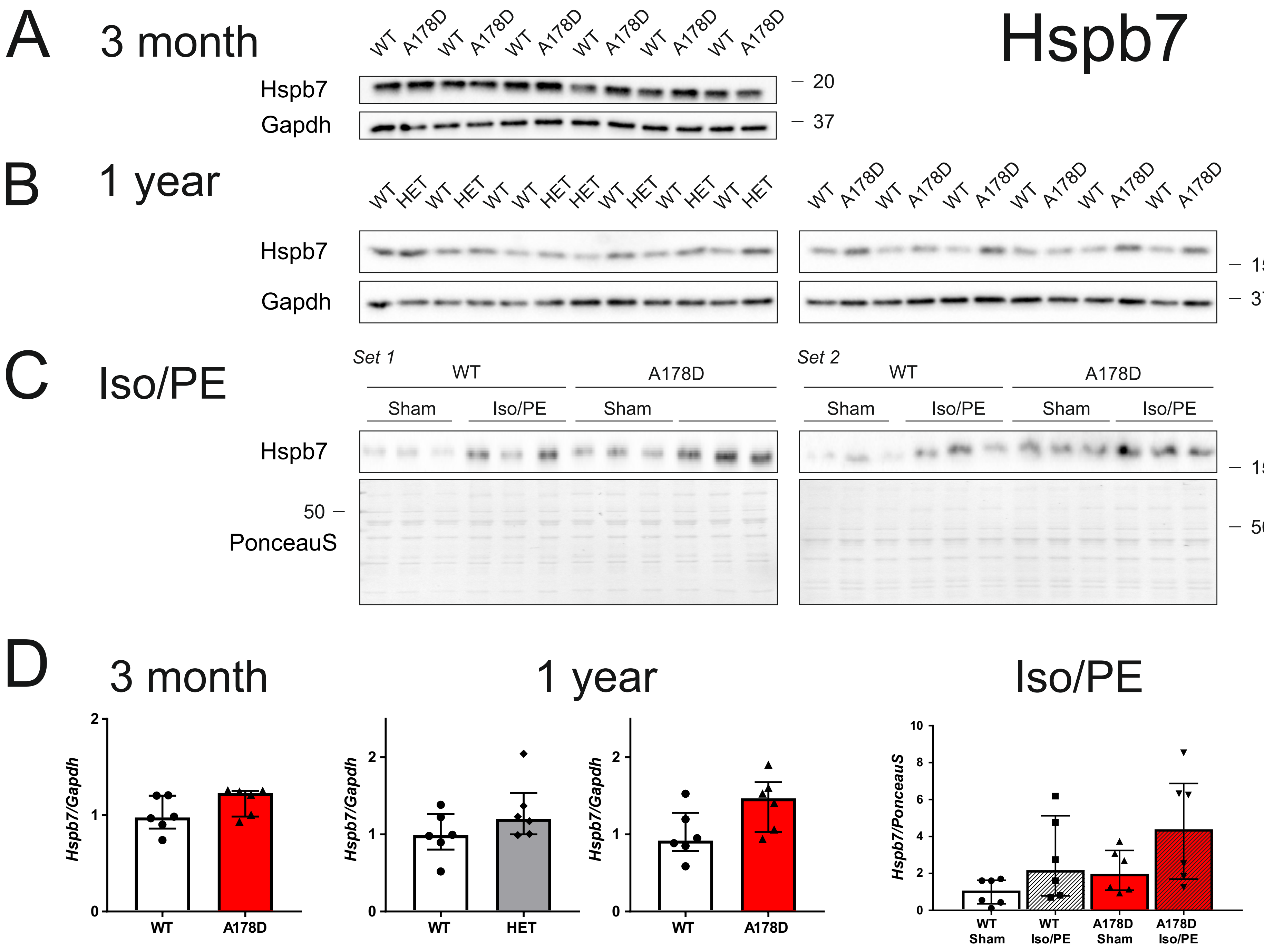
A
3 month
Hspb7
WT A178D WT A178D WT A178D WT A178D WT A178D WT A178D
Hspb7
Gapdh
20
37
B
1 year
WT HET WT HET WT WT HET HET WT HET WT HET WT HET
WT A178D WT A178D WT A178D WT A178D WT A178D WT A178D
Hspb7
Gapdh
15
37
C
Iso/PE
Set 1
WT
A178D
Sham
Iso/PE
Sham
Set 2
WT
A178D
Sham
Iso/PE
Sham
Iso/PE
Hspb7
15
50
PonceauS
50
D
3 month
1 year
Iso/PE
Hspb7/Gapdh
WT
A178D
HET
Hspb7/PonceauS
WT Sham
WT Iso/PE
A178D Sham
A178D Iso/PE



Figure S34

**Table S1**: Echocardiographic parameters of WT and *Ttn* A178D HET hearts at 3 and 6 months and 1 year. Values are given as mean ± SEM. No significant changes were observed between both genotypes at any age (Mann-Whitney-U-test at each time point, effect of aging not tested). FS – fractional shortening, LVEDD – left ventricular end diastolic dimension, LVESD - left ventricular end systolic dimension, LVAWD – left ventricular anterior wall in diastole, LVPWD – left ventricular posterior wall in diastole, LVM – calculated left ventricular mass, HR – heart rate, HW/TL – heart weight normalised to tibia length.

| | | **3 month** | | **6 month** | | **1 year** | |
|---|---|---|---|---|---|---|---|
| | | **WT** | **HET** | **WT** | **HET** | **WT** | **HET** |
| n | | 9 | 10 | 9 | 9 | 9 | 8 |
| Body weight | g | 29.0 ± 0.8 | 28.6 ± 0.6 | 36.3 ± 1.6 | 35.0 ± 1.4 | 44 ± 2 | 43 ± 2 |
| Age | days | 96 ± 1 | 97 ± 1 | 183 ± 1 | 184 ± 1 | 385 ± 1 | 386 ± 1 |
| FS | % | 41.0 ± 2.6 | 37.2 ± 1.8 | 40.3 ± 2.2 | 36.8 ± 2.2 | 32.2 ± 1.8 | 33.4 ± 0.9 |
| LVEDD | mm | 3.47 ± 0.04 | 3.68 ± 0.02 | 3.80 ± 0.08 | 3.77 ± 0.07 | 4.18 ± 0.08 | 4.19 ± 0.11 |
| LVESD | mm | 2.05 ± 0.08 | 2.32 ± 0.11 | 2.27 ± 0.10 | 2.39 ± 0.10 | 2.83 ± 0.10 | 2.79 ± 0.06 |
| LVAWD | mm | 1.08 ± 0.04 | 1.08 ± 0.02 | 1.15 ± 0.03 | 1.12 ± 0.04 | 1.09 ± 0.03 | 1.11 ± 0.04 |
| LVPWD | mm | 0.85 ± 0.02 | 0.85 ± 0.02 | 0.84 ± 0.02 | 0.95 ± 0.05 | 1.05 ± 0.06 | 1.02 ± 0.03 |
| LVM | mg | 98 ± 4 | 107 ± 4 | 118 ± 4 | 123 ± 5 | 152 ± 9 | 151 ± 6 |
| HR | bpm | 496 ± 2 | 497 ± 3 | 497 ± 4 | 496 ± 2 | 467 ± 14 | 461 ± 17 |
| HW/TL | mg/mm | nd | nd | nd | nd | 9.5 ± 0.3 | 9.5 ± 0.2 |

**Table S2:** Haemodynamic parameters of WT and *Ttn* A178D HET hearts at 1 year. Values are given as mean ± SEM. No significant differences were observed between the two genotypes at baseline or either given dobutamine dose (2-way-ANOVA, multiple comparisons only for genotype effect). LVESP – left ventricular end systolic pressure, LVEDP – left ventricular end diastolic pressure, AP – arterial pressure.

| | | **WT** | **HET** |
|---|---|---|---|
| n | | 8 | 7 |
| Body weight | g | 45.2 ± 2.2 | 46.5 ± 1.7 |
| Age | days | 424 ± 1 | 425 ± 2 |
| ***baseline*** | | | |
| Heart rate | (bpm) | 463 ± 18 | 471 ± 21 |
| $dP/dT_{max}$ | (mmHg/s) | 7693 ± 403 | 7063 ± 509 |
| $dP/dT_{min}$ | (mmHg/s) | -6561 ± 620 | -6422 ± 846 |
| Tau | (ms) | 9.3 ± 1.0 | 9.9 ± 1.1 |
| LVESP | (mmHg) | 100 ± 3 | 97 ± 4 |
| LVEDP | (mmHg) | 8.5 ± 1.6 | 7.8 ± 1.3 |
| Mean AP | (mmHg) | 44.4 ± 1.7 | 45.4 ± 2.3 |
| ***Dobutamine (low dose; 4 ng $g^{-1}$ BW $min^{-1}$)*** | | | |
| Heart rate | (bpm) | 479 ± 22 | 485 ± 20 |
| $dP/dT_{max}$ | (mmHg/s) | 8171 ± 486 | 7428 ± 516 |
| $dP/dT_{min}$ | (mmHg/s) | -6769 ± 604 | -6501 ± 790 |
| Tau | (ms) | 9.5 ± 1.0 | 9.7 ± 1.0 |
| $\Delta dP/dT_{max}$ | (mmHg/s) | 478 ± 209 | 365 ± 79 |
| ***Dobutamine (high dose; 16 ng $g^{-1}$ BW $min^{-1}$)*** | | | |
| Heart rate | (bpm) | 512 ± 21 | 501 ± 16 |
| $dP/dT_{max}$ | (mmHg/s) | 8955 ± 634 | 8772 ± 670 |
| $dP/dT_{min}$ | (mmHg/s) | -7041 ± 680 | -7031 ± 799 |
| Tau | (ms) | 9.2 ± 1.1 | 8.8 ± 0.9 |
| $\Delta dP/dT_{max}$ | (mmHg/s) | 1263 ± 341 | 1709 ± 320 |

**Table S3:** Echocardiographic parameters of WT and *Ttn* A178D hearts at 3 months. Values are given as mean ± SEM (Student's t test). For abbreviations see Legend Table S1.

| | | 3 month | | |
|---|---|---|---|---|
| | | **WT** | **A178D** | |
| n | | 21 | 17 | |
| Body weight | g | 29.0 ± 0.6 | 28.2 ± 0.7 | |
| Age | days | 98.7 ± 1.3 | 99.5 ± 1.3 | |
| FS | % | 44.5 ± 1.4 | 34.6 ± 1.3 | **** |
| LVEDD | mm | 3.79± 0.08 | 4.09 ± 0.07 | ** |
| LVESD | mm | 2.12 ± 0.09 | 2.69 ± 0.09 | *** |
| LVAWD | mm | 1.18 ± 0.04 | 1.09 ± 0.04 | |
| LVPWD | mm | 0.88 ± 0.02 | 0.83 ± 0.03 | |
| LVM | mg | 124 ± 5 | 126 ± 5 | |
| HR | bpm | 506.1 ± 1.1 | 503.9 ± 1.1 | |
| | | | | |
| HW/TL | mg/mm | 7.7 ± 0.15 | 7.6 ± 0.14 | |
| n | | 22 | 22 | |

**Table S4** Echocardiographic parameters of WT and *Ttn* A178D hearts (mean ± SEM) at 1 year. (Student's t-test, male mice). For abbreviations see Legend Table S1.

| | | 1 year | |
|---|---|---|---|
| | | **WT** | **A178D** |
| n | | 13 | 10 |
| age | days | 385 ± 4 | 391 ± 2 |
| Body weight | g | 41.2 ± 1.5 | 40.5 ± 1.9 |
| FS | % | 40.5 ± 1.2 | 37.7 ± 1.6 |
| LVEDD | mm | 3.88 ± 0.13 | 4.30 ± 0.16 * |
| LVESD | mm | 2.31 ± 0.10 | 2.69 ± 0.14 * |
| LVAWD | mm | 1.16 ± 0.05 | 1.09 ± 0.04 |
| LVPWD | mm | 1.11 ± 0.04 | 1.08 ± 0.07 |
| LVM | mg | 150 ± 12 | 160 ± 6 |
| HR | bpm | 500.4 ± 1.5 | 498.4 ± 2.0 |
| | | | |
| HW/TL | mg/mm | 8.75 ± 0.33 | 9.31 ± 0.32 |

**Table S5:** Echocardiographic parameters of WT and *Ttn* A178D hearts with Iso/PE or sham treatment in 3.5 month old mice. Values are given as mean ± SEM. $ p < 0.01 versus WT Iso/PE, * p < 0.05, ** p < 0.01,*** p < 0.001, **** p < 0.0001 versus sham group of same genotype (Kruskal-Wallis followed by Wilcoxon rank sum test with Bonferroni correction). For abbreviations see Legend Table S1.

| | | **WT sham** | **WT Iso/PE** | **A178D sham** | **A178D Iso/PE** |
|---|---|---|---|---|---|
| n | | 10 | 9 | 10 | 9 |
| Body weight | g | 28.5 ± 0.9 | 28.9 ± 0.4 | 27.7 ± 0.9 | 25.6 ± 0.6 $ |
| Age | days | 116.4 ± 1.3 | 117.1 ± 1.4 | 119.6 ± 1.4 | 117.1 ± 1.9 |
| FS | % | 39.8 ± 2.5 | 33.4 ± 3.2 | 33.9 ± 2.2 | 24.6 ± 2.0 * |
| LVEDD | mm | 3.96 ± 0.07 | 4.30 ± 0.16 | 4.12 ± 0.10 | 4.68 ± 0.12 ** |
| LVESD | mm | 2.39 ± 0.12 | 2.89 ± 0.22 | 2.74 ± 0.15 | 3.54 ± 0.15 ** |
| LVAWD | mm | 1.13 ± 0.02 | 1.17 ± 0.04 | 1.03 ± 0.03 | 1.06 ± 0.05 |
| LVPWD | mm | 0.89 ± 0.02 | 1.08 ± 0.04* | 0.85 ± 0.03 | 0.97 ± 0.04 |
| LVM | mg | 128 ± 4 | 171 ± 12 ** | 123 ± 5 | 169 ± 9 * |
| HR | bpm | 504 ± 1 | 502 ± 3 | 504 ± 2 | 501 ± 2 |
| HW/TL | mg/mm | 8.00 ± 0.16 | 11.7 ±0.3 *** | 7.99 ± 0.15 | 10.9 ± 0.3 *** |

**Table S6:** Cell dimension and contractility measurements of unloaded cardiomyocytes isolated from WT and *Ttn* A178D mice (mean ± SEM). Analysed by Student's t-test using hierarchical clustering (see methods).

| | **WT** | **A178D** | |
|---|---|---|---|
| Total number of mice | 12 | 12 | |
| Number of female mice | 4 | 4 | |
| Number of male mice | 8 | 8 | |
| Age of mice (days) | 128 ± 8 | 132 ± 10 | |
| | | | |
| ***Cell size*** | | | |
| Number of cells per mouse | 32-39 | 26-32 | |
| Total number of cells | 392 | 369 | |
| Area (µm$^2$) | 3161 ± 123 | 4171 ± 124 | **** |
| Length (µm) | 119 ± 4 | 138 ± 4 | ** |
| Width (µm) | 34.5 ± 1.0 | 39.7 ± 1.0 | *** |
| Length:Width ratio | 3.67 ± 0.18 | 3.74 ± 0.18 | |
| | | | |
| ***Contractility*** | | | |
| Number of cells per mouse | 4-25 | 10-27 | |
| Total number of cells | 197 | 215 | |
| Basal sarcomere length (µm) | 1.88 ± 0.01 | 1.86 ± 0.01 | |
| Fractional sacromere shortening (%) | 5.6 ± 0.2 | 5.6 ± 0.2 | |
| Time to 50% peak contraction (ms) | 26.9 ± 0.7 | 25.7 ± 0.7 | |
| Time to 90% peak contraction (ms) | 43.5 ± 1.3 | 43.0 ± 1.2 | |
| Time to 50% relaxation (ms) | 30.0 ± 1.5 | 34.0 ± 1.5 | |

**Table S7:** Calcium transient measurements of Fura-2 loaded cardiomyocytes isolated from WT and *Ttn* A178D mice (mean ± SEM). Analysed by Student's t-test using hierarchical clustering (see methods).

| | **WT** | **A178D** |
|---|---|---|
| Total number of mice | 5 | 5 |
| Number of female mice | 2 | 2 |
| Number of male mice | 3 | 3 |
| Age of mice (days) | 147 ± 7 | 159 ± 10 |
| ***Calcium transient measurements*** | | |
| Total number of cells | 63 | 59 |
| Diastolic calcium (Fura-2 ratio) | 0.884 ± 0.008 | 0.842 ± 0.009 |
| Calcium transient amplitude (Fura-2 ratio) | 0.331 ± 0.016 | 0.314 ± 0.013 |
| Time decay constant Tau (msec) | 85 ± 3 | 79 ± 2 |

**Table S8:** Passive tension measurements in loaded, demembranised fibres from WT and A178D hearts. Passive tension (mN/mm$^2$) and titin derived fraction of total passive tension were assessed; the remaining tension was attributed to extracellular collagen. Titin derived fraction of total passive tension was calculated, all values given as mean ± SEM.

All parameter were found to be normal (Student's t-test; n = 6, age WT 132 ± 2 d/A178D 129 ± 1 d, 4M/2F).

| | **Stretch** | **Passive tension** mN/mm$^2$ | **titin derived passive tension** mN/mm$^2$ | **collagen derived passive tension** mN/mm$^2$ | **titin fraction** |
|---|---|---|---|---|---|
| **WT** | 1.05 $L_0$ | 3.7 ± 0.69 | 3.0 ± 0.72 | 0.73 ± 0.06 | 0.76 ± 0.06 |
| **A178D** | 1.05 $L_0$ | 5.5 ± 2.2 | 4.7 ± 2.0 | 0.79 ± 0.26 | 0.83 ± 0.02 |
| **WT** | 1.1 $L_0$ | 8.6 ± 1.6 | 6.3 ± 1.6 | 2.2 ± 0.15 | 0.70 ± 0.06 |
| **A178D** | 1.1 $L_0$ | 11.1 ± 3.9 | 8.6 ± 3.2 | 2.5 ± 0.71 | 0.76 ± 0.02 |
| **WT** | 1.15 $L_0$ | 17.3 ± 3.2 | 10.3 ± 2.5 | 7.0 ± 1.0 | 0.57 ± 0.05 |
| **A178D** | 1.15 $L_0$ | 18.6 ± 4.4 | 11.3 ± 3.0 | 7.3 ± 1.4 | 0.59 ± 0.02 |
| **WT** | 1.2 $L_0$ | 31.9 ± 6.2 | 11.2 ± 3.3 | 20.7 ± 3.5 | 0.33 ± 0.06 |
| **A178D** | 1.2 $L_0$ | 31.4 ± 4.2 | 10.7 ± 1.8 | 20.7 ± 2.4 | 0.33 ± 0.02 |

**Table S9:** Genes differentially expressed in *Ttn* A178D compared to WT hearts in RNAseq analysis (FDR < 0.05, n = 3/4 (WT/A178D), age 135 ± 1 d, all male). Table available as extra file.

**Table S10:** KEGG pathway gene sets significantly enriched in either A178D or WT using the UC San Diego and Broad Institute GSEA software. n = 3/4 (WT/A178D), age 135 ± 1 d, all male; ES – enrichment score, NES – normalised enrichment score, FDR – false discovery rate, FWER – Familywise-error rate.

| **Gene set** | **ES** | **NES** | **FDR q-value** | **FWER p-value** | **Rank at max** |
|---|---|---|---|---|---|
| **Enriched in A178D** | | | | | |
| KEGG_PROTEASOME | 0.64 | 2.21 | 0.000 | 0.000 | 4947 |
| KEGG_OXIDATIVE_PHOSPHORYLATION | 0.46 | 1.86 | 0.033 | 0.064 | 5300 |
| KEGG_BUTANOATE_METABOLISM | 0.59 | 1.83 | 0.030 | 0.087 | 1992 |
| KEGG_RIBOSOME | 0.47 | 1.80 | 0.034 | 0.130 | 5821 |
| **Enriched in WT** | | | | | |
| KEGG_VASCULAR_SMOOTH_MUSCLE_CONTRACTION | -0.49 | -2.06 | 0.004 | 0.004 | 1804 |

**Table S11:** Reagents used for qPCR, Western blotting (WB) and immuno-fluorescence (IF).

a) Taqman Assays for qPCR

(all Applied Biosystems, FAM-MGB unless stated otherwise)

| ***Transcript*** | ***Species*** | ***Assay ID*** | ***Comments*** |
|---|---|---|---|
| Acta1 | Mouse | Mm00808218_g1 | |
| Actn2 | Mouse | Mm00473657_m1 | |
| Ankrd1 | Mouse | Mm00496512_m1 | |
| Ankrd2 | Mouse | Mm00508030_m1 | |
| Bag3 | Mouse | Mm00443474_m1 | |
| Bax | Mouse | Mm00432051_m1 | |
| Bcl2 | Mouse | Mm00477631_m1 | |
| Casp3 | Mouse | Mm01195085_m1 | |
| Col1a1 | Mouse | Mm00801666_g1 | |
| Cryab | Mouse | Mm00515567_m1 | (αβ-CRYSTALLIN) |
| Csrp3 | Mouse | Mm00443379_m1 | (CSRP3/MLP) |
| Fhl1 | Mouse | Mm04204611_g1 | |
| Fhl2 | Mouse | Mm00515781_m1 | |
| Gapdh | Mouse | 4352339E | VIC-MGB labelled |
| Hspa1a | Mouse | Mm01159846_s1 | (HSP70) |
| Hspa8 | Mouse | Mm01731394_gH | (HSC70) |
| Hspb1 | Mouse | Mm00834384_g1 | (HSP27) |
| Hspb7 | Mouse | Mm04210487_m1 | |
| Lox | Mouse | Mm00495386_m1 | |
| Myh7 | Mouse | Mm00600555_m1 | |
| Nppa | Mouse | Mm01255748_g1 | |
| Nppb | Mouse | Mm01255770_g1 | |
| Rcan1 | Mouse | Mm00627762_m1 | |
| Trp53 | Mouse | Mm01731290_g1 | |
| Tcap | Mouse | Mm00495557_g1 | |
| Ttn | Mouse | Mm00621005_m1 | |

b) Antibodies (Dilutions for Western blotting unless stated. IF - Immunofluorescence)

| ***Protein*** | ***Type*** | ***Clone/Cat. No.*** | ***Source*** | ***Dilution (WB)*** |
|---|---|---|---|---|
| αβ-crystallin | mouse | ab13496 | Abcam | 1:3000 |
| alpha-actinin | rabbit | ab68167 | Abcam | 1:1000 |
| alpha-actinin | mouse | EA53/ A7811 | Sigma | 1:500 (IF) |
| Ankrd1/Carp | rabbit | ab88456 | Abcam | 1:500 |
| Bag3 | rabbit | 10599-1-AP | Proteintech | 1:2000 |
| beta-myosin | mouse | NOQ7.5.4D/M8421 | Sigma | 1:2000 |
| Fhl1 | mouse | ab76912 | Abcam | 1:200 |
| Fhl2 | mouse | K0055-3 | MBL | 1:500, 1:50 (IF) |
| Gapdh | rabbit | ABS16 | Millipore | 1:3000 |
| Hsc70 | rabbit | ADI-SPA-815-D | Enzo | 1:1000 |
| Hsp70 | rabbit | 4872 | CST | 1:1000 |
| Hsp27 | rabbit | 2442 | CST | 1:1000 |
| Hspb7 | rabbit | 15700-1-AP | Proteintech | 1:1000 |
| Laminin | rabbit | L9393 | Sigma | 1:200 (IF) |
| LC3 | rabbit | 2775 | CST | 1:250 |
| Mlf1 | rabbit | 13100-1-AP | Proteintech | 1:500 |
| Csrp3/MLP | mouse | 79D2 | [8] | 1:1000 |
| myomesin | mouse | B4 | DSHB | 1:200 (IF) |
| p53 | mouse | DO-7 | DAKO | 1:1000 |
| p62 | mouse | ab56416 | Abcam | 1:500 |
| telethonin | mouse | sc-25327 | Santa Cruz | 1:30 (IF) |
| telethonin | rabbit | ab133646 | Abcam | 1:1000, 1:30 (IF) |
| titin m8 | rabbit | m8 (M-band) | [17] | 1:50 (IF) |
| titin T12 | mouse | T12 (near Z-disc) | [5] | 1:10 (IF) |
| titin Z1Z2 | rabbit | Z1Z2 (Z-disc) | [9] | 1:50 (IF) |
| ubiquitin | rabbit | Z0458 | DAKO | 1:1000 |

## Supplementary References


1. Agarkova I, Schoenauer R, Ehler E, Carlsson L, Carlsson E, Thornell LE, Perriard JC (2004) The molecular composition of the sarcomeric M-band correlates with muscle fiber type. Eur J Cell Biol 83:193-204 doi:10.1078/0171-9335-00383

2. Captur G, Radenkovic D, Li C, Liu Y, Aung N, Zemrak F, Tobon-Gomez C, Gao X, Elliott PM, Petersen SE, Bluemke DA, Friedrich MG, Moon JC (2017) Community delivery of semiautomated fractal analysis tool in cardiac mr for trabecular phenotyping. J Magn Reson Imaging 46:1082-1088 doi:10.1002/jmri.25644

3. Carnicer R, Hale AB, Suffredini S, Liu X, Reilly S, Zhang MH, Surdo NC, Bendall JK, Crabtree MJ, Lim GB, Alp NJ, Channon KM, Casadei B (2012) Cardiomyocyte GTP cyclohydrolase 1 and tetrahydrobiopterin increase NOS1 activity and accelerate myocardial relaxation. Circ Res 111:718-727 doi:10.1161/CIRCRESAHA.112.274464

4. Chung CS, Granzier HL (2011) Contribution of titin and extracellular matrix to passive pressure and measurement of sarcomere length in the mouse left ventricle. J Mol Cell Cardiol 50:731-739 doi:10.1016/j.yjmcc.2011.01.005

5. Furst DO, Osborn M, Nave R, Weber K (1988) The organization of titin filaments in the half-sarcomere revealed by monoclonal antibodies in immunoelectron microscopy: a map of ten nonrepetitive epitopes starting at the Z line extends close to the M line. J Cell Biol 106:1563-1572 doi:10.1083/jcb.106.5.1563

6. Gehmlich K, Dodd MS, Allwood JW, Kelly M, Bellahcene M, Lad HV, Stockenhuber A, Hooper C, Ashrafian H, Redwood CS, Carrier L, Dunn WB (2015) Changes in the

cardiac metabolome caused by perhexiline treatment in a mouse model of hypertrophic cardiomyopathy. Mol Biosyst 11:564-573 doi:10.1039/c4mb00594e

7. Gehmlich K, Pinotsis N, Hayess K, van der Ven PF, Milting H, El Banayosy A, Körfer R, Wilmanns M, Ehler E, Fürst DO (2007) Paxillin and ponsin interact in nascent costameres of muscle cells. J Mol Biol 369:665-682 doi:10.1016/j.jmb.2007.03.050

8. Geier C, Gehmlich K, Ehler E, Hassfeld S, Perrot A, Hayess K, Cardim N, Wenzel K, Erdmann B, Krackhardt F, Posch MG, Osterziel KJ, Bublak A, Nägele H, Scheffold T, Dietz R, Chien KR, Spuler S, Fürst DO, Nürnberg P, Ozcelik C (2008) Beyond the sarcomere: CSRP3 mutations cause hypertrophic cardiomyopathy. Hum Mol Genet 17:2753-2765 doi:10.1093/hmg/ddn160

9. Gregorio CC, Trombitas K, Centner T, Kolmerer B, Stier G, Kunke K, Suzuki K, Obermayr F, Herrmann B, Granzier H, Sorimachi H, Labeit S (1998) The NH2 terminus of titin spans the Z-disc: its interaction with a novel 19-kD ligand (T-cap) is required for sarcomeric integrity. J Cell Biol 143:1013-1027 doi:10.1083/jcb.143.4.1013

10. Hastings R, de Villiers CP, Hooper C, Ormondroyd L, Pagnamenta A, Lise S, Salatino S, Knight SJ, Taylor JC, Thomson KL, Arnold L, Chatziefthimiou SD, Konarev PV, Wilmanns M, Ehler E, Ghisleni A, Gautel M, Blair E, Watkins H, Gehmlich K (2016) Combination of Whole Genome Sequencing, Linkage, and Functional Studies Implicates a Missense Mutation in Titin as a Cause of Autosomal Dominant Cardiomyopathy With Features of Left Ventricular Noncompaction. Circ Cardiovasc Genet 9:426-435 doi:10.1161/circgenetics.116.001431

11. Hirschy A, Schatzmann F, Ehler E, Perriard JC (2006) Establishment of cardiac cytoarchitecture in the developing mouse heart. Dev Biol 289:430-441 doi:10.1016/j.ydbio.2005.10.046

12. Hutchinson KR, Saripalli C, Chung CS, Granzier H (2015) Increased myocardial stiffness due to cardiac titin isoform switching in a mouse model of volume overload limits eccentric remodeling. J Mol Cell Cardiol 79:104-114 doi:10.1016/j.yjmcc.2014.10.020

13. Lange S, Gehmlich K, Lun AS, Blondelle J, Hooper C, Dalton ND, Alvarez EA, Zhang X, Bang ML, Abassi YA, Dos Remedios CG, Peterson KL, Chen J, Ehler E (2016) MLP and CARP are linked to chronic PKCalpha signalling in dilated cardiomyopathy. Nat Commun 7:12120 doi:10.1038/ncomms12120

14. Li H, Handsaker B, Wysoker A, Fennell T, Ruan J, Homer N, Marth G, Abecasis G, Durbin R, Genome Project Data Processing S (2009) The Sequence Alignment/Map format and SAMtools. Bioinformatics 25:2078-2079 doi:10.1093/bioinformatics/btp352

15. Love MI, Huber W, Anders S (2014) Moderated estimation of fold change and dispersion for RNA-seq data with DESeq2. Genome Biol 15:550 doi:10.1186/s13059-014-0550-8

16. Neagoe C, Kulke M, del Monte F, Gwathmey JK, de Tombe PP, Hajjar RJ, Linke WA (2002) Titin isoform switch in ischemic human heart disease. Circulation 106:1333-1341 doi:10.1161/01.cir.0000029803.93022.93

17. Obermann WM, Gautel M, Steiner F, van der Ven PF, Weber K, Furst DO (1996) The structure of the sarcomeric M band: localization of defined domains of myomesin, M-protein, and the 250-kD carboxy-terminal region of titin by immunoelectron microscopy. J Cell Biol 134:1441-1453 doi:10.1083/jcb.134.6.1441

18. Perez-Riverol Y, Csordas A, Bai J, Bernal-Llinares M, Hewapathirana S, Kundu DJ, Inuganti A, Griss J, Mayer G, Eisenacher M, Perez E, Uszkoreit J, Pfeuffer J, Sachsenberg T, Yilmaz S, Tiwary S, Cox J, Audain E, Walzer M, Jarnuczak AF, Ternent T, Brazma A, Vizcaino JA (2019) The PRIDE database and related tools and resources in 2019: improving support for quantification data. Nucleic Acids Res 47:D442-D450 doi:10.1093/nar/gky1106

19. Smart N, Riegler J, Turtle CW, Lygate CA, McAndrew DJ, Gehmlich K, Dube KN, Price AN, Muthurangu V, Taylor AM, Lythgoe MF, Redwood C, Riley PR (2017) Aberrant developmental titin splicing and dysregulated sarcomere length in Thymosin beta4 knockout mice. J Mol Cell Cardiol 102:94-107 doi:10.1016/j.yjmcc.2016.10.010

20. Subramanian A, Tamayo P, Mootha VK, Mukherjee S, Ebert BL, Gillette MA, Paulovich A, Pomeroy SL, Golub TR, Lander ES, Mesirov JP (2005) Gene set enrichment analysis: a knowledge-based approach for interpreting genome-wide expression profiles. Proc Natl Acad Sci U S A 102:15545-15550 doi:10.1073/pnas.0506580102

21. Tyanova S, Temu T, Sinitcyn P, Carlson A, Hein MY, Geiger T, Mann M, Cox J (2016) The Perseus computational platform for comprehensive analysis of (prote)omics data. Nat Methods 13:731-740 doi:10.1038/nmeth.3901
